# Bayesian compositional regression with microbiome features via variational inference

**DOI:** 10.1186/s12859-023-05219-x

**Published:** 2023-05-22

**Authors:** Darren A. V. Scott, Ernest Benavente, Julian Libiseller-Egger, Dmitry Fedorov, Jody Phelan, Elena Ilina, Polina Tikhonova, Alexander Kudryavstev, Julia Galeeva, Taane Clark, Alex Lewin

**Affiliations:** 1 Department of Medical Statistics, London School of Hygiene and Tropical Medicine, Keppel Street, London, United Kingdom; 2 Laboratory of Experimental Cardiology, University Medical Center Utrecht, Utrecht University, Utrecht, Netherlands; 3 Federal Research and Clinical Center of Physical-Chemical Medicine, Moscow, Russia; 4 Bioinformatics and Genomics Intercollege Graduate Program, Huck Institutes of Life Sciences, Pennsylvania State University, Pennsylvania, USA; 5 Northern State Medical University, Arkhangelsk, Russia

**Keywords:** Compositional, Variational inference, Microbiome, Singular multivariate normal, Markov chain Monte Carlo

## Abstract

The microbiome plays a key role in the health of the human body. Interest often lies in finding features of the microbiome, alongside other covariates, which are associated with a phenotype of interest. One important property of microbiome data, which is often overlooked, is its compositionality as it can only provide information about the relative abundance of its constituting components. Typically, these proportions vary by several orders of magnitude in datasets of high dimensions. To address these challenges we develop a Bayesian hierarchical linear log-contrast model which is estimated by mean field Monte-Carlo co-ordinate ascent variational inference (CAVI-MC) and easily scales to high dimensional data. We use novel priors which account for the large differences in scale and constrained parameter space associated with the compositional covariates. A reversible jump Monte Carlo Markov chain guided by the data through univariate approximations of the variational posterior probability of inclusion, with proposal parameters informed by approximating variational densities via auxiliary parameters, is used to estimate intractable marginal expectations. We demonstrate that our proposed Bayesian method performs favourably against existing frequentist state of the art compositional data analysis methods. We then apply the CAVI-MC to the analysis of real data exploring the relationship of the gut microbiome to body mass index.

**Supplementary Information:**

The online version contains supplementary material available at 10.1186/s12859-023-05219-x.

## Introduction

The human microbiome is the combined genome of the microorganisms that live in the human body. It has been estimated that these microbes make up to 10 trillion cells, equivalent to the number of human cells [1]. Advances in genome sequencing technologies has enabled scientists to study these microbes and their function and to research microbiome-host interactions both in health and disease. The decreasing cost and increasing accessibility of nucleotide sequencing means it is the primary tool used to study the microbiome [2]. Any microbiome dataset is compositional [3] as the magnitude of a single operational taxonomic unit (OTU) depends on the sum of all the OTUs counts, and only provides information about the relative magnitudes of the compositional components. This means that the standard methods of analysis such as linear regression are not applicable to microbiome data [4], unless a transformation is performed.

The large dimensions of these datasets often present a problem in variable selection where the number of covariates *p* exceeds the number of observations *n* ($p>> n$) and the space of possible combinations of significant variables is large, imposing a high computational burden. Sparse variable selection of the *p* covariates is expected, where just a few microbes are associated with the response. Bayesian variable selection approaches have the advantage of being able to include prior knowledge and simultaneously incorporate many sources of variation. Shrinkage priors encourage the majority of regression coefficients to be shrunk to very small values when an estimator is applied identifying associations [5]. Alternatively, introducing latent variables produces posterior distributions of model inclusion and parameter values which enable model choice and a probabilistic understanding of the strength and nature of the association [6]. The different approaches within explicit variable selection are characterised by the location of the latent variable and its relationship with the covariates ([7–9]).

To model compositional data, a transformation is required to transfer the compositional vectors into Euclidean space. Various log-ratio transformations have been proposed including additive log-ratio (alr), centred log-ratio (clr) [10] and more recently isometric log-ratio (ilr) [11]. The ilr transformation defines balances proportional to the log difference between two groups which are scale invariant. In ilr linear regression models, just the first parameter can be interpreted. Thus, the only way to interpret the role of *d* compositional parts for explaining the response is to consider *d* different regression models [12].

In the context of regression, the reparameterised alr transformation (or log-contrast model) removes the requirement for a reference category and results in a sum to zero constraint on the associated parameter space within the linear model, has proved to be useful in allowing a direct inference between selected covariates and the compositional data set [13, 14] propose an adaptive $l_1$ regularisation regression for the log-contrast lasso. This has been extended to multiple linear constraints for sub-compositional coherence across predefined groups of predictors [15]. To obtain a sparser model [16] introduce an additional feature selection step on those variables identified in a two-step log-ratio lasso. A general approach to convex optimisation, where the model has been extended to the high-dimensional setting via regularization has recently been proposed by [17]. In the Bayesian framework [18] introduce a generalised transformation matrix on the parameters rather than the covariates, as a function of a tuning parameter *c*, similar to the generalized lasso. This ensures parameter estimates remain in the *p* space and as *c* reaches infinity the sum to zero constraint is imposed. By incorporating the matrix into conjugate prior and avoiding any singular distributions by not strictly imposing the zero sum constraint, a Gibbs sampler for the marginal posterior of the selection parameter can be derived. Alternative Bayesian approaches treat the the microbiome predictors as random, parameterised by a multivariate count model. [19] combine this with the ilr transformation in a predictive model which identifies correlations across the microbiome. [20] cluster on a categorical covariate via a Gaussian mixture model in an ANOVA type model, but both approaches do not allow a direct inference between the compositional predictors and the response.

The abundances of features in microbiome data often differ by orders of magnitude. As far as we know this has not been explicitly accounted for in the current literature. In the Bayesian lasso [5] separate scale parameters can have a hierarchical prior placed on them rather than this component being marginalised over which results in the Laplace prior. In the regularisation case, the choice of hyperprior defines how the parameters are shrunk to zero. This model is easily extended to the adaptive lasso [21] by positing independent exponential priors on each scale parameter, and then augmenting each tuning parameter with additional hyperpriors.

Typically, model selection is performed using Markov chain Monte Carlo (MCMC) methods. Various stochastic search based methods have been used to explore the model space in a computationally efficient manner ([9, 22, 23]). Despite this body of work, MCMC can still be considered too slow in practice for sufficiently large scale problems. Variational inference is an alternative technique which uses optimisation to achieve computational savings by approximating the marginal posterior densities. Its success in machine learning problems has led to concerted efforts in the literature to encourage its use by statisticians ([24, 25]). The speed of variational inference gives it an advantage, particular for exploratory regression, where a very large model is fitted to gain an understanding of the data and identify a subset of the microbiome which can be explored in more detail.

Approximate solutions arise in variational inference by restricting the family of densities which can be used as a proxy for the exact conditional density. Typically, the mean field variational family is used where independence is assumed across the factors. Thus by specifying conjugate priors, approximate marginal posteriors are members of the exponential family [26]. However, many models of interest such as logistic regression and non conjugate topic models, do not enjoy the properties required to exploit this algorithm. Using variational inference in these settings require algorithms to be adjusted to for the specific model requirement. A variety of strategies have been explored including alternative bounds ([27, 28]), numerical quadrature [29] and Monte Carlo approximation [30].

We propose a Bayesian hierarchical linear log-contrast model for compositional data which is estimated by mean field Monte Carlo co-ordinate ascent variational inference. We use the alr transformation within a log-contrast model which removes the need to specify a reference category. Sparse variable selection is performed through novel priors within a hierarchical prior framework which account for the constrained parameter space associated with the compositional covariates and the different orders of magnitude in the taxon abundances. As our constrained priors are not conjugate, Monte Carlo expectations are used to approximate intractable integrals. These expectations are obtained via a reversible jump Monte Carlo Markov chain (RJMCMC) [31], which is guided by the data through univariate approximations of the intractable variational posterior probability of inclusion. We exploit the nested nature of variational inference by proposing parameters from approximated variational densities via auxiliary parameters. Model averaging over all the explored models can be performed and shrunk estimates of the regression coefficient (by the model uncertainty) are available. The approach accommodates high dimensional microbial data and offers the potential to be scaled up for models with multiple responses.

We compare the performance of the proposed modelling approach with the lasso, the log-contrast lasso [14], two-stage log-ratio lasso [16] and selbal [32] on simulated data. Our method is then applied to a subset of the “Know Your Heart” cross-sectional study of cardiovascular disease [33] in order to examine the association of the gut microbiome with body mass index (BMI). The study was conducted in two Russian cities Novosibirsk and Arkhangelsk, enrolling 4542 men and women aged between 35 and 69 years recruited from the general population. A health check questionnaire was completed, providing information on smoking, weight and levels of alcohol consumption. We analyse the microbiome of 515 subjects from the Arkhangelsk region at the phylum and genus level, as the 16 S rRNA sequencing of faecal samples was only performed for these participants, alongside age and health covariates.

## Methods

### Microbiome model

The microbiome data begins as raw counts for each taxon. Any zeros are replaced by a small pseudo-count (typically 0.5), before each row is standardised to sum to 1. The sample space of a vector of components is a simplex for each data point, where the rows of each vector make up the design matrix ${\varvec{Q}}_{n \times d}$. The set of compositional explanatory variables can be transformed onto the unconstrained sample space ${\mathbb {R}}^{d-1}$ using the alr transformation

1 $$\begin{aligned} alr({\varvec{q}}_i) = \left[ \log \big (\frac{q_{i1}}{q_{id}}\big ), \log \big (\frac{q_{i2}}{q_{id}}\big ),..., \log \big (\frac{q_{id-1}}{q_{id}}\big ) \right] , \end{aligned}$$

where ${\varvec{q}}_i$ is the *i*th row of ${\varvec{Q}}$ and the ratios have been arbitrarily chosen to involve the division of each of the first $d-1$ components by the final component. The log linear model, with the alr transformed variables as proposed by [13], can be expressed as

2 $$\begin{aligned} y_i = \alpha {\varvec{1}}_n + alr({\varvec{q}}_i)\tilde{{\varvec{\theta }}} + \epsilon _i \end{aligned}$$

where $\tilde{{\varvec{\theta }}} = (\theta _1,...,\theta _{d-1})^T$ is the corresponding $(d - 1)$ vector of regression coefficients and $\epsilon _i$ is independent noise distributed as $N(0, \sigma ^2)$. Although convenient, the interpretation of the model depends on the arbitrary choice of the reference category. If we expand the dot product $alr({\varvec{q}}_i) \cdot \tilde{{\varvec{\theta }}}$ and set

3 $$\begin{aligned} \theta _d = -\sum _j^{d-1} {\tilde{\theta }}_j, \end{aligned}$$

the log contrast model can be conveniently expressed in matrix form [14] as

4 $$\begin{aligned} \textbf{y} = \alpha {\varvec{1}}_n + {\varvec{Z}}{\varvec{\theta }} + {\varvec{\epsilon }} \quad \text {subject to } \sum _{j=1}^d \theta _j = 0 \end{aligned}$$

where ${\varvec{Z}}=(\log {\varvec{q}}_1,..., \log {\varvec{q}}_d)$ is the $n \times d$ compositional design matrix and ${\varvec{\theta }} = (\theta _1,...,\theta _d)^T$ is a d-vector of regression coefficients constrained to the affine hyperplane.

This likelihood is used by [18] who specify a *d* dimensional multivariate normal distribution on $\theta$ within a “spike-and-slab” prior,

5 $$\begin{aligned} {\varvec{\theta }}|\sigma ^2, \psi ,\textbf{V} \sim N_{d} (\textbf{0}, \sigma ^2 \psi \textbf{V}), \qquad \textbf{V} = \textbf{I}_d - \frac{c^2}{1 + c^2 d} {\mathbb {J}}_d \end{aligned}$$

where ${\mathbb {J}}_d$ is a matrix of ones and $\textbf{V}$ is the generalised transformation matrix which incorporates the tuning parameter *c* to constrain the ${\varvec{\theta }}$ parameter space and takes the form in (5) for the alr transformation. This approach allows the probability distribution to remain in the *d* dimensional space as $\textbf{V}$ is a matrix of full rank, facilitating conjugate updates, as the sum to zero constraint is not imposed exactly.

Interest often lies in assessing the association of unconstrained data, in the form of categorical or continuous covariates against the response, alongside the microbiome. Two additional design matrices are added to the likelihood, ${\varvec{X}}$ which comprises the scaled continuous covariates and ${\varvec{W}}$ which contains the dummy variables for the $g = 1,...,G$ categorical variables coded to indicate the $m_g$ levels with respect to the intercept. The likelihood for our model is thus expressed as

6 $$\begin{aligned} \textbf{y} = \alpha {\varvec{1}}_n + {\varvec{X}}{\varvec{\beta }} + {\varvec{W}}{\varvec{\zeta }} + {\varvec{Z}}{\varvec{\theta }} + {\varvec{\epsilon }} \quad \text {subject to } \sum _{j=1}^d \theta _j = 0. \end{aligned}$$

### Compositional priors

The linear constraint on the unconstrained vector can be expressed in matrix form as

7 $$\begin{aligned} \textbf{T}= (\textbf{I}_d - (1 / d) {\mathbb {J}}_d) \end{aligned}$$

where $\textbf{T}$ is an idempotent matrix of rank $d-1$. If we originally parametrise $\theta _j \sim N(\mu _j, \psi _j)$, where the large differences in the order of magnitude of each row of the ${\varvec{Z}}$ design matrix are accounted for by allowing each parameter $\theta _j$ to have a separate variance parameter $\psi _j$, then the constrained random variables associated with the compositional explanatory variables are from a singular multivariate normal distribution

8 $$\begin{aligned} {\varvec{\theta }}|{\varvec{\mu }},{\varvec{\psi }} \sim SMVN_d(\textbf{T} {\varvec{\mu }},\textbf{T}\text {diag}({\varvec{\psi }})\textbf{T}^T ) \end{aligned}$$

with ${\varvec{\psi }}$ a vector of scale parameters. This prior respects the sum to zero constraint imposed by the reparametrisation of the likelihood in (6). The distribution is degenerate, the transformation matrix $\textbf{T}$ means the covariance matrix is singular, and will assign 0 values to all sets in the *d* dimensional space. [18] treat the constraint as a tuning parameter, restricting the values that ${\varvec{\theta }}$ can take whilst still remaining in the *d* dimensional space so that the marginal posterior can be obtained in closed form. Our approach imposes the constraint exactly. The singular multivariate normal prior for the compositional data can be considered to be at the unobtainable limit of *c* in the alr transformation approach (5), when the tuning parameter creates a singular matrix where the standard normal prior is no longer appropriate.

We augment the prior on ${\varvec{\theta }}$ with dependent latent indicator variables from a product of Bernoulli distributions which have been truncated to account for the alr transformation which prevents the selection of a single taxon into the model

9 $$\begin{aligned} p({\varvec{\xi }}|\kappa ) \propto \prod _{j=1} \kappa ^ {\xi _{j}} (1- \kappa ) ^{1-\xi _{j}} \textrm{I}\Big [\sum _j\xi _{j} \ne 1 \Big ], \end{aligned}$$

where $\textrm{I}$ is the indicator function. This truncation is particularly important in the presence of sparsity. The full singular multivariate normal spike-and-slab prior for $p({\varvec{\theta }}|{\varvec{\xi }}) = p({\varvec{\theta }}_{\xi }|{\varvec{\xi }})p({\varvec{\theta }}_{{\bar{\xi }}}|{\varvec{\xi }})$, where ${\varvec{\theta }}_\xi$ and ${\varvec{\theta }}_{{\bar{\xi }}}$ are subvectors of ${\varvec{\theta }}$ such that

10 $$\begin{aligned} p({\varvec{\theta }}_\xi | \Sigma , {\varvec{\xi }})&= \frac{1}{(\text {det}^*(2\pi \Sigma ^+_{\xi }))^{(-1/2)}}\exp \left( -\frac{1}{2}{\varvec{\theta }}_{\xi } \Sigma ^+_{\xi }{\varvec{\theta }}_{\xi }\right) \quad \text {and} \quad p({\varvec{\theta }}_{{\bar{\xi }}} =0|{\varvec{\xi }}) = 1, \end{aligned}$$

$\Sigma ^+_\xi$ denotes the Moore-Penrose pseudo inverse of the matrix $\textbf{T}_\xi D({\varvec{\psi }}_\xi )\textbf{T}_\xi$ defined by $A^+= VS^+U^T$ if $A=USV^T$ is the singular value decomposition of A and $S^+$ is the diagonal matrix which has the same entries as *S* and where $S^+_ii=1/S_{ii}$ for the nonzero diagonal entries. The pseudo-determinant $\text {det}^*$ is defined as the product of the nonzero eigenvalues of the matrix and ${\varvec{\xi }}$ is a vector of zeros and ones. The ${\varvec{\theta }}_\xi$ parameters are dependent (the covariance for unit scale is equal to the fraction $- 1/d_\xi$ and for the case of $d_\xi =2$ the correlation is 1). This prior implies a univariate spike-and-slab on the diagonal of the covariance matrix in (10),

11 $$\begin{aligned} p({\varvec{\psi }}|{\varvec{\xi }}) = \prod _{j=1}^d \left[ \frac{b_{\psi }^{a_{\psi }}}{\Gamma (a_{\psi })} (\psi _j)^{-a_{\psi }-1} \exp \{-b_{\psi } \psi ^{-1}_j\}\right] ^{\xi _j} \delta _0(\psi _j)^{1-\xi _j} \quad {\psi _j}>0 ~\forall ~j. \end{aligned}$$

A beta distribution is placed on the sparsity parameter $\kappa$ and the hyperparameter $b_\psi$ is given a gamma prior. This approach can be interpreted as replacing the continuous mixing density in the Bayesian lasso, which can have either hierarchical structure [21] or be marginalised over [5], with a discrete mixture. This set of explicit variable selection priors on the compositional data ensures that the marginal posterior of variable $\xi _j$ represents the inclusion of the *j*th taxon in the model.

### Priors

The choice of the remaining prior distributions is partly down to convenience. The prior distributions and likelihood are semi-conjugate pairs which means the optimal form for the mean field variational density is in the same exponential family form.

We employ a variable selection spike-and-slab prior [34] for $\beta _s$ associated with the continuous variables in the design matrix ${\varvec{X}}$, where each *s* parameter is independent. The spike is a point mass at 0 (Dirac distribution) with probability $1-p(\gamma _s) = 1-\omega$ and the slab is a zero centred Gaussian with variance *w* which requires the variables to be standardised. The binary latent indicator variable $\gamma _s$ represents the inclusion of the *s*th covariate in the model.

In the case of the categorical data matrix, we are interested in selecting the group of variables associated with the response into the model, rather than a particular level. Each factor variable (or group) $g = 1,..,G$ has $j =1,...,m_g, m_{g+1}$ levels which are coded as dummy variables in ${\varvec{W}}$ with reference to the intercept. Motivated by the Bayesian group lasso [35] who introduce binary indicators to perform selection both between and within the groups levels, we employ a variable selection spike-and-slab prior on the vector ${\varvec{\zeta }}_g$ with dimension $m_g$. The spike is a point mass at 0 (Dirac distribution) with probability $1-p(\chi _g) = 1-\varrho$ and the slab is a zero centred Gaussian with variance *v*. The binary latent indicator variable $\chi _g$ represents the inclusion of the *g*th categorical variable into the model. In the case where there factors have just 2 levels, the prior reduces to the same form as its unrestricted continuous counterpart, with a different scale parameter.

Hierarchical priors are also included to fully incorporate the uncertainty surrounding these parameters. The probability that a given covariate in the design matrices of ${\varvec{X}}$ and ${\varvec{W}}$ affects the response is modelled by the parameters $\omega$ and $\varrho$, with beta priors. Inverse gamma distributions with gamma (shape and scale) hyperpriors on their respective scales are placed on the prior variance parameters *w* and *v*.

### Variational inference

We employ coordinate ascent variational inference (CAVI) [36] as our estimation procedure, rather than relying entirely on MCMC which often requires substantial computing resources when the dimensionality of the problem is large. We use structured *mean field variational family*, where dependencies between parameters are explicitly incorporated within blocks and independence is retained across the blocks ([37–40]). Each latent variable is still governed by a distinct factor in the variational density. An example of an approximating posterior block which captures the natural dependency between the latent indicator variable $\gamma _j$ and the corresponding regression coefficient $\beta _j$ directly associated with the design matrix ${\varvec{X}}$ is

12 $$\begin{aligned} q(\beta _j, \gamma _j) = q(\beta _j|\gamma _j)q(\gamma _j). \end{aligned}$$

This leads to a natural type of approximation for hierarchical Bayesian models, where the hierarchical structure of the prior often suggests a good hierarchical structure for the posterior approximation. The full structured mean field approximation distribution $q({\varvec{\vartheta }})$, where ${\varvec{\vartheta }}$ represents all of the latent variables in the model, is defined in the Additional file 1: Sect. 1. The full DAG of the Monte Carlo coordinate ascent variational inference (CAVI-MC) model is in Additional file 1: Fig. S2.

### Unconstrained updates

The variational inference updates are available analytically for all unconstrained parameters and hyperparameters in the model. Derivations are given in the Additional file 1: Sect. 1. The updates involve a combination of univariate and multivariate calculations. The regression parameters directly associated with the ${\varvec{X}}$ and ${\varvec{W}}$ design matrices have joint updates in the same spike-and-slab form as their priors. The conjugate update for $q(\beta _{s}, \gamma _{s})$ is

$$\begin{aligned} q(\beta _{s}|\gamma _{s},\textbf{y})&= {\mathcal {N}}(\mu _{\beta _s},\sigma ^2_{\beta _s})^{\gamma _s}\delta _0(\beta _{s})^{1-\gamma _s} \qquad q(\gamma _{s}|\textbf{y}) = Bern ((\gamma _{s})^{(1)}) \nonumber  \end{aligned}$$

with free parameters

$$\begin{aligned} \sigma ^2_{\beta _s} =&\left( \Vert X_s\Vert ^2 (\sigma ^{-2})^{(1)} + (w^{-1})^{(1)} \right) ^{-1},\\ \mu _{\beta _s} =&(\sigma ^{-2})^{(1)} \sigma ^2_{\beta _s} X_s^T \bigg ( \textbf{y} - (\alpha )^{(1)}{\varvec{1}}_n - \sum _{k \ne s} X_k (\beta _k)^{(1)} + \\&- \sum _g{\varvec{W}}_g({\varvec{\zeta }}_g)^{(1)} - {\varvec{Z}}( {\varvec{\theta }}_\xi )^{(1)} \bigg ) \end{aligned}$$

and

$$\begin{aligned} (\gamma _{s})^{(1)} =&\bigg [ 1 + \exp \bigg \{ (\log (1-\omega ))^{(1)} - (\log \omega )^{(1)} + \\&-\frac{1}{2}\left( (\log w^{-1})^{(1)} -\mu _{\beta _s}^2\sigma ^{-2}_{\beta _s}-\log (\sigma ^2_{\beta _s})\bigg ) \bigg \} \right] ^{-1}, \end{aligned}$$

where $(\cdot )^{(1)}$ denotes the *q* expectation. The conjugate update for $q({\varvec{\zeta }}_g, \chi _g)$ is

13 $$\begin{aligned} q({\varvec{\zeta }}_{g}|\chi _{g}, \textbf{y}) = {\mathcal {N}}_{m_g}({\varvec{\mu }}_{\zeta _g},\Sigma _{\zeta _g})^{\chi _{g}}\delta _0({\varvec{\zeta }}_{g})^{1 - \chi _{g}}\qquad q(\chi _{g}|y) = Bern((\chi _{g})^{(1)}), \end{aligned}$$

where the free parameters for ${\varvec{\zeta }}_g$ are updated by the multivariate extension of the previous univariate update,

$$\begin{aligned} \Sigma _{\zeta _g} =&\left[ (\sigma ^{-2})^{(1)} {\varvec{W}}_g^T{\varvec{W}}_g + (v^{-1})^{(1)} \right] ^{-1},\\ {\varvec{\mu }}_{\zeta _g} =&(\sigma ^{-2})^{(1)} \Sigma _{\zeta _g} {\varvec{W}}_g^T \bigg (\textbf{y} - (\alpha )^{(1)}{\varvec{1}}_n - \sum _sX_s(\beta _s)^{(1)} + \\&- \sum _{k \ne g} {\varvec{W}}_k ({\varvec{\zeta }}_k)^{(1)} - {\varvec{Z}}({\varvec{\theta }})^{(1)} \bigg ), \\ (\chi _{g})^{(1)} =&\bigg [ 1 + \exp \bigg \{ (\log (1-\varrho ))^{(1)} - (\log \varrho )^{(1)} + \\&-\frac{m_g}{2}(\log v^{-1})^{(1)} -\frac{1}{2} {\varvec{\mu }}_{\zeta _g}^T \Sigma _{\zeta _g}^{-1}{\varvec{\mu }}_{\zeta _g} - \frac{1}{2}\log (\text {det}(\Sigma _{\zeta _g})) \bigg \} \bigg ]^{-1} . \end{aligned}$$

The marginal expectation of ${\varvec{\zeta }}_{g}$ and $\beta _s$ is the mean of the conditional density when the parameter is included in the model, shrunk by the probability of being included in the model. The nested *q* density update for each free parameter(s) is the expectation of the log joint distribution with respect to all the other factors. Thus, any update involving a marginal expectation from a parameter with a spike and slab prior involves a form of regularisation.

The selection of the spike-and-slab priors for $\beta _s$, ${\varvec{\zeta }}_g$ and ${\varvec{\theta }}$ with sparsity inducing hyperparameters for variable selection, shrinks the parameters estimates in the variational updates rather then performing explicit variable selection as in MCMC. These estimates are a useful proxy for the final model effects, but as opposed to a model with regularisation priors, the expectation of the model indicator parameters gives us the probability of a covariate being associated with the response. In the case of ${\varvec{\zeta }}_g$, which is associated with the *g*th categorical covariate, the parameterisation has a convenient interpretation. Each element in the vector is free to vary but all elements are shrunk by the same value. Thus the expectation $(\chi _{g})^{(1)}$ is the probability of the categorical covariate (rather than the individual levels) being included in the model.

### CAVI-MC

The conditional vector update $q({\varvec{\theta }}|{\varvec{\psi }},{\varvec{\xi }})$ is available analytically and takes the form

14 $$\begin{aligned} q({\varvec{\theta }}_\xi |{\varvec{\xi }}, \textbf{y})&= SMVN_{d_\xi }(\textbf{T}_\xi {\varvec{\mu }}_{\theta _{\xi }},\textbf{T}_\xi \Sigma _{\theta _{\xi }} \textbf{T}_\xi ),\quad q({\varvec{\theta }}_{{\bar{\xi }}}|{\varvec{\xi }},\textbf{y}) = \delta _0({\varvec{\theta }}_{{\bar{\xi }}}), \end{aligned}$$

where $\delta _0$ is the Dirac distribution on the subvector ${\varvec{\theta }}_{{\bar{\xi }}}$ with updates

15 $$\begin{aligned} {\varvec{\mu }}_{\theta _{\xi }} =&~ \Sigma _{\theta _{\xi }}(\sigma ^{-2})^{(1)}{\varvec{Z}}_{\xi }^T\left( \textbf{y}-(\alpha )^{(1)}{\varvec{1}}_n - \sum _sX_s(\beta _{s})^{(1)} -\sum _g {\varvec{W}}_g ({\varvec{\zeta }}_g)^{(1)}\right)\end{aligned}$$

16 $$\begin{aligned}\Sigma _{\theta _{\xi }} =&~ \left( (\textbf{T}_\xi D({\varvec{\psi }}_\xi )\textbf{T}_\xi )^{+} + (\sigma ^{-2})^{(1)}{\varvec{Z}}_{\xi }^T{\varvec{Z}}_{\xi }\right) ^{-1} \end{aligned}$$

The truncated Bernoulli prior distributions for ${\varvec{\xi }}$ and unique scale parameter $\psi _j$ for each element in ${\varvec{\theta }}$, prevents a conjugate posterior update for the joint block $q({\varvec{\theta }},{\varvec{\psi }},{\varvec{\xi }})$. All other updates are available analytically.

The difficult to compute joint $q({\varvec{\theta }}, {\varvec{\psi }}, {\varvec{\xi }})$ update is performed by inserting a Monte Carlo step within the mean field variational inference approach. We take advantage of the structure of the target density $p({\varvec{\vartheta }},\textbf{y}) \equiv f({\varvec{\vartheta }})$ (the data $\textbf{y}$ is omitted for notational purposes as its fixed) which has the form

17 $$\begin{aligned} f({\varvec{\vartheta }}) = h({\varvec{\vartheta }})\exp (\langle {\varvec{\eta }},T({\varvec{\vartheta }})\rangle - A({\varvec{\eta }}) ), \quad {\varvec{\vartheta }} \in S_p \end{aligned}$$

for *r*-dimensional constant vector ${\varvec{\eta }}$, vector function $T({\varvec{\vartheta }})$ and relevant scalar functions $h > 0$. In our case this admits the factorisation for all $j \notin {\mathcal {J}}$

$$\begin{aligned} h({\varvec{\vartheta }}) = h_{q(\vartheta _j)}(\vartheta _j) h_{q({\varvec{\vartheta }}_{-j})}({\varvec{\vartheta }}_{-j}), \qquad T_l({\varvec{\vartheta }}) = T_{l,j}(\vartheta _j)T_{l,-j}({\varvec{\vartheta }}_{-j}), \quad 1 \le l \le r, \end{aligned}$$

where ${\mathcal {J}}$ is the set of all analytically available updates. This allows us to avoid generating and storing the samples from the approximating densities which would involve considerable computational cost, by using the *q* marginal expectations in the Monte Carlo estimate for $q({\varvec{\theta }}|{\varvec{\psi }},{\varvec{\xi }})$. [30] show that, under regularity conditions, an CAVI-MC recursion will get arbitrarily close to a maximiser of the evidence lower bound with any given high probability.

The MCMC approach involves two move types, within-model moves where the samples are generated from a Metropolis-Hastings sampler and between-model moves which are sampled from a RJMCMC. The samplers involve using some form of the joint approximating posterior $q({\varvec{\theta }},{\varvec{\psi }}, {\varvec{\xi }}|{\varvec{y}}) \propto ~ q({\varvec{\theta }}|{\varvec{\psi }}, {\varvec{\xi }},{\varvec{y}})q({\varvec{\xi }},{\varvec{\psi }}|{\varvec{y}})$ which is simplified as $q({\varvec{\theta }}|{\varvec{\psi }}, {\varvec{\xi }},{\varvec{y}})$ has the conjugate spike-and-slab form (14).

Randomly choose either a between-model move which consists of sequentially updating ${\varvec{\xi }},{\varvec{\psi }}|{\varvec{\xi }}$ and ${\varvec{\theta }}|{\varvec{\psi }}$, ${\varvec{\xi }}$ or a within-model move where ${\varvec{\xi }}$ is not updated. This naturally leads to questions regarding the proposals for ${\varvec{\psi }}$ which has a constrained support and ${\varvec{\xi }}$ which has the potential to be a very large binary space.

#### Between-model RJMCMC - approximating $q({\varvec{\xi }}, {\varvec{\psi }}|{\varvec{y}})$ to $p({\varvec{\xi }}|{\varvec{\vartheta }})$ for the proposal distribution $j_m({\varvec{\xi }},{\varvec{\xi }}')$

The choice of priors for the parameters associated with microbiome features, the indicator vector ${\varvec{\xi }}$ and set of scale parameters ${\varvec{\psi }}_\xi$, prevents a conjugate update for $q({\varvec{\theta }}, {\varvec{\psi }}, {\varvec{\xi }})$. An MCMC step is introduced to sample from the intractable *q* approximating posterior. To search the binary space we use a RJMCMC where the proposal for $\psi _j$ conditional on $\xi _j = 1$ is from the *q* approximating density of the auxiliary parameter $\Omega _j$

18 $$\begin{aligned} \pi (\psi _j|\xi _j = 1) = IG_q(a_{\Delta _j}^*, b_{\Delta _j}^*), \end{aligned}$$

where the calculation of the free parameters $a_{\Delta _j}^*$ and $b_{\Delta _j}^*$ is explained in the next section. ${\varvec{\theta }}$ is generated directly from the singular multivariate normal target distribution (14).

There is considerable research in sampling high-dimensional binary vectors. [41] propose a general model for the proposal which combines local moves with global ones by changing blocks of variables. They find that the acceptance rates for Metropolis-Hastings samplers that include, exclude or swap a single variable improves. [22] extend their model with adaptive parameters which change during the mixing of the MCMC. Motivated by incorporating information from data into the proposal parameters, we use the variational inference posterior distribution $q({\varvec{\xi }},{\varvec{\psi }}|\textbf{y})$ which is only available up to a constant of proportionality

19 $$\begin{aligned} q\left( {{\varvec{\xi }},{\varvec{\psi }}\left| {\textbf{y}} \right.} \right) \propto ~ & \exp \left( {\frac{1}{2}\left( {{\varvec{\mu }}_{{\theta _{{(\xi ,\psi )}} }}^{T} {\textbf{T}}_{\xi } \left( {{\textbf{T}}_{\xi }^{T} \Sigma _{{\theta _{{(\xi ,\psi )}} }} {\textbf{T}}_{\xi } } \right)^{ + } {\textbf{T}}_{\xi } {\varvec{\mu }}_{{\theta _{{(\xi ,\psi )}} }} } \right) + } \right. \\ & + \frac{1}{2}\log \left( {\det ^{*} ({\textbf{T}}_{\xi } \Sigma _{{\theta _{{(\xi ,\psi )}} }} {\textbf{T}}_{\xi } )} \right) + \sum\limits_{j} {\xi _{j} } (\log \kappa )^{{(1)}} - \frac{1}{2}\log \left( {\det ^{*} \left( {{\textbf{T}}_{\xi } D({\varvec{\psi }}_{\xi } ){\textbf{T}}_{\xi } } \right)} \right) + \\ & + \sum\limits_{j} {(1 - \xi _{j} )(\log (1 - \kappa ))^{{(1)}} } - (a_{\psi } + 1)\sum\limits_{j} {\xi _{j} \log (\psi _{j} ) - b_{\psi } \sum\limits_{j} {\xi _{j} } \psi _{j}^{{ - 1}} } + \\ & \left. { + (a_{\psi } \log (b_{\psi } ) - \log (\Gamma (a_{\psi } ))\sum\limits_{j} {\xi _{j} } } \right), \\ \end{aligned}$$

to obtain a univariate approximation relative to the *j*th element to guide the RJMCMC. These normalised probabilities are used to obtain our proposal probabilities in a birth-death and swap sampling scheme. Similar to adaptive parameters in MCMC, these selection probabilities are updated at each iteration of the CAVI.

The pseudo determinant in (19) is approximated by removing the constraints $\textbf{T}_{\xi }$ and taking the MCMC expectation conditional on $\xi _{j} = 1$. So for the *j*th element the approximation is

20 $$\begin{aligned} \log (\text {det}^*(\textbf{T}_{\xi } D({\varvec{\psi }}_{\xi })\textbf{T}_{\xi } )) \approx \{\log (\psi _{j})\}^{\{1\}}_{{0}\!\!\!/}, \end{aligned}$$

where the curly brackets $\{\}$ denote an MCMC expectation and ${0}\!\!\!/$ defines an expectation over all non-zero values. A similar approach can be used to approximate the determinant containing $\Sigma _{\theta _\xi }$

$$\begin{aligned} \log (\text {det}^*(\textbf{T}_{\xi }\Sigma _{\theta _{\xi }}\textbf{T}_{\xi })) \approx \log ({\bar{\sigma }}_{\theta _j}^2), \end{aligned}$$

where ${\bar{\sigma }}_{\theta_j}^2$ is the non-zero variance average over the MCMC iterations, obtained by extracting the diagonal from $\Sigma _{\theta _{(\xi ,\psi )}}$ at each iteration. If the *j*th term has not been included in the model the term is approximated by

21 $$\begin{aligned} \log (\text {det}^*(\textbf{T}_{\xi }\Sigma _{\theta _{\xi }}\textbf{T}_{\xi })) \approx \log \left( \left[ \Vert Z_j\Vert ^2 (\sigma ^{-2})^{(1)} \right] ^{-1}\right) . \end{aligned}$$

After approximating $\Sigma _{\theta _{\xi }}$ to a scalar for each *j*th element the matrix dot product reduces to

22 $$\begin{aligned} {\varvec{\mu }}_{\theta _{\xi }}^T\textbf{T}_{\xi }( \textbf{T}_{\xi }^T\Sigma _{\theta _{\xi }}\textbf{T}_{\xi })^+ \textbf{T}_{\xi } {\varvec{\mu }}_{\theta _{\xi }} \approx {\bar{\sigma }}_{\theta _j}^2 \Big ( \sum _j(1-1/d_{\xi })\mu _{\theta _{\xi _{j}}}^2 -2\sum _{j<j'}(\mu _{\theta _{{\xi }_{j'}}}\mu _{\theta _{{\xi }_{j}}}/d_{\xi }) \Big ). \end{aligned}$$

To account for the cross product terms which contains the elements of ${\varvec{\xi }}$ not equal to *j* and the associated ${\varvec{\mu }}_{\theta }$ terms, a combination of conditional expectations and marginal expectations which shrink the values in proportion to its probability of being zero, is used. As $\xi _{j}$ can not be separated from the sum in the numerator $d_{\xi }$, two approximations of the matrix dot product are used conditional on the expectation from the previous chain.

Defining the expectations with respect to the parameter currently being updated from the previous MCMC by a curly bracket as:

- $\{\mu _{\theta _{j}}\}^{\{1\}}_{{0}\!\!\!/}$: Conditional expectation $\xi _{j}=1$. Weighted average of the nonzero terms from previous chain,
- $\{\mu _{\theta _{j}}\}^{\{1\}}$: Expectation wrt *q* from the previous chain,
- $\{d_{\xi }\}^{\{1\}}$: Expectation wrt *q* from the previous chain,

the approximation of the dot product $(\textbf{T}_{\xi } {\varvec{\mu }}_{\theta _{\xi }})^T \textbf{T}_{\xi } {\varvec{\mu }}_{\theta _{\xi }}$ for $\{d_{\xi }\}^{\{1\}} >2$ is thus

$$\begin{aligned} {\bar{\sigma }}_{\theta_j}^{-2}\Bigg ( \sum _j(1-1/\{d_{\xi }\}^{\{1\}})\xi _{j}(\{\mu _{\theta _{j}}\}^{\{1\}}_{{0}\!\!\!/})^2 -\frac{2}{\{d_{\xi }\}^{\{1\}}} \sum _{j<j'}\xi _{j}\{\mu _{\theta _{{\xi }_{j}}}\}_{{0}\!\!\!/}^{\{1\}}\{\mu _{\theta _{{\xi }_{j'}}}\}^{\{1\}}\Bigg ) \end{aligned}$$

and

$$\begin{aligned} {\bar{\sigma }}_{\theta_j}^{-2} \left( \sum _j\xi _{j}(\{\mu _{\theta _{j}}\}^{\{1\}}_{{0}\!\!\!/})^2 \right) \qquad \text {for } \{d_{\xi }\}^{\{1\}} <2. \end{aligned}$$

Although $\{ d_{\xi } \in {\mathbb {N}}_0 | d_{\xi } \le d, d_{\xi } \ne 1 \}$, the support of the MCMC expectation $\{d_{\xi }\}^{\{1\}}$ is the positive real line so we threshold on 2. When $\{d_{\xi }\}^{\{1\}}>2$ the probabilities used in the proposal distribution for the RJMCMC, derived from approximating Equation (19) and normalising is

23 $$\begin{aligned} \tilde{p}\left( {\xi _{j} = 1\left| \vartheta \right.} \right) \equiv & \left[ {\exp \left\{ {(\log (1 - \kappa ))^{{(1)}} - \frac{1}{{2\bar{\sigma }_{{\theta ,j}}^{2} }}\left( {(1 - 1/\{ d_{\xi } \} ^{{\{ 1\} }} )(\{ \mu _{{\theta _{j} }} \} _{\phi }^{{\{ 1\} }} )^{2} + } \right.} \right.} \right. \\ & \;\left. { - \frac{2}{{\{ d_{\xi } \} ^{{\{ 1\} }} }}\{ \mu _{{\theta _{{\xi _{j} }} }} \} _{\phi }^{{\{ 1\} }} \sum\limits_{{j^{\prime} \ne j}} {\{ \mu _{{\theta _{{\xi _{{j^{\prime}}} }} }} \} ^{{\{ 1\} }} } } \right) - \frac{1}{2}\log (\bar{\sigma }_{{\theta ,j}}^{2} ) + \frac{1}{2}(\log \psi _{j} )_{\phi }^{{\{ 1\} }} - (\log \kappa )^{{(1)}} \\ & \;\left. {\left. { + \left( {\log \Gamma (a_{\psi } ) - a_{\psi } \log b_{\psi } } \right) + (a_{\psi } + 1)(\log \psi _{j} )_{\phi }^{{\{ 1\} }} + b_{\psi } (\psi _{j}^{{ - 1}} )_{\phi }^{{\{ 1\} }} } \right\} + 1} \right]^{{ - 1}} \\ \end{aligned}$$

which contains the variational expectations and an MCMC conditional expectation from the previous iterations. This is then used to propose the various move types in the RJMCMC.

#### Pseudo updates for MCMC proposals

A conjugate update for the parameters associated with the microbiome features $q({\varvec{\theta }}, {\varvec{\psi }}, {\varvec{\xi }})$ is prevented by the choice of priors for the indicator vector ${\varvec{\xi }}$ and set of scale parameters ${\varvec{\psi }}_\xi$. Samples from the intractable *q* approximating posterior are simulated from an MCMC step instead. The move types in the RJMCMC for ${\varvec{\xi }}$ use an element-wise approximation of the joint $q({\varvec{\xi }})$ density (23). For the proposal distribution of ${\varvec{\psi }}$, we use the model likelihood and an unconstrained approximation to the constrained priors. In order to do this we define auxiliary parameters (upper case Greek letters) which are unconstrained versions of the constrained parameters. We derive pseudo variational updates from an unconstrained model with a simpler prior parametrisation, then use the *q* approximating distribution of the relevant auxiliary parameter as our proposal for ${\varvec{\psi }}$. We can think of the auxiliary parameters as introducing an alternative directed acyclic graph (DAG) which is updated first, helping us to approximate the model in order to guide the MCMC step (depicted in the Additional file 1: Fig. S1). These updates are refined by the full variational inference updates which account for the constraint at each iteration. The parameter $\kappa$ and the hyperparameters $a_{\Delta }, b_{\Delta }$ which are set to $a_{\psi }, b_{\psi }$ provide a link back to the constrained model.

The series of pseudo variational updates are determined from a simple prior parametrisation where the parameters associated with the compositional covariates are not constrained to sum to 0. This unconstrained model has the following prior parametrisation

$$\begin{aligned} p( \Omega _{j} | \Delta _{j},\Upsilon _{j})&= N(\Omega _{j}|0,\Delta _{j})^{\Upsilon _{j}}\delta _0(\Omega _{j})^{1 - \Upsilon _{j}}, \\ p(\Delta _{j}|\Upsilon _{j})&= IG(\Delta _{j}|a_{\Delta }, b_{\Delta })^{\Upsilon _{j}} \delta _0(\Delta _{j})^{1-\Upsilon _{j}}, \\p(\Upsilon _{j})&= Bern(\Upsilon _{j}|\kappa ), \\ \end{aligned}$$

where ${\varvec{\Omega }}$ are the unconstrained version of the ${\varvec{\theta }}$ parameters, ${\varvec{\Delta }}$ are the variance parameters for ${\varvec{\Omega }}$ which are both dependent on the model selection parameters ${\varvec{\Upsilon }}$. The prior for the model selection parameter $\Upsilon _{j}$ is a simple Bernoulli distribution. The remaining priors and likelihood take the form defined in the initial prior parametrisation. The introduction of independence across each univariate $(\Omega _{j}, \Delta _{j}, \Upsilon _{j})$ block, (where the data is being treated as unconstrained) ensures the *q* expectations are all available in closed form (derived in the Additional file 1: Sect. 1).

Despite the similarities of the prior parametrisation to (13), the addition of a separate scale parameter $\Delta _{j}$ for $\Omega _{j}$ prevents a joint conjugate update on the $(\Omega _{j}, \Delta _{j}, \Upsilon _{j})$ block. Instead we update $q(\Omega _j, \Upsilon _j)$ (for $j=1,...,d$) before updating $q(\Delta _j|\Upsilon _j)$. Both require expectations conditional on $\Upsilon _j$ as well as the typical marginal expectations. The full $q(\Omega _{j}, \Upsilon _{j})$ update is

24 $$\begin{aligned}&q(\Omega _j, \Upsilon _j) \propto N(\Omega _j |\mu _{\Omega _j},\sigma ^2_{\Omega _j})^{\Upsilon _j} \delta _0(\Omega _j)^{1-\Upsilon _j}. \end{aligned}$$

25 $$\begin{gathered} \left\{ {\exp \left( {\frac{1}{2}\log \sigma _{{\Omega _{j} }}^{2} + (\log \kappa )^{{(1)}} - \frac{{\mathbb{E}_{q} (\log \Delta _{j} |\Upsilon _{j} )}}{2} + \frac{{\mu _{{\Omega ,j}}^{2} \sigma _{{\Omega ,j}}^{{ - 2}} }}{2} + a_{\Delta } \log (b_{\Delta } ) + } \right.} \right. \hfill \\ \left. {\left. { - \log (\Gamma (a_{\Delta } )) - (a_{\Delta } + 1)\mathbb{E}_{q} (\log \Delta _{j} |\Upsilon _{j} ) - b_{\Delta } \mathbb{E}_{q} [\Delta _{j}^{{ - 1}} |\Upsilon _{j} ]} \right)} \right\}^{{\Upsilon _{j} }} \hfill \\ \left\{ {(1 - \kappa )^{{(1)}} + \delta _{0} (\Delta _{j} )} \right\}^{{1 - \Upsilon _{j} }} \hfill \\ \end{gathered}$$

The binary form of the pseudo update for $\Omega _j$ and $\Upsilon _j$ enables us to determine the values for the conditional expectations. In (24) we have under *q*, where we condition on the value of $\Upsilon _j$

26 $$\begin{aligned} q(\Omega _{j}|\Upsilon _{j} = 1,\textbf{y})&= {\mathcal {N}}(\mu _{\Omega ,j},\sigma ^2_{\Omega ,j}) \quad q(\Omega _{j}|\Upsilon _{j}=0,\textbf{y}) = \delta _0(\Omega _{j}), \end{aligned}$$

which allows us to set the expectations in the normal variance update as ${\mathbb {E}}_q[\Delta _j^{-1}|\Upsilon _j = 1 ]$

27 $$\begin{aligned} \sigma ^2_{\Omega ,j}&= \left( \Vert Z_j\Vert ^2 (\sigma ^{-2})^{(1)} + {\mathbb {E}}_q[\Delta _j^{-1}|\Upsilon _j = 1 ] \right) ^{-1} \end{aligned}$$

28 $$\begin{aligned} \mu _{\Omega ,j}&= \sigma ^2_{\Omega ,j} Z_j^T \Bigg \{(\sigma ^{-2})^{(1)}\Bigg ( \textbf{y} - \sum _{k \ne j} Z_k (\Omega _k)^{(1)} - \sum _s X_s (\beta _s)^{(1)} \Bigg ) \Bigg \}. \end{aligned}$$

The conditional expectation prevents us averaging over $\Upsilon _j$ which shrinks the marginal expectation, creating an update which has the same form as (13). Using the form of (25) to determine the conditional expectation and normalising gives the probability of inclusion

$$\begin{aligned} (\Upsilon _{j})^{(1)} =&\Bigg [ \exp \Bigg \{\frac{\log (\sigma ^{-2}_{\Omega ,s})}{2}+ (\log (1-\kappa ))^{(1)} - (\log \kappa )^{(1)} + \log \Gamma (a_\Delta ) + \nonumber \\&+\frac{{\mathbb {E}}_q(\log \Delta _j|\Upsilon _j = 1) }{2} -\frac{1}{2}\mu _{\Omega ,j}^2\sigma ^{-2}_{\Omega ,j} + (a_\Delta + 1){\mathbb {E}}_q(\log \Delta _j|\Upsilon _j = 1) + \nonumber \\&+b_\Delta {\mathbb {E}}_q[\Delta _j^{-1}|\Upsilon _j = 1] - a_\Delta \log (b_\Delta ) \Bigg \} + 1 \Bigg ]^{-1}. \nonumber \end{aligned}$$

The univariate approximation of $q({\varvec{\xi }},{\varvec{\psi }}|{\varvec{y}})$ (23) can be interpreted as a refinement of $(\Upsilon _{j})^{(1)}$ using MCMC expectations and information on all elements of ${\varvec{\xi }}$ to partially account for the constraint in the probability of inclusion.

The spike-and-slab form of the pseudo update for $q(\Delta _{j}|\Upsilon _{j})$ allows us to again back out the conditioning in the conditional expectation of ${\mathbb {E}}_q[\Omega ^2_j|\Upsilon _j]$ in $b^*_{\Delta _j}$.

$$\begin{aligned} q(\Delta _{j}|\Upsilon _{j},\textbf{y}) = IG\left( \Delta _j \bigg |\frac{1}{2} + a_{\psi },\frac{(\sigma ^2_{\Omega ,j} + \mu ^2_{\Omega ,j})}{2} + b_{\psi }\right) ^{\Upsilon _{j}} \delta _0(\Delta _{j})^{1-\Upsilon _{j}}. \nonumber \end{aligned}$$

As the update $\Delta _{j}$ is conditional on $\Upsilon _j$, the free parameters in the proposal distributions are not a function of shrunken estimates. The $q(\Delta _j|\Upsilon _j, \textbf{y})$ auxiliary approximating density is then used to propose scale parameters with the appropriate support, which are informed by the data, for ${\varvec{\psi }}_\xi$ in the MCMC move.

#### Algorithm

CAVI is performed by iterating through the analytical variational updates, maximising the evidence lower bound (ELBO) with respect to each coordinate direction whilst fixing the other coordinate values. For the $q({\varvec{\theta }},{\varvec{\psi }},{\varvec{\xi }})$ block an MCMC is implemented to obtain Monte Carlo estimates of the intractable marginal expectations of the approximating densities. The proposal probabilities for the sampling scheme are a function of the data and the free parameters, and are updated at each iteration of the CAVI.

For each run we compute the ELBO (derived in Additional file 1: Sect. 1), with the updated free parameters, until this converges to the local optimum. The ELBO is no longer monotonically increasing because of the Monte Carlo variability, but we are able to declare convergence when the random fluctuations are small around a fixed point. The implementation of the overall approach is described in Algorithm 1, with the MCMC move detailed in 2.

It is computationally inefficient to start with a large number of iterations *m*, when the current variational distribution can be far from the maximiser. The software allows the user to specify a smaller number of iterations to begin with before increasing the number of iterations as the algorithm becomes more stable, improving the accuracy of the Monte Carlo estimates.
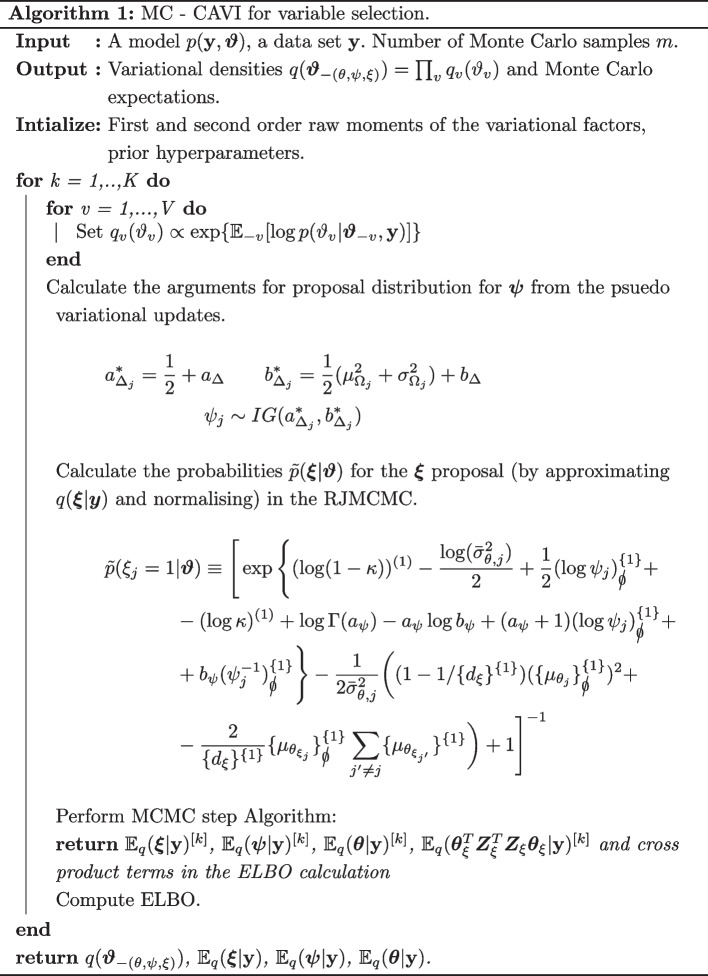



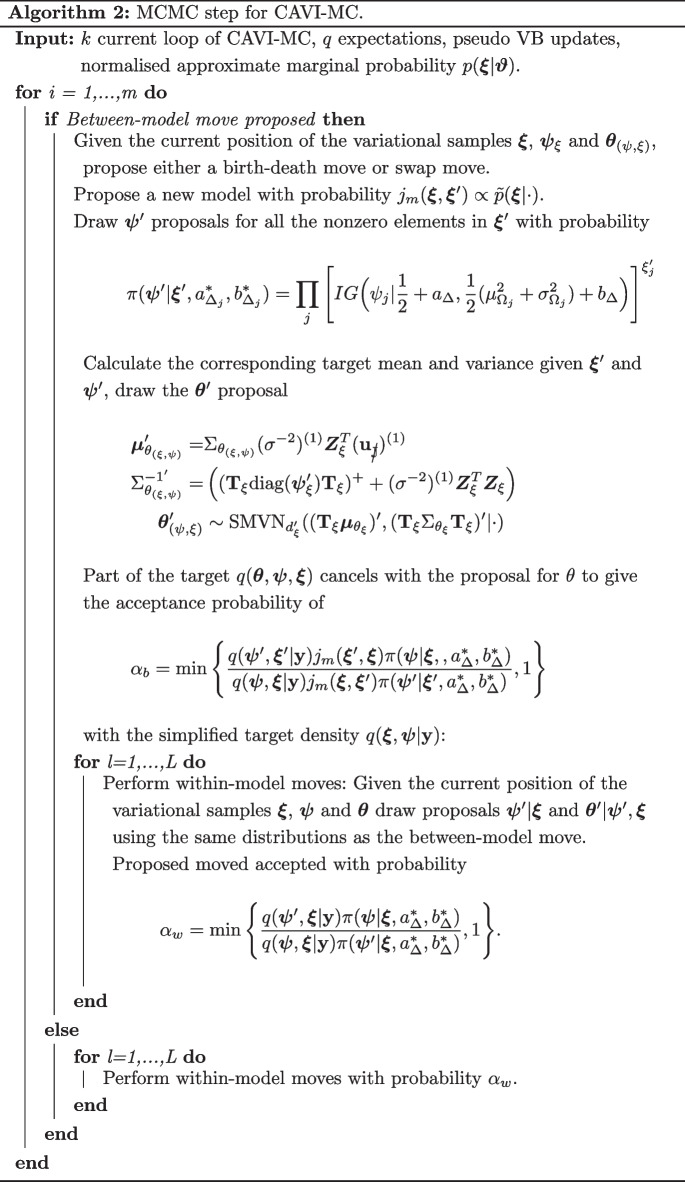


## Simulation study

We validate the performance of our variational inference model, focusing on the compositional element, in a simulation experiment against four frequentist variable selection approaches with software freely available; $L_1$ lasso [42], log-contrast lasso [14], two-stage log-ratio lasso [16] and selbal [32]. All except the vanilla lasso explicitly account for the compositional nature of the design matrix within the feature selection.

We simulate data from an additive log-ratio model. An $n \times d$ data matrix $\textbf{O} = (o_{ij})$ is drawn from a multivariate normal distribution $N_p({\varvec{\mu }}_o, \Sigma _o)$, and then the compositional covariate matrix $\textbf{Q}= (q_{ij})$ is obtained via the transformation $q_{ij} = \frac{\exp (\tau o_{ij})}{\sum _{k=1}^d \exp (\tau o_{ik})}$. The covariates thus follow a logistic normal distribution [43]. To account for the differences in the order of magnitudes of the components so common in microbiome data, we fix $\tau = 2$ and let $\mu _{oj} = \log (d \times 0.5)$ for j = 1,..., 5 and $\mu _{oj}=0$ otherwise. Thus we have 5 of the compositional (or microbiome) features with a larger order of magnitude. We vary the number of compositional features *d* from 45 to 200 for $n=100$, to ensure a setting where the features out number the observations. As the correlations between the abundances of features in the microbiome can vary quite considerably according to the taxonomy class, we choose three settings for $\Sigma _o$: $\Sigma _o = \textbf{I},~(\rho ^{|i-j|} )$ with $\rho = 0.2$ or $0.4$.

We select 6 compositional features to be associated with the response, 3 of which have a larger order of magnitude, via a *d* dimensional ${\varvec{\theta }}$ vector with non-zero elements ${\varvec{\theta }}_{\xi } = (1, -1.5, 0.5, -1, 1.5, -0.5)$. The signal to noise ratio (SNR) is defined as $\text {SNR} = \text {Mean}(|{\varvec{\theta }}_\xi |) / \sigma$. To generate the data with the various choices of SNR, this expression is solved for $\sigma$ and 100 simulations are generated.

Rearranging the simulated data model as we have only 3 unique non-zero values of ${\varvec{\theta }}$

29 $$\begin{aligned} y_i = 1 \log (q_{i1}/ q_{i6}) + 1.5 \log (q_{i2}/ q_{i7}) + 0.5 \log (q_{i3}/ q_{i8})+ \epsilon_i \text{ for } i=1,..., n, \end{aligned}$$

we obtain the all-pairs log-ratio model with a 0 intercept, as in [16].

The log-contrast lasso of [14] is a $L_1$ penalization lasso on the log transformed variables with the additional constraint that the sum of coefficients is zero. This is fitted using the glmnet.constr in R, by augmenting the data with an additional data point with all features equal to 1 and a response value of zero. By assigning this value a large weight the resulting parameter estimates will approximately sum to zero. A two-stage log-ratio lasso procedure by [16] builds on the [14] model by adding an additional forward selection step which effectively prunes the model for a sparser solution, whilst maintaining the parameter constraint. The selbal [32] is a balance selection algorithm which starts with a search of the two taxa whose balance, a log-contrast where the coefficients of the linear function sum to zero, is most closely associated with the response. Once the first two-variable balance is selected, the algorithm performs a forward selection process to add further variables to the model. For all of the comparison methods, prediction and cross-validation is performed over a grid of values to determine model selection and tuning parameter estimation.

As the focus of the simulation study is on the compositional element, the parameters associated with the unrestricted design matrix are omitted from the Bayesian structure in the CAVI-MC. Vague priors are placed on the hyperparameters for the CAVI-MC model (highlighted with red in the DAG (Additional file 1: Fig. S2)). Standard variable selection in high-dimensional data with spike-and-slab priors in a Bayesian framework is well understood [44]. The sparsity of the compositional features is controlled by the choice of $a_\kappa$ and $b_\kappa$ on the Beta hyperprior on $\kappa$. We fix their choice by specifying a prior average number of covariates, $d^*$, expected to be included in the model, setting

30 $$\begin{aligned} a_\kappa = 1 \qquad b_\kappa = \frac{(d - d^*)}{d}. \end{aligned}$$

We perform the simulations with $d^*$ equal to 6 or 12 and report the average, reflecting uncertainty in $d^*$.

Since the optimisation problem for maximizing the ELBO is non-convex, the approach can be sensitive to initialization of the variational parameters. For each simulation, the variational parameters are initialized via random samples from the associated prior distribution. 25 variational inference iterations are performed (although the algorithm typically converges after approximately 6 iterations) for each run. The initial number of between-model MCMC iterations is set to 5000, before 10,000 iterations are performed after the 5th set of variational inference updates.

To assess the performance of the approaches we use metrics which evaluate the ability to select the correct variables and estimate the appropriate effects. The prediction error (PE), defined as $\text {PE} = \frac{1}{n_{test}}(\textbf{y}_{test}-\textbf{Z}_{test}\hat{{\varvec{\theta }}}_{train})^T(\textbf{y}_{test}-\textbf{Z}_{test}\hat{{\varvec{\theta }}}_{train})$, is computed using an independent test sample of size 5. We compute the $l_2$ loss $||\hat{{\varvec{\theta }}}- {\varvec{\theta }}||^2$ and bias squared to assess the accuracy of the coefficient estimates. To asses the accuracy of the variable selection, the true positive rate (TPR or sensitivity) and false positive rate (FPR or 1 - specificity) is reported, where positives and negatives in the context of the frequentist approaches refer to non-zero and zero coefficients respectively. For each of these metrics, the respective standard deviation across datasets is included. Variable selection for the CAVI-MC is performed by thresholding the marginal approximate posterior distribution ${\mathbb {E}}[q(\xi _j|y)]$ at 0.5. The approximate posterior mean is used for the parameter estimate of the Bayesian model.

**Fig. 1:**
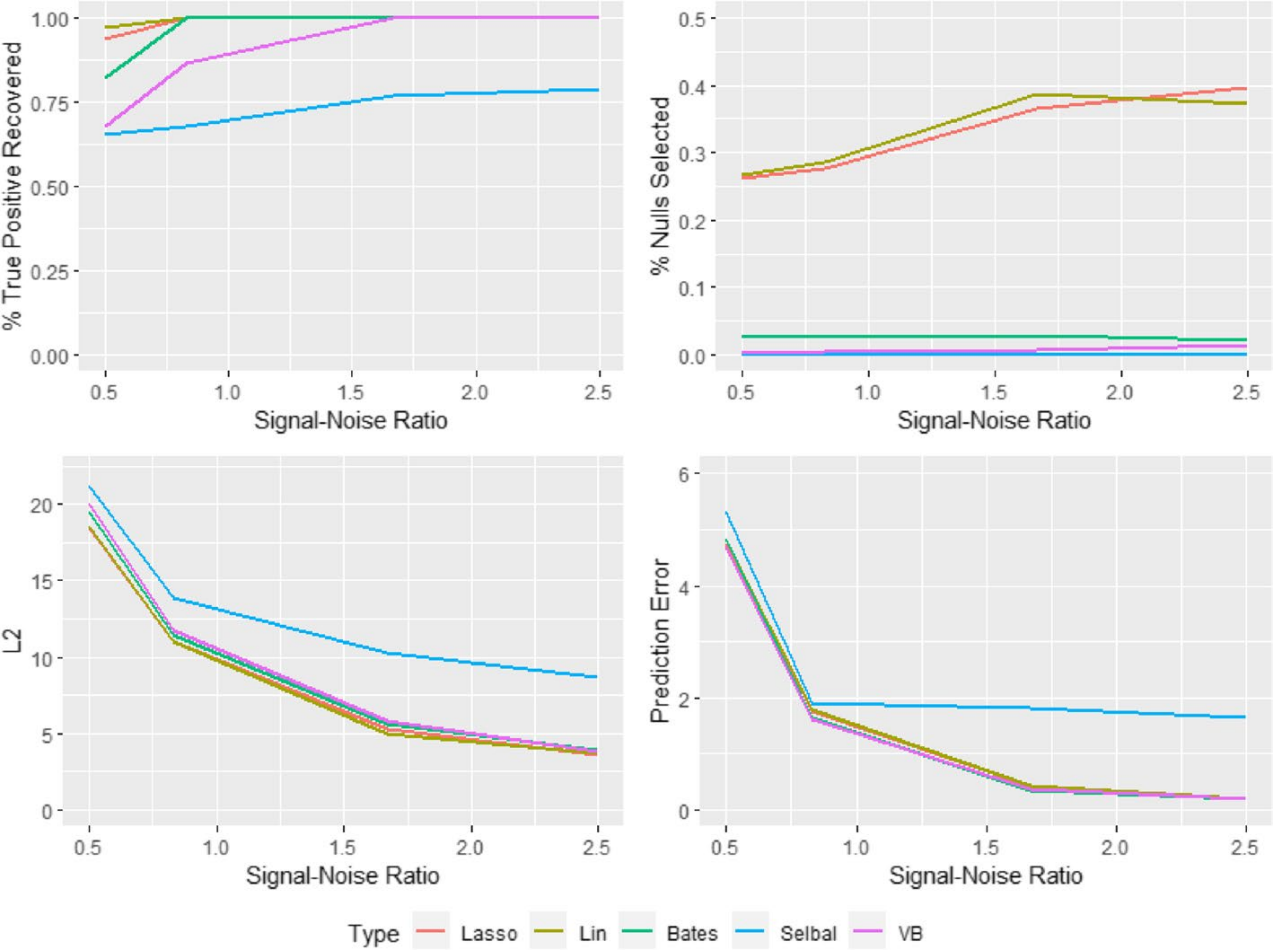
Results of the simulation study for $d=45$ and $\rho =0$. The “% true positive recovered” reports the proportion of times that the true parameters are selected in the model. The “% nulls selected” graph shows the average fraction of null variables selected in the model

**Fig. 2:**
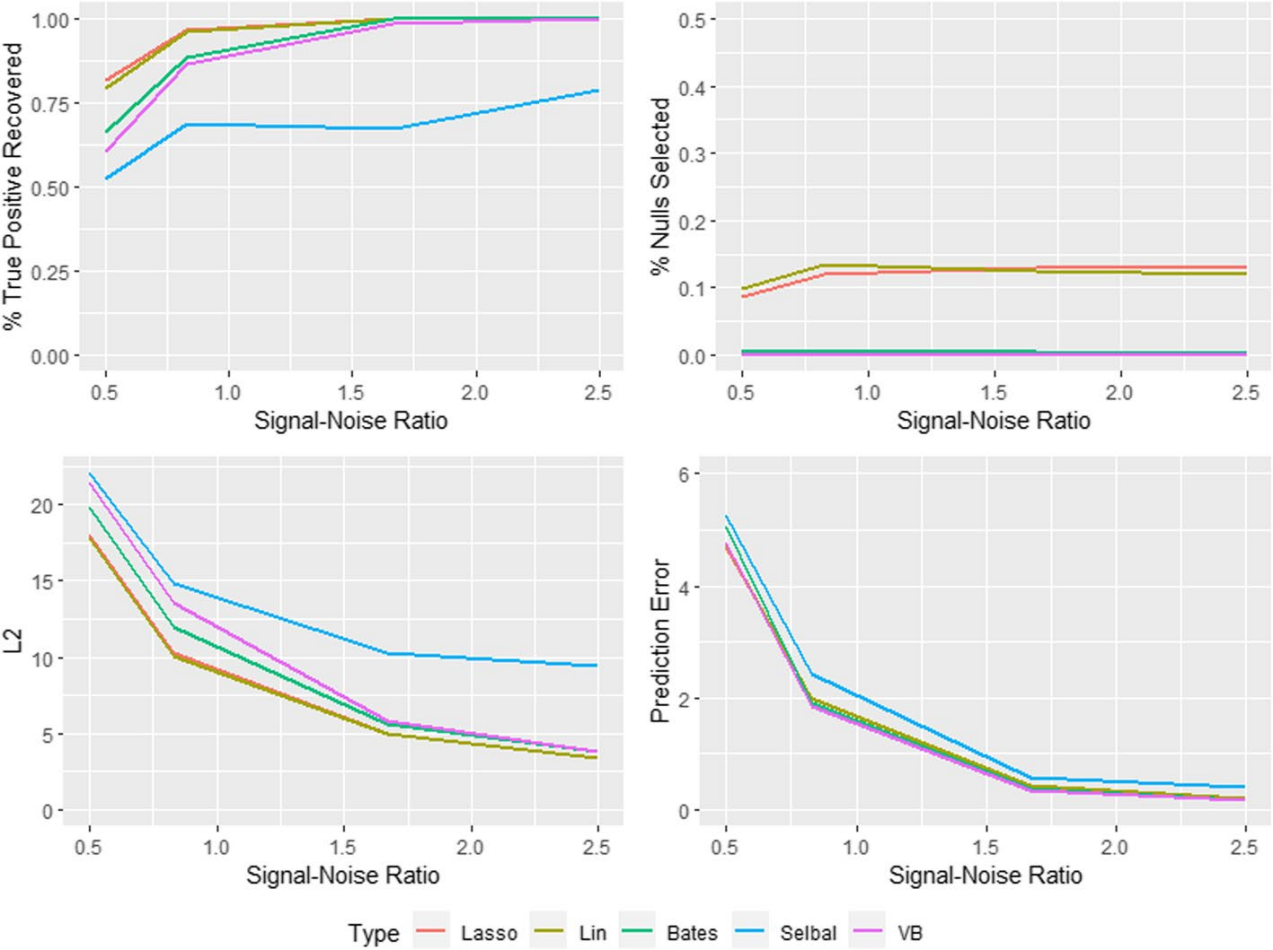
Results of the simulation study for $d=200$ and $\rho =0$. The “% true positive recovered” reports the proportion of times that the true parameters are selected in the model. The “% nulls selected” graph shows the average fraction of null variables selected in the model

The vanilla lasso and the log-contrast lasso consistently detect the true parameters for low SNRs, but this comes at a considerable cost of a high false positive rate. This failure to capture the sparsity of the true model is a function of the the number of compositional covariates and the correlation between the compositional covariates. The results for $\rho =0$ and $d=45$ and $d=200$ are plotted in Figs. 1 and 2. When *d* is 45 and $\rho =0.4$ these two approaches can incorrectly select over a third of the covariates. This is not the case with either the selbal, log-ratio lasso or the CAVI-MC. These methods control for false positives whilst still maintaining a high probability of identifying the correct features.

The proposed CAVI-MC Bayesian method out performs the constrained lasso and selbal with respect to FPR, and prediction error (Tables 1, 2, 3, 4, 5, 6, 7 and 8). The performance of the CAVI-MC is very similar to the log-ratio lasso for moderate and strong SNRs. The CAVI-MC requires slightly more signal to detect the true parameters, but consistently outperforms the log-ratio lasso in controlling for false positives. The Bayesian approach has the additional benefit of a posterior distribution for each of the associated compositional parameters $\theta _j$ in the model and additional model flexibility. Where as the log-ratio lasso is restricted to models of the form of (29), the CAVI-MC can accurately capture models for any number of unique non-zero values.

**Table 1:** Table of true positive rate, false positive rate, L2 loss and prediction error for the additive-log-ratio model with SNR of 0.5 and *d* of 45

Method	$\rho$	TPR	FPR	L2	PE
Lasso	0	0.937 ± 0.091	0.262 ± 0.136	18.465 ± 1.933	4.741 ± 2.901
Lin	0	0.972 ± 0.067	0.266 ± 0.124	18.572 ± 1.847	4.828 ± 2.928
Bates	0	0.822 ± 0.147	0.029 ± 0.048	19.504 ± 1.803	4.837 ± 3.059
Selbal	0	0.655 ± 0.117	0.002 ± 0.008	21.159 ± 1.541	5.305 ± 3.570
VB	0	0.678 ± 0.111	0.002 ± 0.008	20.072 ± 1.560	4.687 ± 2.948
Lasso	0.2	0.882 ± 0.133	0.271 ± 0.140	18.442 ± 1.996	5.051 ± 2.951
Lin	0.2	0.897 ± 0.125	0.292 ± 0.143	18.526 ± 2.215	4.939 ± 2.866
Bates	0.2	0.767 ± 0.128	0.042 ± 0.057	19.425 ± 1.828	5.023 ± 2.727
Selbal	0.2	0.637 ± 0.114	0.005 ± 0.012	21.259 ± 1.718	4.815 ± 2.800
VB	0.2	0.640 ± 0.141	0.002 ± 0.007	20.156 ± 1.549	4.410 ± 2.475
Lasso	0.4	0.825 ± 0.137	0.294 ± 0.163	18.330 ± 2.118	4.661 ± 3.396
Lin	0.4	0.835 ± 0.139	0.354 ± 0.151	18.322 ± 2.180	4.651 ± 3.205
Bates	0.4	0.692 ± 0.165	0.058 ± 0.079	19.471 ± 2.169	4.583 ± 3.437
Selbal	0.4	0.550 ± 0.168	0.004 ± 0.012	21.134 ± 1.699	4.733 ± 3.852
VB	0.4	0.428 ± 0.211	0.001 ± 0.005	20.835 ± 2.021	4.370 ± 2.948

Bates refers to the two-stage log-ratio lasso and VB refers to the CAVI-MC

**Table 2:** Table of true positive rate, false positive rate, L2 loss and prediction error for the additive-log-ratio model with SNR of 0.5 and *d* of 200

Method	$\rho$	TPR	FPR	L2	PE
Lasso	0	0.820 ± 0.118	0.086 ± 0.056	18.037 ± 3.142	4.673 ± 2.765
Lin	0	0.793 ± 0.130	0.099 ± 0.061	17.830 ± 3.280	4.746 ± 2.753
Bates	0	0.665 ± 0.124	0.007 ± 0.013	19.862 ± 2.434	5.052 ± 3.219
Selbal	0	0.523 ± 0.165	0.001 ± 0.002	21.987 ± 2.050	5.257 ± 3.453
VB	0	0.608 ± 0.138	0.000 ± 0.001	21.392 ± 2.388	4.760 ± 3.139
Lasso	0.2	0.797 ± 0.145	0.097 ± 0.069	18.149 ± 3.537	5.903 ± 3.475
Lin	0.2	0.813 ± 0.136	0.129 ± 0.082	17.236 ± 4.241	6.590 ± 3.841
Bates	0.2	0.662 ± 0.186	0.009 ± 0.015	19.785 ± 2.792	6.229 ± 4.564
Selbal	0.2	0.523 ± 0.162	0.001 ± 0.003	21.988 ± 1.979	6.763 ± 3.814
VB	0.2	0.617 ± 0.141	0.002 ± 0.007	20.739 ± 2.174	6.409 ± 3.599
Lasso	0.4	0.705 ± 0.150	0.112 ± 0.071	17.574 ± 3.755	7.193 ± 4.369
Lin	0.4	0.615 ± 0.203	0.125 ± 0.088	17.864 ± 4.715	8.344 ± 4.456
Bates	0.4	0.545 ± 0.199	0.011 ± 0.015	20.014 ± 2.700	6.401 ± 3.994
Selbal	0.4	0.455 ± 0.162	0.002 ± 0.004	21.533 ± 1.790	6.932 ± 5.012
VB	0.4	0.295 ± 0.180	0.000 ± 0.001	23.043 ± 2.210	9.111 ± 5.407

**Table 3:** Table of true positive rate, false positive rate, L2 loss and prediction error for the additive-log-ratio model with SNR of 0.83 and *d* of 45

Method	$\rho$	TPR	FPR	L2	PE
Lasso	0	1.000 ± 0.000	0.277 ± 0.139	10.979 ± 1.114	1.766 ± 1.191
Lin	0	1.000 ± 0.000	0.287 ± 0.140	10.978 ± 1.182	1.786 ± 1.215
Bates	0	0.985 ± 0.053	0.026 ± 0.041	11.456 ± 1.011	1.639 ± 1.113
Selbal	0	0.680 ± 0.061	0.000 ± 0.000	13.925 ± 1.022	1.904 ± 1.294
VB	0	0.867 ± 0.140	0.004 ± 0.009	11.784 ± 1.135	1.618 ± 1.125
Lasso	0.2	0.993 ± 0.033	0.314 ± 0.140	10.932 ± 1.112	1.597 ± 0.884
Lin	0.2	0.998 ± 0.017	0.329 ± 0.134	10.997 ± 1.042	1.579 ± 0.869
Bates	0.2	0.965 ± 0.080	0.042 ± 0.056	11.478 ± 0.929	1.564 ± 0.929
Selbal	0.2	0.672 ± 0.037	0.000 ± 0.000	14.366 ± 0.903	1.746 ± 0.978
VB	0.2	0.900 ± 0.113	0.002 ± 0.007	11.878 ± 0.962	1.473 ± 0.889
Lasso	0.4	0.965 ± 0.080	0.352 ± 0.149	10.840 ± 1.248	1.692 ± 0.872
Lin	0.4	0.972 ± 0.071	0.426 ± 0.151	10.752 ± 1.280	1.674 ± 0.951
Bates	0.4	0.868 ± 0.145	0.043 ± 0.064	11.701 ± 1.241	1.602 ± 0.966
Selbal	0.4	0.668 ± 0.050	0.000 ± 0.000	13.395 ± 0.958	1.643 ± 0.879
VB	0.4	0.802 ± 0.135	0.001 ± 0.008	12.510 ± 1.064	1.413 ± 0.707

**Table 4:** Table of true positive rate, false positive rate, L2 loss and prediction error for the additive-log-ratio model with SNR of 0.83 and *d* of 200

Method	$\rho$	TPR	FPR	L2	PE
Lasso	0	0.965 ± 0.068	0.120 ± 0.084	10.238 ± 2.330	1.998 ± 1.127
Lin	0	0.962 ± 0.082	0.135 ± 0.089	10.070 ±2.661	2.002 ± 1.114
Bates	0	0.887 ± 0.141	0.004 ± 0.009	11.923 ± 1.318	1.909 ± 1.196
Selbal	0	0.687 ± 0.150	0.000 ± 0.000	14.837 ± 1.199	2.424 ± 1.475
VB	0	0.725 ± 0.104	0.000 ± 0.001	12.706 ± 1.119	1.962 ± 1.061
Lasso	0.2	0.972 ± 0.075	0.134 ± 0.076	9.911 ± 2.239	2.045 ± 1.214
Lin	0.2	0.972 ± 0.071	0.158 ± 0.079	9.580 ± 2.344	2.351 ± 1.443
Bates	0.2	0.925 ± 0.126	0.004 ± 0.009	11.691 ± 1.368	1.806 ± 1.178
Selbal	0.2	0.657 ± 0.074	0.001 ± 0.001	14.494 ± 1.011	2.649 ± 1.624
VB	0.2	0.720 ± 0.170	0.000 ± 0.001	12.947 ± 1.908	2.186 ± 1.496
Lasso	0.4	0.847 ± 0.125	0.142 ± 0.084	10.054 ± 2.316	3.142 ± 1.834
Lin	0.4	0.810 ± 0.121	0.172 ± 0.084	9.630 ± 2.456	3.341 ± 1.861
Bates	0.4	0.798 ± 0.142	0.008 ± 0.015	11.790 ± 1.642	1.860 ± 1.116
Selbal	0.4	0.633 ± 0.085	0.001 ± 0.001	13.986 ± 1.097	2.669 ± 1.896
VB	0.4	0.603 ± 0.139	0.000 ± 0.001	13.611 ± 1.971	2.418 ± 1.797

**Table 5:** Table of true positive rate, false positive rate, L2 loss and prediction error for the additive-log-ratio model with SNR of 1.67 and *d* of 45

Method	$\rho$	TPR	FPR	L2	PE
Lasso	0	1 ± 0.000	0.365 ± 0.179	5.265 ± 0.699	0.432 ± 0.308
Lin	0	1 ± 0.000	0.388 ± 0.164	5.310 ± 0.686	0.434 ± 0.331
Bates	0	1 ± 0	0.029 ± 0.058	5.685 ± 0.580	0.358 ± 0.257
Selbal	0	0.770 ± 0.091	0.000 ± 0.000	9.644 ± 0.762	1.810 ± 0.729
VB	0	1 ± 0	0.008 ± 0.015	5.633 ± 0.492	0.378 ± 0.247
Lasso	0.2	1 ± 0	0.334 ± 0.166	5.389 ± 0.623	0.471 ± 0.289
Lin	0.2	1 ± 0	0.308 ± 0.164	5.321 ± 0.686	0.482 ± 0.295
Bates	0.2	1 ± 0	0.022 ± 0.036	5.805 ± 0.454	0.445 ± 0.275
Selbal	0.2	0.667 ± 0	0 ± 0	10.274 ± 0.549	1.364 ± 0.568
VB	0.2	1 ± 0	0.003 ± 0.008	5.705 ± 0.438	0.416 ± 0.263
Lasso	0.4	1 ± 0	0.378 ±0.141	5.384 ± 0.5875	0.523 ± 0.346
Lin	0.4	1 ± 0	0.423 ± 0.141	5.358 ± 0.598	0.536 ± 0.333
Bates	0.4	1 ± 0	0.019 ± 0.035	5.792 ± 0.427	0.417 ± 0.257
Selbal	0.4	0.667 ± 0.024	0 ± 0	9.365 ± 0.547	0.945 ± 0.581
VB	0.4	1 ± 0	0.008 ± 0.015	5.633 ± 0.492	0.441 ± 0.273

**Table 6:** Table of true positive rate, false positive rate, L2 loss and prediction error for the additive-log-ratio model with SNR of 1.67 and *d* of 200

Method	$\rho$	TPR	FPR	L2	PE
Lasso	0	1 ± 0	0.130 ± 0.077	4.959 ± 1.004	0.436 ± 0.310
Lin	0	1 ± 0	0.124 ± 0.066	5.000 ± 0.988	0.429 ± 0.288
Bates	0	1 ± 0	0.005 ± 0.012	5.664 ± 0.629	0.364 ± 0.234
Selbal	0	0.673 ± 0.400	0.000 ± 0.000	10.296 ± 0.561	0.588 ± 0.351
VB	0	0.985 ± 0.063	0.000 ± 0.001	5.801 ± 0.676	0.354 ± 0.230
Lasso	0.2	1 ± 0	0.146 ± 0.072	4.780 ± 0.920	0.474 ± 0.294
Lin	0.2	1 ± 0	0.165 ± 0.079	4.641 ± 0.981	0.549 ± 0.352
Bates	0.2	1 ± 0	0.005 ± 0.011	5.641 ± 0.558	0.434 ± 0.259
Selbal	0.2	0.667 ± 0.102	0 ± 0	10.379 ± 0.503	1.047 ± 0.544
VB	0.2	1 ± 0	0.001 ± 0.002	6.231 ± 0.438	0.526 ± 0.382
Lasso	0.4	1 ± 0	0.143 ± 0.064	4.912 ± 0.915	0.472 ± 0.273
Lin	0.4	1 ± 0	0.182 ± 0.073	4.607 ± 1.010	0.492 ± 0.317
Bates	0.4	1 ± 0	0.008 ± 0.009	5.541 ± 0.522	0.430 ± 0.285
Selbal	0.4	0.667 ± 0.017	0 ± 0	9.204 ± 0.528	1.138 ± 0.538
VB	0.4	0.978 ± 0.077	0.000 ± 0.001	5.810 ± 0.626	0.397 ± 0.249

**Table 7:** Table of true positive rate, false positive rate, L2 loss and prediction error for the additive-log-ratio model with SNR of 2.5 and *d* of 45

Method	$\rho$	TPR	FPR	L2	PE
Lasso	0	1 ± 0	0.397 ± 0.159	3.647 ± 0.386	0.211 ± 0.125
Lin	0	1 ± 0	0.374 ± 0.144	3.668 ± 0.377	0.214 ± 0.122
Bates	0	1 ± 0	0.022 ± 0.034	3.914 ± 0.303	0.191 ± 0.119
Selbal	0	0.790 ± 0.073	0.000 ± 0.000	8.645 ± 0.562	1.637 ± 0.526
VB	0	1 ± 0	0.015 ± 0.019	3.848 ± 0.278	0.197 ± 0.119
Lasso	0.2	1 ± 0	0.297 ± 0.134	3.723 ± 0.360	0.184 ± 0.107
Lin	0.2	1 ± 0	0.279 ± 0.137	3.817 ± 0.366	0.196 ± 0.113
Bates	0.2	1 ± 0	0.019 ± 0.033	3.957 ± 0.284	0.169 ± 0.102
Selbal	0.2	0.667 ± 0	0 ± 0	9.161 ± 0.404	1.182 ± 0.369
VB	0.2	1 ± 0	0.004 ± 0.010	3.870 ± 0.267	0.159 ± 0.091
Lasso	0.4	1 ± 0	0.366 ± 0.145	3.544 ± 0.371	0.226 ± 0.163
Lin	0.4	1 ± 0	0.429 ± 0.139	3.533 ± 0.393	0.229 ± 0.150
Bates	0.4	1 ± 0	0.019 ± 0.035	3.811 ± 0.316	0.186 ± 0.115
Selbal	0.4	0.667 ± 0	0 ± 0	8.192 ± 0.386	0.707 ± 0.279
VB	0.4	1 ± 0	0.017 ± 0.017	4.104 ± 0.313	0.208 ± 0.126

**Table 8:** Table of true positive rate, false positive rate, L2 loss and prediction error for the additive-log-ratio model with SNR of 2.5 and *d* of 200

Method	$\rho$	TPR	FPR	L2	PE
Lasso	0	1 ± 0	0.130 ± 0.077	3.384 ± 0.637	0.206 ± 0.124
Lin	0	1 ± 0	0.120 ± 0.064	3.412 ± 0.645	0.202 ± 0.127
Bates	0	1 ± 0	0.003 ± 0.007	3.847 ± 0.339	0.178 ± 0.115
Selbal	0	0.667 ± 0.000	0.000 ± 0.000	9.460 ± 0.367	0.396 ± 0.191
VB	0	1 ± 0	0.001 ± 0.002	3.797 ± 0.284	0.178 ± 0.106
Lasso	0.2	1 ± 0	0.150 ± 0.075	3.293 ± 0.662	0.230 ± 0.138
Lin	0.2	1 ± 0	0.172 ± 0.069	3.107 ± 0.657	0.269 ± 0.167
Bates	0.2	1 ± 0	0.003 ± 0.006	3.849 ± 0.302	0.184 ± 0.118
Selbal	0.2	0.667 ± 0.000	0.000 ± 0.000	9.399 ± 0.362	0.791 ± 0.351
VB	0.2	1 ± 0	0.001 ± 0.002	3.667 ± 0.319	0.254 ± 0.156
Lasso	0.4	1 ± 0	0.170 ± 0.080	3.142 ± 0.711	0.230 ± 0.133
Lin	0.4	1 ± 0	0.186 ± 0.067	3.095 ± 0.672	0.226 ± 0.132
Bates	0.4	1 ± 0	0.005 ± 0.006	3.792 ± 0.313	0.180 ± 0.104
Selbal	0.4	0.667 ± 0.000	0.000 ± 0.000	8.019 ± 0.356	0.876 ± 0.316
VB	0.4	1 ± 0	0.000 ± 0.001	3.671 ± 0.295	0.186 ± 0.110

Each of the methods ability to detect the true parameters in the model deteriorate in the presence of large correlation and low SNR (Tables 1 and 2 ). The selbal appears to be the most robust method for larger correlation but clearly struggles to select the correct features even with much higher SNRs. The between-model moves in the CAVI-MC rely on a RJMCMC which is guided by an approximation of the likelihood. When the signal is low and correlation high, this reduces the ability to guide the sampler to the the true parameters within a large binary space. The approximation of $q({\varvec{\xi }},{\varvec{\psi }}|\textbf{y})$ for univariate proposals is slightly less effective at identifying the correct features, compared with the log-contrast lasso approach which identifies the initial variables for the log-ratio lasso.

The $L_2$ loss and squared bias diagnostics (Additional file 1: Tables 9–16) indicate the CAVI-MC estimates the model well, as it typically outperform all but the log-ratio lasso. Given the true model in the simulation study is a log-ratio model (29), the log-ratio lasso benefits from estimating a much smaller number of parameters than the other methods. As the CAVI-MC is more flexible than the log-ratio lasso, the squared bias for this simulation scenario is typically larger, but this comes with the distinct advantage of being able to accurately capture a much large space of models.

**Fig. 3:**
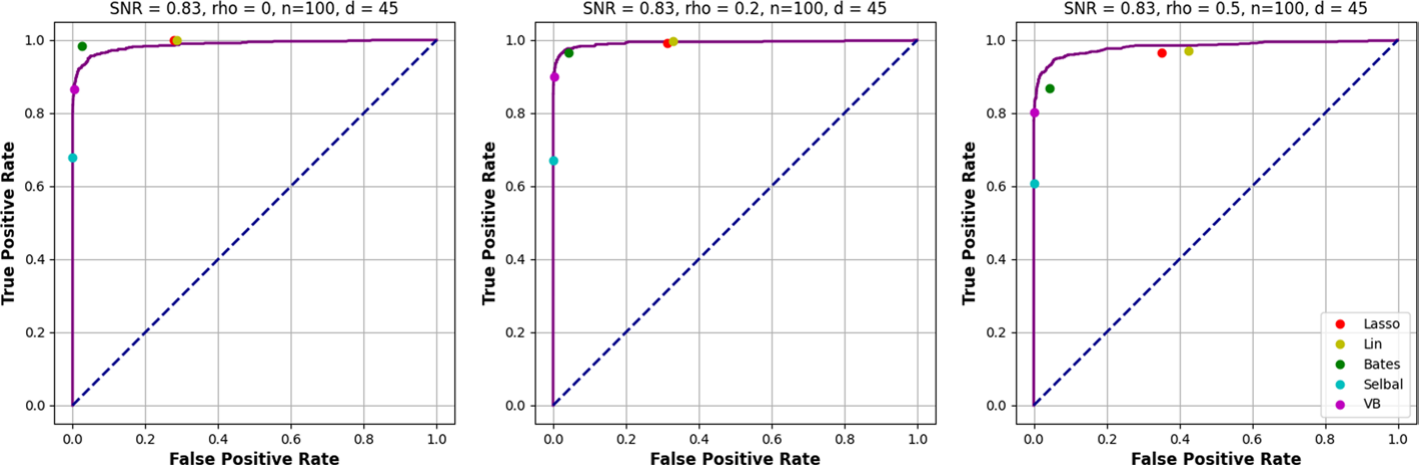
Plot of the ROC curves for the CAVI-MC for a SNR of 0.83 for each value of $\rho$

The performance of the CAVI-MC, from varying the thresholding value for ${\mathbb {E}}[q(\xi _j|\textbf{y})]$ when the SNR of 0.83, is plotted in Fig. 3 where the purple point represents the value for 0.5. Despite the log-ratio lasso having a larger TPRs, the points for $\rho =0.2$ and $\rho =0.4$ fall inside the CAVI-MC ROC curve (further ROC curves are in the Additional file 1: Fig. S3–S5).

## Data

We apply our proposed method to a subset of the main study in Arkhangelsk, containing 515 men and women aged between 35 and 69 years recruited from the general population, from the “Know your Heart” cross-sectional study of cardiovascular disease [33]. As part of the study, participants were asked to volunteer faecal samples for analysis of the gut microbiome. The relative abundances of the microbes were then determined by 16 S rRNA sequencing (using the variable regions V3–V4) followed by taxonomic classification using a Naive Bayes classifier [45]. A baseline questionnaire captured unconstrained covariate information on age, sex and smoking status. Information on alcohol consumption from the questionnaire and biomarker data was used to derive a categorical factor with four levels on alcohol use.

The gut microbiome plays an important role in energy extraction and obesity [46], which we illustrate by regressing body mass index (BMI) against the microbiome at the phylum and genus level alongside the unconstrained covariates. The counts are transformed into relative abundances after adding a small constant of 0.5 to replace the zero counts [47] and then log transformed. BMI is also log transformed and the continuous age covariate is standardised.

Vague priors are placed on the hyperparameters for the CAVI-MC model. Given the previous results from microbiome against BMI analysis, $d^*$ for the hyperprior on $\kappa$ is set to 8. The birth-death or swap move parameter $\phi$ is set to 0.5. Four runs of the CAVI-MC algorithm are performed, each with different initialisation values for the *q* expectations and the ELBO is monitored to confirm convergence. For each run 20 variational inference iterations are performed (although the algorithm typically converges after approximately 6 iterations). The initial number of between-model MCMC iterations is set to 5000, before 10,000 iterations are performed after the 5th set of variational inference updates.

**Fig. 4:**
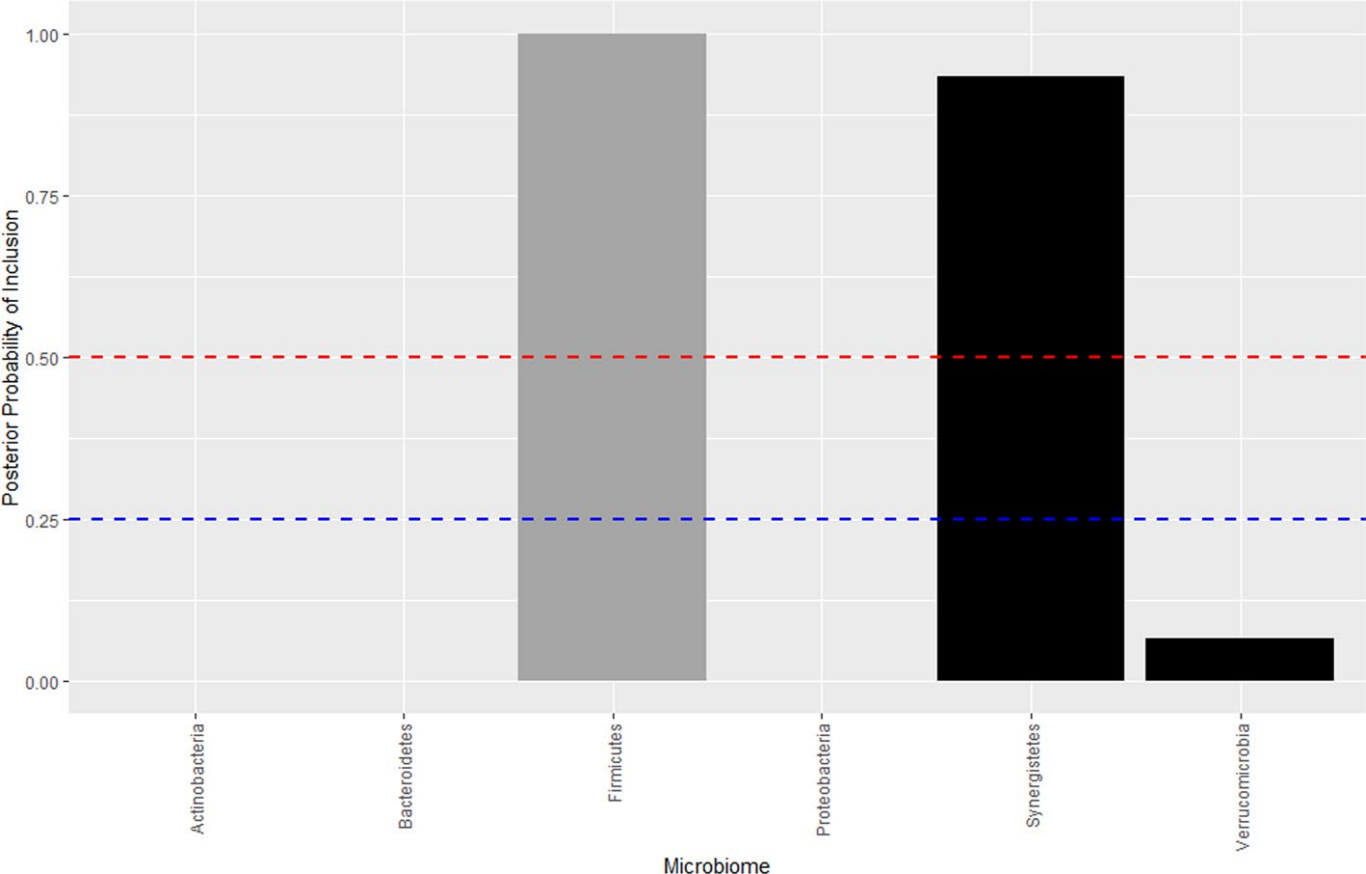
Plot of the marginal expectation of the approximating posterior ${\mathbb {E}}_q[p({\varvec{\xi }}|\textbf{y})]$ at the phylum level. The grey denotes a positive $\theta _j$, black a negative $\theta _j$. The bars above the 0.5 probability of inclusion (red dashed line) are *Firmicutes* and *Synergistetes* respectively

**Fig. 5:**
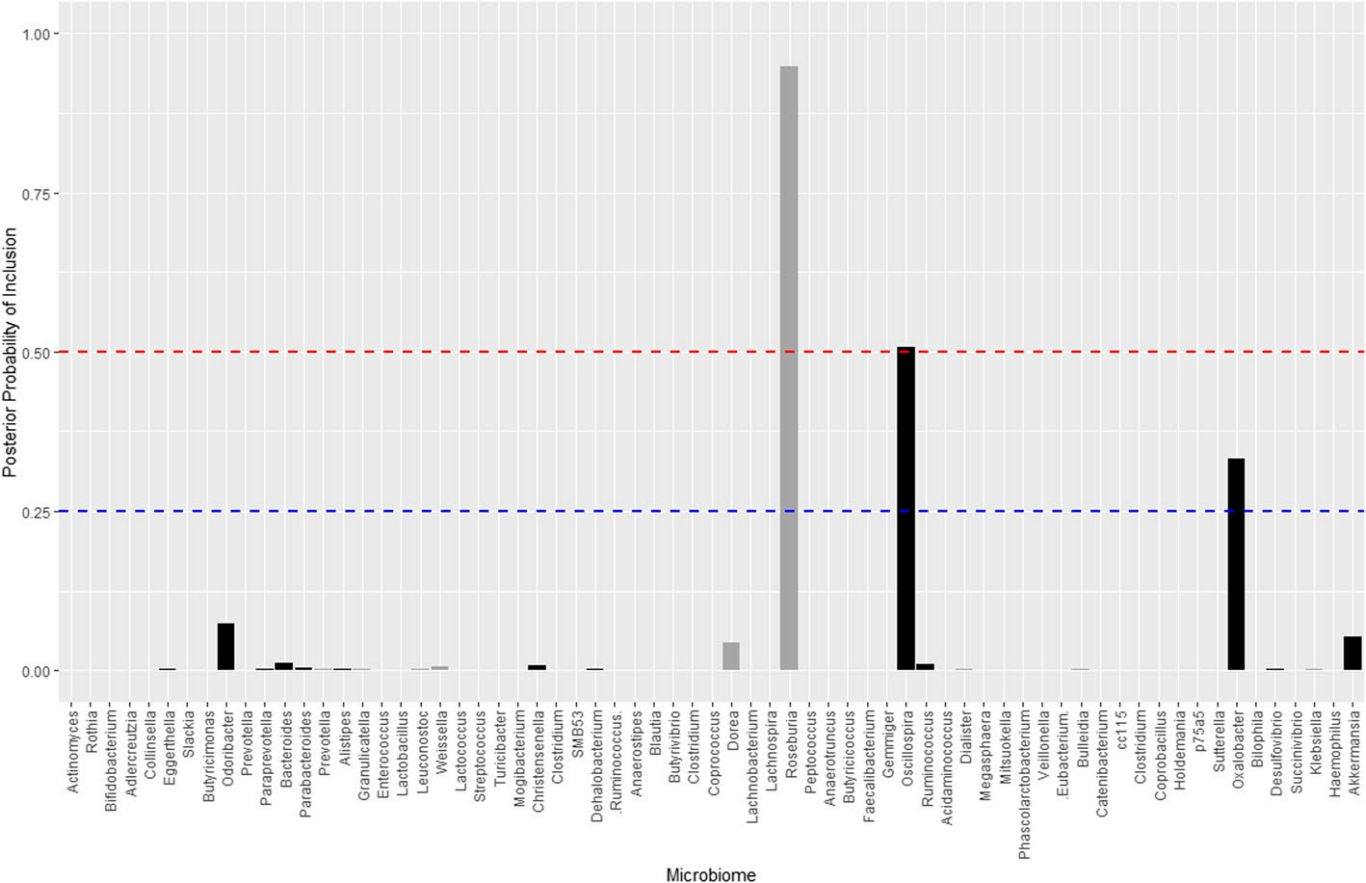
Plot of the marginal expectation of the approximating posterior ${\mathbb {E}}_q[p({\varvec{\xi }}|\textbf{y})]$ at the genus level. The grey denotes a positive $\theta _j$, black a negative $\theta _j$. The bars above 0.25 probability of inclusion (blue dashed line) are *Roseburia*, *Oscillospira* and *Oxalobacter* respectively. The red dashed line at 0.5 probability of inclusion indicate the thresholding value used to determine a significant association

Despite different initial starting point the CAVI-MC converges to the same maximum. Thresholding the marginal expectation of the approximate posterior distributions at 0.5, we find an increase in Firmicutes (which has a −0.8 correlation with Bacteroidetes) and a decrease in Synergistetes is associated with an increase of BMI at the phylum level. At the genus level, BMI is increased by an increase in *Roseburia* and a reduction in *Oscillospira*. The corresponding marginal expectation of the approximating posterior ${\mathbb {E}}[q({\varvec{\xi }}|y)]$, for both the phylum and genus level are plotted in Figs. 4 and 5. We also find BMI to be positively associated with age. The ELBO for each model at each microbiome level indicates an optimum has been reached (Additional file 1: Fig. S6 and S7), with each run finding the same local optimum.

Our findings appear to be consistent with previous studies. The ratio of Firmicutes to Bacteroidetes at the phylum level is considered to be a biomarker for obesity ([48, 49]). Increases in physical training of rats has led to an increase in their levels of Synergistetes [50]. At the genus level [51] identifies *Roseburia* to be positively correlated with obesity in children, and [52] determines *Oscillospira* to be negatively associated with BMI.

## Discussion

Our Bayesian hierarchical linear log-contrast model estimated by structured mean field Monte Carlo co-ordinate variational inference improves Bayesian regression modelling for compositional data. Sparse variable selection is performed through priors which fully account for the constrained parameter space associated with the compositional covariates. We introduce Monte Carlo expectations to approximate integrals which are not available in closed form. These expectations are obtained via RJMCMC with proposal parameters informed by approximating variational densities via auxiliary parameters with pseudo updates. As long as there is sufficient signal to guide the RJMCMC, the approach leads to a high TPR and low FPR in compared with frequentist compositional approaches.

The CAVI-MC suffers when the SNR is low and the correlation is high. Addressing the correlation by adapting the prior parametrisation may help to improve the model in these settings. One approach to address this issue is to use a Markov Random Field prior [53] which imposes a structure on the selection of ${\varvec{\xi }}$. [18] use this prior to incorporate the phylogenetic relationship among the bacterial taxa alongside a model which partially accounts for the constraint on the parameters. Alternatively, to avoid having to pre-define the structure of the taxa, a Dirichlet Process could be used to account for the correlation of the microbiome by clustering the covariates [54] prior to the regression.

At the genus level, despite the CAVI-MCMC identifying associations between the BMI and *Roseburia* and *Oscillospira*, some of the other microbiome features which have been found to be associated with BMI were not detected. *Bifidobacterium* has been found to be negatively associated with BMI in children [55]. This taxon was also found to be associated with BMI in adults, alongside a negative association between BMI and *Methanobrevibacter* [56]. However, associations between BMI and the gut microbiome at the genus level are subject to a high degree of variation across studies [57]. This maybe partly explained by the tools used to construct the microbiome datasets, which can identify quite different results from the same sample [58].

As genetic sequencing becomes more widely available, interest grows in modelling the relationship between the microbiome and a complex set of phenotypes such as blood concentrations of lipids or other metabolites. Bayesian hierarchical models have been introduced for multiple outcomes ([59, 60]), which leverage shared information improving predictor selection. These approaches often use the simplifying assumption of conditionally independent residuals to allow different covariates to be associated with different responses. In future work, we would like to explore this multiple response extension to our model, using a hierarchical approach to allow information on the shared parameters to be pooled whilst incorporating correlation between the responses to aid variable selection.

## Supplementary material

Supplementary Material which contains the derivations of all of the analytical updates for the CAVI-MC is available online.

## Supplementary Information

**Additional file 1**. The derivation of the CAVI-MC updates, details of the RJMCMC moves and model proposals, properties of the constraint matrix, additional plots and tables.

## Data Availability

The Know Your Heart data that support the findings of this study are available from the the International Project on Cardiovascular Disease in Russia (IPCDR) Steering Group (at the London School of Hygiene and Tropical Medicine) but restrictions apply to the availability of these data, which were used under license for the current study, and so are not publicly available. Data are however available from the authors upon reasonable request and with permission of IPCDR Steering Group. Software in the form of Python code, together with a sample input data set and complete documentation is available on request from the corresponding author.

## Abbreviations

alr: Additive log-ratio
BMI: Body mass index
CAVI: Coordinate ascent variational inference
CAVI-MC: Monte Carlo coordinate ascent variational inference
clr: Centred log-ratio
DAG: Directed acyclic graph
ELBO: Evidence lower bound
ilr: Isometric log-ratio
MCMC: Markov chain Monte Carlo
OTU: Operational taxonomic unit
PE: Prediction error
RJMCMC: Reversible jump Monte Carlo Markov chain
SNR: Signal to noise ratio
