# Supplementary material for: Bayesian compositional regression with microbiome features via variational inference
Source: BMC Bioinformatics. 2023 May 22;24:210. doi: 10.1186/s12859-023-05219-x (PMC10201722; doi:10.1186/s12859-023-05219-x)
Supplement: Supplementary file 1 — Additional file 1. The derivation of the CAVI-MC updates, details of the RJMCMC moves and model proposals, properties of the constraint matrix, additional plots and tables. [file 12859_2023_5219_MOESM1_ESM.pdf]

Bayesian compositional regression with microbiome features via variational inference
Supplementary Material

DARREN A. V. SCOTT[1] and ALEX LEWIN[1]

[1]*London School of Hygiene and Tropical Medicine, London, UK*

Contents

1 CAVI-MC Updates **3**

1.1 Parametrisation 3

1.2 CAVI Updates 6

1.3 Pseudo Updates 25

1.4 ELBO 29

2 RJMCMC moves and model proposals **37**

2.1 Birth-death and swap moves 38

2.2 Within-model moves 40

3 Proofs **41**

3.1 Proof: Simplification of the constraint matrix 41

3.2 Proof: Eigenvalues of the constraint matrix 43

3.3 Proof: Pseudo Inverse of the constraint matrix 44

4 Tables **46**

5 Figures **50**

1 CAVI-MC Updates

This section contains all of the variation inference updates for the CAVI-MC.

1.1 Parametrisation

The full prior parametrisation is defined below. The likelihood and first level parameters are:

$$p(\mathbf{y}|\cdot) = \left(2\pi\sigma^2\right)^{-n/2} \exp\left(-\frac{1}{2\sigma^2} \|\mathbf{y} - \mathbf{1}_n\alpha - \mathbf{X}\boldsymbol{\beta} - \mathbf{W}\boldsymbol{\zeta} - \boldsymbol{Z}\boldsymbol{\theta}\|^2\right) \quad y \in \mathbb{R}^n \tag{1.1}$$

$$p(\alpha|w_\alpha) = (2\pi w_\alpha)^{-1/2} \exp\left(-\frac{1}{2w_\alpha}\alpha^2\right) \tag{1.2}$$

$$p(\beta_s|\gamma_s, w) = \left[(2\pi)^{-1/2} (w)^{-1/2} \exp\left\{-\frac{1}{2w} \|\beta_s\|^2\right\}\right]^{\gamma_s} \delta_0(\beta_s)^{1-\gamma_s} \quad \beta_s \in \mathbb{R}^1 \tag{1.3}$$

$$p(\gamma_s|\omega) = \omega^{\gamma_s}(1-\omega)^{1-\gamma_s} \quad \gamma_s \in \{0,1\} \tag{1.4}$$

$$p(\boldsymbol{\theta}|\cdot) = \frac{1}{\det^*(2\pi\mathbf{T}_\xi D(\boldsymbol{\psi}_\xi)\mathbf{T}_\xi^T)^{(1/2)}} \exp\left(-\frac{1}{2}(\boldsymbol{\theta}_\xi)^T(\mathbf{T}_\xi D(\boldsymbol{\psi}_\xi)\mathbf{T}_\xi^T)^{+}(\boldsymbol{\theta}_\xi)\right)\delta_0(\boldsymbol{\theta}_{\bar{\xi}}) \tag{1.5}$$

$$p(\boldsymbol{\psi}|\boldsymbol{\xi}) = \prod_{j=1}^{d} \left[\frac{b_\psi^{a_\psi}}{\Gamma(a_\psi)}(\psi_j)^{-a_\psi-1}\exp\{-b_\psi\psi_j^{-1}\}\right]^{\xi_j} \delta_0(\psi_j)^{1-\xi_j} \quad \psi_j > 0,\ \forall\ j \tag{1.6}$$

$$p(\boldsymbol{\zeta}_g|\chi_g, v) = \left(\frac{1}{(2\pi v)^{m_g/2}} \exp\left(-\frac{1}{2v}\boldsymbol{\zeta}_g^T\boldsymbol{\zeta}_g\right)\right)^{\chi_g} \delta_0(\boldsymbol{\zeta}_g)^{1-\chi_g} \tag{1.7}$$

$$p(\chi_g|\varrho) = \varrho^{\chi_g}(1-\varrho)^{1-\chi_g} \tag{1.8}$$

$$p(\sigma^2|\tau,\nu) = \frac{\nu^\tau}{\Gamma(\tau)}(\sigma^2)^{-\tau-1}\exp\{-\nu\sigma^{-2}\} \quad \sigma^2 > 0 \tag{1.9}$$

The hyperparameters are:

$$p(w_\alpha|a_\alpha, b_\alpha) = \frac{b_\alpha^{a_\alpha}}{\Gamma(a_\alpha)}(w_\alpha)^{-a_\alpha-1}\exp\{-b_\alpha w_\alpha^{-1}\} \quad w > 0 \tag{1.10}$$

$$p(b_\alpha) = \frac{b_{b\alpha}^{a_{b\alpha}}}{\Gamma(a_{b\alpha})}(b_\alpha^{a_{b\alpha}-1})\exp\{-b_{b\alpha}b_\alpha\} \quad b_\alpha > 0 \tag{1.11}$$

$$p(\omega|a_\omega, b_\omega) = \frac{1}{B(a_\omega, b_\omega)}\omega^{a_\omega-1}(1-\omega)^{b_\omega-1} \quad 0 \leq \omega \leq 1 \tag{1.12}$$

$$p(w|a_w, b_w) = \frac{b_w^{a_w}}{\Gamma(a_w)}(w)^{-a_w-1}\exp\{-b_w w^{-1}\} \quad w > 0 \tag{1.13}$$

$$p(b_w) = \frac{b_b^{a_b}}{\Gamma(a_b)}(b_w^{a_b-1})\exp\{-b_b b_w\} \quad b_w > 0 \tag{1.14}$$

$$p(\nu) = \frac{b_\nu^{a_\nu}}{\Gamma(a_\nu)}(\nu^{a_\nu-1})\exp\{-\nu b_\nu\} \tag{1.15}$$

$$p(\boldsymbol{\xi}) \propto \prod_{j=1}^{d}\kappa^{\xi_j}(1-\kappa)^{1-\xi_j}\mathbf{I}\left[\sum_j \xi_j \neq 1\right] \tag{1.16}$$

$$p(\kappa) = \frac{1}{B(a_\kappa, b_\kappa)}\kappa^{a_\kappa-1}(1-\kappa)^{b_\kappa-1} \quad 0 \leq \kappa \leq 1 \tag{1.17}$$

$$p(\varrho) = \frac{1}{B(a_\varrho, b_\varrho)}\varrho^{a_\varrho-1}(1-\varrho)^{b_\varrho-1} \quad 0 \leq \varrho \leq 1 \tag{1.18}$$

$$p(v|a_v, b_v) = \frac{b_v^{a_v}}{\Gamma(a_v)}(w)^{-a_v-1}\exp\{-b_v v^{-1}\} \quad v > 0 \tag{1.19}$$

$$p(b_v) = \frac{b_{bv}^{a_{bv}}}{\Gamma(a_{bv})}(b_v^{a_{bv}-1})\exp\{-b_{bv}b_v\} \quad b_v > 0 \tag{1.20}$$

The prior parametrisation is defined above, where the indexes s, j, g assign unique variables per index where as α, λ, τ and b assign single parameters. The design matrix $\boldsymbol{X}$ contains the continuous covariates, $\boldsymbol{W}$ contains the categorical covariates as dummy variables with reference to an intercept and $\boldsymbol{Z}$ contains the log microbiome data.

By imposing a constraint on θ we introduce a covariance between the elements θ_j which

we capture within the mean field family. The joint posterior is

$$
\begin{aligned}
p(\mathbf{y}, \boldsymbol{\vartheta}) =& p(\mathbf{y}|\cdot) \times \left\{ \prod_s p(\beta_s|w, \gamma_s) \times \prod_s p(\gamma_s|\omega) \right\} \times \left\{ \prod_g p(\boldsymbol{\zeta}_g, \chi_g) \times p(\chi_g|\varrho) \right\} \\
& \left\{ p(\boldsymbol{\theta}|\Sigma(\mathbf{T}, \boldsymbol{\psi}), \boldsymbol{\xi}) \times p(\boldsymbol{\psi}|\boldsymbol{\xi}) \times p(\boldsymbol{\xi}) \right\} \times p(\alpha|w_\alpha) \times p(w_\alpha|b_\alpha) \times p(b_\alpha) \\
& p(\omega) \times p(\kappa) \times p(\varrho) \times p(\sigma^2|\tau, \nu) \times p(w|b_w) \times p(b_w) \times p(\nu) \times p(v|b_v) \times p(b_v)
\end{aligned}
$$

Define the mean-field approximation distribution as

$$
\begin{aligned}
q(\boldsymbol{\vartheta}) =& q(\alpha) \times \left\{ \prod_s q(\beta_s, \gamma_s) \right\} \times \left\{ \prod_g q(\boldsymbol{\zeta}_g, \chi_g) \right\} \times q(\boldsymbol{\theta}, \boldsymbol{\psi}, \boldsymbol{\xi}) \times q(\omega) \times q(\kappa) \times q(\varrho) \times \\
& q(\sigma^2) \times q(w_\alpha) \times q(w) \times q(v) \times q(b_\alpha) \times q(b_w) \times q(b_v) \times q(\nu) \times q(\tau)
\end{aligned}
$$

with $f(\boldsymbol{\vartheta})^{(j)}$ as the j-th moment of $f(\boldsymbol{\vartheta})$ with respect to $q(\boldsymbol{\vartheta})$, $\mathbb{E}_q\left[f(\boldsymbol{\vartheta})^j\right]$.

By defining a block in the mean field approximation as a multivariate density $q(\theta, \xi)$, this allows us to incorporate correlation between the elements in θ (and the corresponding elements in ξ) related to the compositional explanatory variables and the correlation between θ_j and ξ_j. Now the expectation is with respect to the vector.

1.2 CAVI Updates

The CAVI update is proportional to

$$
\begin{aligned}
\log q(\alpha) \propto & \mathbb{E}_{(-\alpha)}\left[\log p(\mathbf{y}|.) + \log p(\alpha|w_\alpha)\right] \\
\propto & \mathbb{E}_{(-\alpha)}\Bigg[-\frac{1}{2\sigma^2}\Big\|\mathbf{y} - \alpha\mathbf{1}_n - \sum_s X_s\gamma_s\beta_s - \sum_j Z_j\xi_j\theta_j - \sum_g W_g\chi_g\boldsymbol{\zeta}_g\Big\|^2 + \\
& + \frac{1}{2}\log\big(w_\alpha^{-1}\big) - \frac{\alpha^2}{2w_\alpha}\Bigg] \\
& \propto -\frac{\alpha^2}{2(w_\alpha)^{(1)}} - \frac{1}{2(\sigma^2)^{(1)}}\Big(\alpha^2 n - 2\alpha\mathbf{1}_n^T\mathbf{y} + \\
& - 2\alpha\mathbf{1}_n^T\sum_s X_s(\beta_s)^{(1)} - 2\alpha\mathbf{1}_n^T\sum_j Z_j(\theta_j)^{(1)} - 2\alpha\mathbf{1}_n^T\sum_g W_g(\boldsymbol{\zeta}_g)^{(1)}\Big).
\end{aligned}
$$

By exponentiating and completing the square we have

$$
q(\alpha) = N(\mu_\alpha, \sigma_\alpha^2)
$$

with updates

$$
\mu_\alpha = \sigma_\alpha^2\Big[(\sigma^{-2})^{(1)}\mathbf{1}_n^T\Big(\mathbf{y} - \sum_s X_s(\beta_s)^{(1)} - (\boldsymbol{Z}_\xi\boldsymbol{\theta}_\xi)^{(1)} - \sum_g \boldsymbol{W}_g(\boldsymbol{\zeta}_g)^{(1)}\Big)\Big] \quad (1.21)
$$

$$
\sigma_\alpha^2 = \Big(n(\sigma^{-2})^{(1)} + (w_\alpha^{-1})^{(1)}\Big)^{-1} \quad (1.22)
$$

$$
\begin{aligned}
\log q(\beta_s,\gamma_s) =& \mathbb{E}_{(\beta_s,\gamma_s)}\Big[\log p(\mathbf{y}|.) + \log p(\beta_s|\gamma_s, w) + \log p(\gamma_s|\omega_s)\Big] + cst \\
=& \mathbb{E}_{(\beta_s,\gamma_s)}\Big[-\frac{1}{2\sigma^2}\Big\|\mathbf{y} - \alpha\mathbf{1}_n - \sum_{k\neq s} X_k\beta_k - X_s\beta_s - \boldsymbol{Z\theta} - \sum_g \boldsymbol{W}_g\boldsymbol{\zeta}_g\Big\|^2 - \frac{\gamma_s\beta_s^2}{2w} + \\
& + \gamma_s\log(2\pi w)^{-1/2} + \gamma_s\log(\omega) + (1-\gamma_s)(\log(1-\omega))\Big] + cst
\end{aligned}
$$

where cst is a constant with respect to β_s and γ_s. The spike-and-slab prior forces the latent selection variables into the likelihood component

$$
\begin{aligned}
\log(\beta_s, \gamma_s) =& \mathbb{E}_{(\beta_s,\gamma_s)}\Big[-\frac{1}{2\sigma^2}\Bigg(\|X_s\|^2\gamma_s\beta_s^2 + 2X_s^T\gamma_s\beta_s\sum_{k\neq s} X_k\gamma_k\beta_k - 2X_s^T\gamma_s\beta_s y + \\
& + 2X_s^T\gamma_s\beta_s \boldsymbol{Z}_\xi\boldsymbol{\theta}_\xi + 2X_s^T\gamma_s\beta_s\mathbf{1}_n\alpha + 2\gamma_s\beta_s X_s^T\sum_g \boldsymbol{W}_g\boldsymbol{\zeta}_g\chi_g\Bigg) - \frac{\gamma_s\beta_s^2}{2w} + \\
& + \gamma_s\log(2\pi w)^{-1/2} + \gamma_s\log(\omega) + (1-\gamma_s)(\log(1-\omega))\Big] + cst \\
=& -\frac{\gamma_s\beta_s^2}{2}\left(\frac{\|X_s\|^2}{(\sigma^2)^{(1)}} + \frac{1}{(w)^{(1)}}\right) + \gamma_s\beta_s\left(\frac{X_s^T}{(\sigma^2)^{(1)}}\left[\sum_{k\neq k} X_k(\beta_k)^{(1)} - \mathbf{y} + (\boldsymbol{Z}_\xi\boldsymbol{\theta}_\xi)^{(1)} + \right.\right. \\
& \left.\left. + \mathbf{1}_n(\alpha)^{(1)} + \sum_g \boldsymbol{W}_g(\boldsymbol{\zeta}_g)^{(1)}\right]\right) + \gamma_s\left(\frac{\log((w))^{(1)}}{2} + (\log\omega)^{(1)} - \frac{\log(2\pi)}{2}\right) + \\
& + (1-\gamma_s)((\log(1-\omega))^{(1)} + \delta_0(\beta_s)) + cst
\end{aligned}
$$

By exponentiating and completing the square we arrive at

$$
\begin{aligned}
q(\beta_s, \gamma_s|\mathbf{y}) =& \left[(2\pi\sigma_{\beta,s}^2)^{-\frac{1}{2}}\exp\left\{-\frac{1}{2\sigma_{\beta,s}^2}(\beta_s - \mu_{\beta,s})^2\right\}\right]^{\gamma_s} \times \qquad (1.23) \\
& \times \left[\left\{\exp\left((\log w^{-1})^{(1)}\right)\sigma_{\beta,s}^2\right\}^{\frac{1}{2}}\exp\left\{\frac{1}{2}\mu_{\beta,s}\sigma_{\beta,s}^{-2}\right\}\exp\left\{(\log\omega)^{(1)}\right\}\right]^{\gamma_s} \times \\
& \times \delta_0(\beta_s)^{1-\gamma_s}\exp\left\{(\log 1-\omega)^{(1)}\right\}^{1-\gamma_s}
\end{aligned}
$$

With updates

$$
\sigma_{\beta,s}^2 = \left[\|X_s\|^2(\sigma^{-2})^{(1)} + (w^{-1})^{(1)}\right]^{-1} \qquad (1.24)
$$

$$
\begin{aligned}
\mu_{\beta,s} &= \sigma_{\beta,s}^2 X_s^T\left[(\sigma^{-2})^{(1)}\left(\mathbf{y} - (\alpha)^{(1)}\mathbf{1}_n - \sum_{k\neq s} X_k(\beta_k)^{(1)} - (\boldsymbol{Z}_\xi\boldsymbol{\theta}_\xi)^{(1)} - \sum_g \boldsymbol{W}_g(\boldsymbol{\zeta}_g)^{(1)}\right)\right] \\
&= \sigma_{\beta,s}^2(\sigma^{-2})^{(1)}X_s^T(\boldsymbol{u}_{-s})^{(1)} \qquad (1.25)
\end{aligned}
$$

and thus by calling

$$(\gamma_s)^{(1)} = \left[1 + \sqrt{\sigma_{\beta,s}^{-2}}\exp\left\{(\log 1-\omega)^{(1)} - (\log\omega)^{(1)} - \frac{1}{2}(\log w^{-1})^{(1)} - \frac{1}{2}\mu_{\beta,s}^2\sigma_{\beta,s}^{-2}\right\}\right]^{-1} \tag{1.26}$$

we have under q

$$q(\beta_s|\gamma_s = 1, y) = \mathcal{N}(\mu_{\beta,s}, \sigma_{\beta,s}^2), \quad q(\beta_s|\gamma_s = 0, y) = \delta_0(\beta_s)$$
$$q(\gamma_s|y) = Bern(\gamma_s^{(1)}).$$

Note that now

$$(\beta_s)^{(1)} = \mu_{\beta,s}(\gamma_s)^{(1)} \tag{1.27}$$

$$(\beta_s)^{(2)} = (\sigma_{\beta,s}^2 + \mu_{\beta,s}^2)(\gamma_s)^{(1)}. \tag{1.28}$$

The index g denotes the categorical factor groupings $g = 1, ..., G$ and m_g is the dimension of the vector $\boldsymbol{\zeta}_g$. As the categorical factors are coded with reference to the intercept, m_g is always 1 less than the levels in the categorical factor.

$$\begin{aligned}\log q(\boldsymbol{\zeta}_g, \chi_g) =& \mathbb{E}_{(\boldsymbol{\zeta}_g, \chi_g)}\Big[\log p(\mathbf{y}|.) + \log p(\boldsymbol{\zeta}_g|\chi_g, v) + \log p(\chi_g|\varrho)\Big] + cst \\ =& \mathbb{E}_{(\zeta_g, \chi_g)}\Big[-\frac{1}{2\sigma^2}\Big\|\mathbf{y} - \alpha\mathbf{1}_n - \boldsymbol{X\beta} - \sum_{k\neq g}\boldsymbol{W}_k\boldsymbol{\zeta}_k - \boldsymbol{W}_g\boldsymbol{\zeta}_g - \boldsymbol{Z\theta}\Big\|^2 - \frac{\chi_g\boldsymbol{\zeta}_g^T\boldsymbol{\zeta}_g}{2v} + \\ &+ \chi_g\log(2\pi v)^{-1/2} + \chi_g\log(\varrho) + (1-\chi_g)(\log(1-\varrho))\Big] + cst\end{aligned}$$

where cst is a constant with respect to $\boldsymbol{\zeta}_g$ and χ_g. The spike-and-slab prior forces the

latent selection variables into the likelihood component

$$
\begin{aligned}
\log(\boldsymbol{\zeta}_g, \chi_g) =& \mathbb{E}_{(\boldsymbol{\zeta}_g, \chi_g)} \Big[-\frac{1}{2\sigma^2} \Bigg(\chi_g \boldsymbol{\zeta}_g^T \boldsymbol{W}_g^T \boldsymbol{W}_g \boldsymbol{\zeta}_g - 2\chi_g \boldsymbol{\zeta}_g^T \boldsymbol{W}_g^T (\mathbf{y} - \alpha \mathbf{1}_n - \sum_s X_s \gamma_s \beta_s + \\
& - \boldsymbol{Z}_\xi \boldsymbol{\theta}_\xi - \sum_k \boldsymbol{W}_k \boldsymbol{\zeta}_k \chi_k) - \frac{\chi_g \boldsymbol{\zeta}_g^T \boldsymbol{\zeta}_g}{2v} + \chi_g \log(2\pi v)^{-m_g/2} + \chi_g \log(\varrho) + \\
& + (1 - \chi_g)(\log(1 - \varrho)) \Big] + cst \\
=& \chi_g \Bigg(\frac{-m_g}{2} (\log 2\pi) - \frac{1}{2} \Bigg(\frac{1}{(v)^{(1)}} \boldsymbol{\zeta}_g^T \boldsymbol{\zeta}_g + \frac{1}{(\sigma^2)^{(1)}} \boldsymbol{\zeta}_g^T \boldsymbol{W}_g^T \boldsymbol{W}_g \boldsymbol{\zeta}_g + \\
& - 2 \frac{1}{(\sigma^2)^{(1)}} \boldsymbol{\zeta}_g^T \boldsymbol{W}_g^T (\boldsymbol{u}_{-g})^{(1)} \Bigg) + \chi_g (\log \varrho)^{(1)} + \frac{m_g}{2} (\log v^{-1})^{(1)}) + \\
& + (1 - \chi_g)(\log(1 - \rho_g))^{(1)} + \delta_0(\boldsymbol{\zeta}_g) + cst
\end{aligned}
$$

defining

$$
\Sigma_{\zeta_g} = \left[(\sigma^{-2})^{(1)} \boldsymbol{W}_g^T \boldsymbol{W}_g + (v^{-1})^{(1)} \mathbf{I}_{m_g} \right]^{-1} \tag{1.29}
$$

$$
\boldsymbol{\mu}_{\zeta_g} = (\sigma^{-2})^{(1)} \Sigma_{\zeta_g} \boldsymbol{W}_g^T (\boldsymbol{u}_{-g})^{(1)} \tag{1.30}
$$

by exponentiating, completing the square we have

$$
\begin{aligned}
q(\boldsymbol{\zeta}_g, \chi_g) =& \left[\frac{1}{(2\pi)^{m_g/2}} \det(\Sigma_{\zeta_g})^{-1/2} \exp\left\{ -\frac{1}{2} (\boldsymbol{\zeta}_g - \boldsymbol{\mu}_{\zeta_g})^T \Sigma_{\zeta_g}^{-1} (\boldsymbol{\zeta}_g - \boldsymbol{\mu}_{\zeta_g}) \right\} \right]^{\chi_g} \times \delta_0(\boldsymbol{\zeta}_g)^{1-\chi_g} \\
& \left[\exp\left(\frac{1}{2} \boldsymbol{\mu}_{\zeta_g}^T \Sigma_{\zeta_g}^{-1} \boldsymbol{\mu}_{\zeta_g} + \frac{1}{2} \log\det(\Sigma_{\zeta_g}) + \frac{m_g}{2} (\log v^{-1})^{(1)} + (\log \varrho)^{(1)} \right) \right]^{\chi_g} \times \\
& \left[\exp\left((\log(1 - \varrho))^{(1)} \right) \right]^{1-\chi_g},
\end{aligned}
$$

and thus by calling

$$
\begin{aligned}
(\chi_g)^{(1)} =& \Big[1 + \exp\Big((\log 1 - \varrho)^{(1)} - (\log \varrho)^{(1)} - \frac{m_g}{2}(\log \upsilon^{-1})^{(1)} - \frac{1}{2}\boldsymbol{\mu}_{\zeta_g}^T \Sigma_{\zeta_g}^{-1} \boldsymbol{\mu}_{\zeta_g} + \\
& - \frac{1}{2}\log\big(\det(\Sigma_{\zeta_g})\big)\Big)\Big]^{-1}
\end{aligned}
$$

we have under q

$$
\begin{aligned}
q(\boldsymbol{\zeta}_g|\chi_g = 1, y) &= \mathcal{N}_{m_g}(\boldsymbol{\mu}_{\zeta_g}, \Sigma_{\zeta_g}), \quad q(\boldsymbol{\zeta}_g|\chi_g = 0, y) = \delta_0(\boldsymbol{\zeta}_g) \\
q(\chi_g|y) &= Bern((\chi_g)^{(1)}).
\end{aligned}
$$

Note that now

$$
(\boldsymbol{\zeta}_g)^{(1)} = \boldsymbol{\mu}_\zeta (\chi_g)^{(1)} \tag{1.31}
$$

$$
(\boldsymbol{\zeta}_g^T \boldsymbol{\zeta}_g)^{(1)} = (\mathrm{tr}(\Sigma_{\zeta_g}) + \boldsymbol{\mu}_{\zeta_g}^T \boldsymbol{\mu}_{\zeta_g})(\chi_g)^{(1)} \tag{1.32}
$$

$$
(\boldsymbol{\zeta}_g^T \boldsymbol{W}_g^T \boldsymbol{W}_g \boldsymbol{\zeta}_g)^{(1)} = (\mathrm{tr}(\boldsymbol{W}_g \Sigma_{\zeta_g} \boldsymbol{W}_g^T) + \boldsymbol{\mu}_{\zeta_g}^T \boldsymbol{W}_g^T \boldsymbol{W}_g \boldsymbol{\mu}_{\zeta_g})(\chi_g)^{(1)} \tag{1.33}
$$

$$
\log q(\boldsymbol{\theta}, \boldsymbol{\psi}, \boldsymbol{\xi}|\cdot) = \mathbb{E}_{(\theta,\psi,\xi)}\Big[\log p(\mathbf{y}|.) + \log p(\boldsymbol{\theta}|\boldsymbol{\psi}, \boldsymbol{\xi}) + \log p(\boldsymbol{\psi}|\boldsymbol{\xi}) + \log p(\boldsymbol{\xi})\Big] + cst
$$

$$
\begin{aligned}
\log q(\boldsymbol{\theta}, \boldsymbol{\psi}, \boldsymbol{\xi}|\cdot) \propto\ & \mathbb{E}_{-(\xi,\psi,\theta)}\Bigg[-\frac{1}{2}\Big(\boldsymbol{\theta}_\xi^T(\mathbf{T}_\xi D(\boldsymbol{\psi}_\xi)\mathbf{T}_\xi)^{+}\boldsymbol{\theta}_\xi + \sigma^{-2}\Big\|\mathbf{y} - \alpha\mathbf{1}_n - \boldsymbol{X\beta} - \boldsymbol{Z}_\xi\boldsymbol{\theta}_\xi + \\
& - \boldsymbol{W\zeta}\Big\|^2\Big) - \frac{1}{2}(d_\xi - 1)\log(2\pi) - \frac{1}{2}\log(\det{}^*(\mathbf{T}_\xi D(\boldsymbol{\psi}_\xi)\mathbf{T}_\xi))\Bigg]_{[I(\sum_j \theta_j = 0)]} + \qquad (1.34) \\
& + \mathbb{E}_{-(\xi,\psi,\theta)}\Bigg[\sum_j\Bigg(\xi_j\log(\kappa) + (1-\xi_j)\log(1-\kappa)\Bigg) + \log\delta(\theta_{\bar{\xi}}) + \sum_j \xi_j(a_\psi\log(b_\psi)) + \\
& - \sum_j \xi_j\log(\Gamma(a_\psi)) - \sum_j(a_\psi+1)\xi_j\log(\psi_j) - b_\psi\sum_j(1-\xi_j)\psi_j^{-1}\Bigg]
\end{aligned}
$$

which we express as

$$
\log p(\boldsymbol{\theta}, \boldsymbol{\psi}, \boldsymbol{\xi}|\mathbf{y}, \cdot) \propto A + B \qquad (1.35)
$$

where each capital letter refers to the expression within the parenthesis of the expectations in equation (1.34).

$$
\begin{aligned}
A \propto & -\frac{1}{2}(d_\xi - 1)\log(2\pi) - \frac{1}{2}\log(\det{}^*(\mathbf{T}_\xi D(\boldsymbol{\psi}_\xi)\mathbf{T}_\xi)) + \\
& -\frac{1}{2}\Bigg(\boldsymbol{\theta}_\xi^T(\mathbf{T}_\xi D(\boldsymbol{\psi}_\xi)\mathbf{T}_\xi)^{+}\boldsymbol{\theta}_\xi + \sigma^{-2}\Bigg(\boldsymbol{\theta}_\xi^T\boldsymbol{Z}_\xi^T\boldsymbol{Z}_\xi\boldsymbol{\theta}_\xi - 2\boldsymbol{\theta}_\xi^T\boldsymbol{Z}_\xi^T(\mathbf{y} - \alpha\mathbf{1}_n - \boldsymbol{X\beta} - \boldsymbol{W\zeta})\Bigg), \qquad (1.36)
\end{aligned}
$$

define

$$
\boldsymbol{u}_{\not{f}} = \mathbf{y} - \alpha\mathbf{1}_n - \sum_s X_s\gamma_s\beta_s - \sum_g \boldsymbol{W}_g\boldsymbol{\zeta}_g \qquad (1.37)
$$

and the vector $\boldsymbol{\mu}_{\theta_\xi}$ and matrix Σ_{θ_ξ}

$$\boldsymbol{\mu}_{\theta_\xi} = \Sigma_{\theta_\xi}(\sigma^{-2})^{(1)}\boldsymbol{Z}_\xi^T(\mathbf{u}_{\not y})^{(1)} \tag{1.38}$$

$$\Sigma_{\theta_\xi} = \left((\mathbf{T}_\xi D(\boldsymbol{\psi}_\xi)\mathbf{T}_\xi)^+ + (\sigma^{-2})^{(1)}\boldsymbol{Z}_\xi^T\boldsymbol{Z}_\xi\right)^{-1} \tag{1.39}$$

Unlike in the β_s updates for the free variational parameters, these are still function of the vector $\boldsymbol{\xi}$. On completing the square we have

$$\boldsymbol{\theta}_\xi^T\Sigma_{\theta_\xi}^{-1}\boldsymbol{\theta}_\xi - 2\boldsymbol{\theta}_\xi^T(\Sigma_{\theta_\xi}^{-1})\boldsymbol{\mu}_{\theta_\xi} = (\boldsymbol{\theta}_\xi - \boldsymbol{\mu}_{\theta_\xi})^T\Sigma_{\theta_\xi}^{-1}(\boldsymbol{\theta}_\xi - \boldsymbol{\mu}_{\theta_\xi}) - \boldsymbol{\mu}_{\theta_\xi}^T\Sigma_{\theta_\xi}^{-1}\boldsymbol{\mu}_{\theta_\xi}$$

$$\begin{aligned}
\log q(\boldsymbol{\theta}, \boldsymbol{\psi}, \boldsymbol{\xi}|\mathbf{y}, .) \propto & \Bigg[-\frac{1}{2}(d_\xi - 1)\log 2\pi - \frac{1}{2}\log(\det{}^*(\mathbf{T}_\xi D(\boldsymbol{\psi}_\xi)\mathbf{T}_\xi)) + \\
& -\frac{1}{2}\left([\boldsymbol{\theta}_\xi - \boldsymbol{\mu}_{\theta_\xi}]^T\Sigma_{\theta_\xi}^{-1}[\boldsymbol{\theta}_\xi - \boldsymbol{\mu}_{\theta_\xi}]\right) - \boldsymbol{\mu}_{\theta_\xi}^T\Sigma_{\theta_\xi}^{-1}\boldsymbol{\mu}_{\theta_\xi}\Bigg]_{[I(\sum_j \theta_{\xi_j}=0)]} + \\
& + \sum_j \xi_j(\log\kappa)^{(1)} + \sum_j(1-\xi_j)(\log(1-\kappa))^{(1)} + (a_\psi\log(b_\psi) + \\
& - \log(\Gamma(a_\psi))\sum_j \xi_j - \sum_j(a_\psi + 1)\xi_j\log(\psi_j) - b_\psi\sum_j \xi_j\psi_j^{-1}
\end{aligned}$$

We can remove the index by adding the constraint on μ_{θ_ξ} and Σ_{θ_ξ} with the matrix T_ξ.

$$\begin{aligned}
\log q(\boldsymbol{\theta}, \boldsymbol{\psi}, \boldsymbol{\xi}|\boldsymbol{y}, .) \propto & -\frac{1}{2}\log(\det{}^*(\mathbf{T}_\xi D(\boldsymbol{\psi}_\xi)\mathbf{T}_\xi)) + \sum_j \xi_j(\log\kappa)^{(1)} + \sum_j(1-\xi_j)(\log\kappa)^{(1)} + \\
& -\frac{1}{2}(d_\xi - 1)\log(2\pi) - \frac{1}{2}\left([\boldsymbol{\theta}_\xi - \mathbf{T}_\xi\boldsymbol{\mu}_{\theta_\xi}]^T(\mathbf{T}_\xi\Sigma_{\theta_\xi}\mathbf{T}_\xi)^+[\boldsymbol{\theta}_\xi - \mathbf{T}_\xi\boldsymbol{\mu}_{\theta_\xi}]\right) + \\
& + \frac{1}{2}\boldsymbol{\mu}_{\theta_\xi}^T\mathbf{T}_\xi^T(\mathbf{T}_\xi^T\Sigma_{\theta_\xi}\mathbf{T}_\xi)^+\mathbf{T}_\xi\boldsymbol{\mu}_{\theta_\xi} - \sum_j(a_\psi + 1)\xi_j\log(\psi_j) - b_\psi\sum_j \xi_j\psi_j^{-1} + \\
& + (a_\psi\log(b_\psi) - \log(\Gamma(a_\psi)))\sum_j \xi_j
\end{aligned}$$

We can then identify the singular multivariate normal density

$$\log q(\boldsymbol{\theta}, \boldsymbol{\psi}, \boldsymbol{\xi}|\boldsymbol{y}, .) \propto -\frac{1}{2}(d_\xi - 1)\log(2\pi) - \frac{1}{2}\log\big(\det{}^*(\mathbf{T}_\xi \Sigma_{\theta_\xi} T_\xi)\big) + \frac{1}{2}\log\big(\det{}^*(\mathbf{T}_\xi \Sigma_{\theta_\xi} \mathbf{T}_\xi)\big) +$$
$$- \frac{1}{2}\left([\boldsymbol{\theta}_\xi - \mathbf{T}_\xi \boldsymbol{\mu}_{\theta_\xi}]^T (\mathbf{T}_\xi \Sigma_{\theta_\xi} T_\xi)^+ [\boldsymbol{\theta}_\xi - \mathbf{T}_\xi \boldsymbol{\mu}_{\theta_\xi}] \right) + \frac{1}{2}\boldsymbol{\mu}_{\theta_\xi}^T \mathbf{T}_\xi^T (\mathbf{T}_\xi^T \Sigma_{\theta_\xi} \mathbf{T}_\xi)^+ \mathbf{T}_\xi \boldsymbol{\mu}_{\theta_\xi} +$$
$$- \frac{1}{2}\log(\det{}^*(\mathbf{T}_\xi D(\boldsymbol{\psi}_\xi)\mathbf{T}_\xi)) + \sum_j \xi_j (\log\kappa)^{(1)} + \sum_j (1-\xi_j)(\log(1-\kappa))^{(1)} +$$
$$- \sum_j (a_\psi + 1)\xi_j \log(\psi_j) - b_\psi \sum_j \xi_j \psi_j^{-1} + (a_\psi \log(b_\psi) - \log(\Gamma(a_\psi)) \sum_j \xi_j$$

which can be expressed as

$$q(\boldsymbol{\theta}, \boldsymbol{\psi}, \boldsymbol{\xi}|\boldsymbol{y}, .) \propto \ \mathrm{SMVN}_{d_\xi}(\mathbf{T}_\xi \boldsymbol{\mu}_{\theta_\xi}, \mathbf{T}_\xi \Sigma_{\theta_\xi} \mathbf{T}_\xi)\delta(\bar{\xi}) \times \tag{1.40}$$
$$\exp\left(\frac{1}{2}\boldsymbol{\mu}_{\theta_\xi}^T \mathbf{T}_\xi (\mathbf{T}_\xi^T \Sigma_{\theta_\xi} \mathbf{T}_\xi)^+ \mathbf{T}_\xi \boldsymbol{\mu}_{\theta_\xi} + \frac{1}{2}\log\big(\det{}^*(\mathbf{T}_\xi \Sigma_{\theta_\xi} \mathbf{T}_\xi)\big) + \sum_j \xi_j (\log\kappa)^{(1)} \right.$$
$$- \frac{1}{2}\log(\det{}^*(\mathbf{T}_\xi D(\boldsymbol{\psi}_\xi)\mathbf{T}_\xi)) + \sum_j (1-\xi_j)(\log(1-\kappa))^{(1)} - \sum_j (a_\psi + 1)\xi_j \log(\psi_j) +$$
$$\left. - b_\psi \sum_j \xi_j \psi_j^{-1} + (a_\psi \log(b_\psi) - \log(\Gamma(a_\psi)) \sum_j \xi_j \right) \tag{1.41}$$

We can identify the singular multivariate normal density (1.40) which is a function of $\boldsymbol{\xi}$ and $\boldsymbol{\psi}$. The $\boldsymbol{\xi}$ and $\boldsymbol{\psi}$ component (1.41) contains terms which do not have a conjugate update. The first term

$$\boldsymbol{\mu}_{\theta_\xi}^T \mathbf{T}_\xi (\mathbf{T}_\xi^T \Sigma_{\theta_\xi} \mathbf{T}_\xi)^+ \mathbf{T}_\xi \boldsymbol{\mu}_{\theta_\xi} \tag{1.42}$$

has dependencies on $\boldsymbol{\xi}$ in $\boldsymbol{\mu}_{\theta_\xi}$ and Σ_{θ_ξ} which are a function of $\boldsymbol{\psi}$ and the remaining q expectations.

Thus

$$q(\boldsymbol{\theta}_\xi|\boldsymbol{\psi}, \boldsymbol{\xi}) = \ \mathrm{SMVN}(\mathbf{T}_\xi \mu_{\theta_\xi}, \mathbf{T}_\xi \Sigma_{\theta_\xi} \mathbf{T}_\xi) \quad \text{and} \quad q(\boldsymbol{\theta}_{\bar{\xi}} = 0|\boldsymbol{\xi}) = 1, \tag{1.43}$$

or

$$q(\boldsymbol{\theta}|\boldsymbol{\xi},\boldsymbol{\psi}) = \text{ SMVN}(\mathbf{T}_\xi \mu_{\theta_\xi}, \mathbf{T}_\xi \Sigma_{\theta_\xi} \mathbf{T}_\xi)\delta(\boldsymbol{\theta}_{\bar{\xi}}) \tag{1.44}$$

and

$$\begin{aligned}\log q(\boldsymbol{\theta},\boldsymbol{\psi},\boldsymbol{\xi}|\boldsymbol{y},.) \propto \log\big(\text{SMVN}(\mathbf{T}_\xi \boldsymbol{\mu}_{\theta_\xi}, \mathbf{T}_\xi \Sigma_{\theta_\xi} \mathbf{T}_\xi)\big) + \frac{1}{2}\mu_{\theta_\xi}^T \mathbf{T}_\xi (\mathbf{T}_\xi^T \Sigma_{\theta_\xi} \mathbf{T}_\xi)^{+} \mathbf{T}_\xi \boldsymbol{\mu}_{\theta_\xi} + \\ + \frac{1}{2}\log\big(\det{}^*(\mathbf{T}_\xi \Sigma_{\theta_\xi} \mathbf{T}_\xi)\big) - \frac{1}{2}\log(\det{}^*(\mathbf{T}_\xi D(\boldsymbol{\psi}_\xi)\mathbf{T}_\xi)) + \sum_j \xi_j (\log\kappa)^{(1)} + \\ + \sum_j (1-\xi_j)(\log(1-\kappa))^{(1)} - \sum_j (a_\psi + 1)\xi_j \log(\psi_j) - b_\psi \sum_j \xi_j \psi_j^{-1} + \\ + (a_\psi \log(b_\psi) - \log(\Gamma(a_\psi)) \sum_j \xi_j\end{aligned} \tag{1.45}$$

For w we have

$$\log q(w) = \mathbb{E}_{-w}\left[\sum_s \log p(\beta_s|w,\gamma_s) + \log p(w|a_w,b_w)\right] + cst$$

$$\begin{aligned} q(w) =& \mathbb{E}_{-w}\Big[\sum_s -\frac{\gamma_s}{2}\Big(\log w - w^{-1}\frac{\beta_s^2}{2}\Big)(-a_w - 1)\log w - b_w w^{-1}\Big] + cst \\ &\propto \log w\Big(-\frac{1}{2}\Big\{\sum_s (\gamma_s)^{(1)}\Big\} - a_w - 1\Big) - w^{-1}\Big(\frac{1}{2}\Big\{\sum_s (\beta_s)^{(2)}\Big\} + (b_w)^{(1)}\Big)\end{aligned} \tag{1.46}$$

thus

$$q(w) = Inv - Gamma(a_w^*, b_w^*) \tag{1.47}$$

with parameters

$$a_w^* = \frac{1}{2}\Big\{ \sum_s (\gamma_s)^{(1)} \Big\} + a_w \tag{1.48}$$

$$b_w^* = \frac{1}{2}\Big\{ \sum_s (\beta_s)^{(2)} + \Big\} + (b_w)^{(1)} \tag{1.49}$$

For v we have

$$\log q(v) = \mathbb{E}_{-v}\left[\sum_g \log p(\boldsymbol{\zeta}_g|v, \chi_g) + \log p(v|a_v, b_v)\right] + cst$$

$$\begin{aligned} q(v) =& \mathbb{E}_{-v}\Big[\sum_g \chi_g\Big(-\frac{m_g}{2}\log v - v^{-1}\frac{\boldsymbol{\zeta}_g^T\boldsymbol{\zeta}_g}{2}\Big) + (-a_v - 1)\log v - b_v v^{-1}\Big] + cst \\ &\propto \log v\Big(-\frac{1}{2}\Big\{\sum_g m_g(\chi_g)^{(1)}\Big\} - a_v - 1\Big) - v^{-1}\Big(\frac{1}{2}\Big\{\sum_g (\chi_g\boldsymbol{\zeta}_g^T\boldsymbol{\zeta}_g)^{(1)}\Big\} + (b_v)^{(1)}\Big) \end{aligned}$$

thus

$$q(v) = Inv - Gamma(a_v^*, b_v^*) \tag{1.50}$$

with parameters

$$a_v^* = \frac{1}{2}\Big\{ \sum_g m_g(\chi_g)^{(1)} \Big\} + a_v \tag{1.51}$$

$$b_v^* = \frac{1}{2}\Big\{ \sum_g (\boldsymbol{\zeta}_g^T\boldsymbol{\zeta}_g)^{(1)} \Big\} + (b_v)^{(1)} \tag{1.52}$$

$$\log q(\omega) \propto \mathbb{E}_{-\omega}\left[\log \prod_s p(\gamma_s|\omega) + \log p(\omega)\right] \tag{1.53}$$

$$
\begin{aligned}
\log q(\omega) &\propto \sum_s (\gamma_s)^{(1)} \log \omega + \sum_s (1-\gamma_s)^{(1)} \log(1-\omega) + (a_\omega - 1)\log\omega + (b_\omega - 1)\log(1-\omega) \\
&\propto \left(a_\omega + \sum_s (\gamma_s)^{(1)} - 1\right) \log\omega + \left(b_{\omega,s} + p - \sum_s (\gamma_s)^{(1)} - 1\right) \log(1-\omega).
\end{aligned}
$$

which implies that

$$
q(\omega) = Beta(a_w^*, b_w^*) \tag{1.54}
$$

with parameters

$$
a_\omega^* = a_\omega + \sum_s (\gamma_s)^{(1)} \tag{1.55}
$$

$$
b_\omega^* = b_\omega + p - \sum_s (\gamma_s)^{(1)} \tag{1.56}
$$

where

$$
\begin{aligned}
(\omega)^{(1)} &= a_\omega^* / \left(a_\omega^* + b_\omega^*\right) = a_\omega^* / \left(a_\omega + b_\omega + p\right) \qquad (1.57) \\
(\log\omega)^{(1)} &= \Psi(a_\omega^*) - \Psi(a_\omega^* + b_\omega^*) \\
(\log(1-\omega))^{(1)} &= \Psi(b_\omega^*) - \Psi(a_\omega^* + b_\omega^*)
\end{aligned}
$$

where $\Psi(\cdot)$ is the digamma function.

$$
\log q(w_\alpha) = \mathbb{E}_{-w_\alpha}\left[\log p(\alpha|, w_\alpha)) + \log p(w_\alpha | a_\alpha, b_\alpha)\right] + cst \tag{1.58}
$$

$$
\begin{aligned}
\log q(w_\alpha) &= \frac{1}{2}\log\left(w_\alpha^{-1}\right) - \frac{w_\alpha^{-1}}{2}\alpha^2 + (a_\alpha + 1)\log\left(w_\alpha^{-1}\right) - w_\alpha^{-1}(b_\alpha)^{(1)} + cst \\
&= \left(a_\alpha + \frac{1}{2}\right)\log\left(w_\alpha^{-1}\right) - w_\alpha^{-1}\left((b_\alpha)^{(1)} + \frac{1}{2}(\alpha)^{(2)}\right) + cst
\end{aligned}
$$

Thus we have

$$q(w_\alpha) = Inv - Gamma(a^*_\alpha, b^*_\alpha) \tag{1.59}$$

with parameters

$$a^*_\alpha = a_\alpha + \frac{1}{2} \tag{1.60}$$

$$b^*_\alpha = (b_\alpha)^{(1)} + \frac{1}{2}(\alpha)^{(2)} \tag{1.61}$$

where

$$(w_\alpha^{-1})^{(1)} = a^*_\alpha / b^*_\alpha \tag{1.62}$$

$$(\log w_\alpha^{-1})^{(1)} = \Psi(a^*_\alpha) - \log b^*_\alpha. \tag{1.63}$$

$$\log q(b_w) = \mathbb{E}_{-b_w}\Big[\log p(w|a_w, b_w) + \log p(b_w|a_b, b_b)\Big] \tag{1.64}$$

$$\begin{aligned}\log q(b_w) &= \mathbb{E}_{-b_w}\Big[a_w \log b_w - b_w w^{-1} + (a_b - 1)\log b_w - b_b b_w\Big] + cst \\ &= a_w \log b_w - b_w (w)^{(-1)} + (a_b - 1)\log b_w - b_b b_w + cst \\ &= \log b_w (a_w + a_b - 1) - b_w((w)^{(-1)} + b_b) + cst \end{aligned} \tag{1.65}$$

thus

$$q(b_w) = Gamma(\text{shape} = a^*_b, \text{rate} = b^*_b)$$

with parameters

$$a_b^* = a_w + a_b \tag{1.66}$$

$$b_b^* = (w)^{(-1)} + b_b \tag{1.67}$$

where

$$(b_w)^{(1)} = a_b^*/b_b^* \tag{1.68}$$

$$(\log b_w)^{(1)} = \Psi(a_b^*) - \log b_b^* \tag{1.69}$$

$$\log q(b_\alpha) = \mathbb{E}_{-b_\alpha}\Bigg[\log p(w_\alpha|a_\alpha, b_\alpha) + \log p(b_\alpha|a_{b,\alpha}, b_{b,\alpha})\Bigg] \tag{1.70}$$

$$\begin{aligned}\log q(b_\alpha) &= \mathbb{E}_{-b_\alpha}\Bigg[a_\alpha \log b_\alpha - b_\alpha w_\alpha^{-1} + (a_{b,\alpha} - 1)\log b_\alpha - b_{b,\alpha} b_\alpha\Bigg] + cst \\ &= \log b_\alpha(a_\alpha + a_{\alpha,b} - 1) - b_\alpha((w_\alpha)^{(-1)} + b_{\alpha,b}) + cst \end{aligned} \tag{1.71}$$

thus

$$q(b_\alpha) = Gamma(\text{shape} = a_{b,\alpha}^*, \text{rate} = b_{b,\alpha}^*)$$

with parameters

$$a^*_{b,\alpha} = a_\alpha + a_{b,\alpha} \tag{1.72}$$

$$b^*_{b,\alpha} = (w_\alpha)^{(-1)} + b_{b,\alpha} \tag{1.73}$$

where

$$(b_\alpha)^{(1)} = a^*_{b,\alpha}/b^*_{b,\alpha} \tag{1.74}$$

$$(\log b_\alpha)^{(1)} = \Psi(a^*_{b,\alpha}) - \log b^*_{b,\alpha} \tag{1.75}$$

$$\log q(b_v) = \mathbb{E}_{-b_v}\Bigg[\log p(v|a_v, b_v) + \log p(b_v|a_{bv}, b_{bv})\Bigg] \tag{1.76}$$

$$\begin{aligned}\log q(b_v) &= \mathbb{E}_{-b_v}\Bigg[a_v \log b_v - b_v v^{-1} + (a_{bv} - 1)\log b_v - b_{bv} b_v\Bigg] + cst \\ &= a_v \log b_v - b_v (v^{-1})^{(1)} + (a_{bv} - 1)\log b_v - b_{bv} b_v + cst \\ &= \log b_v (a_v + a_{bv} - 1) - b_v (v^{-1})^{(1)} + b_{bv}) + cst \end{aligned} \tag{1.77}$$

thus

$$q(b_v) = Gamma(shape = a^*_v, rate = b^*_v)$$

with parameters

$$a^*_{bv} = a_v + a_{bv} \tag{1.78}$$

$$b^*_{bv} = (v^{-1})^{(1)} + b_{bv} \tag{1.79}$$

where

$$(b_v)^{(1)} = a^*_{bv}/b^*_{bv} \tag{1.80}$$

$$(\log b_v)^{(1)} = \Psi(a^*_{bv}) - \log b^*_{bv} \tag{1.81}$$

$$\log q(\sigma^2) = \mathbb{E}_{-\sigma^2}[\log p(\sigma^2|\tau,\nu)] + \mathbb{E}_{-\sigma^2}[\log p(\mathbf{y}|\boldsymbol{\beta},\boldsymbol{\theta},\boldsymbol{\zeta},\sigma^2)] + cst$$

Using $\mathbb{E}_q[\boldsymbol{Z}_\xi\boldsymbol{\theta}_\xi] = \boldsymbol{Z}(\boldsymbol{\theta})^{(1)}$ and

$$\begin{aligned}\mathbb{E}_q[\boldsymbol{\zeta}_g^T\boldsymbol{W}_g^T\boldsymbol{W}_g\boldsymbol{\zeta}_g\chi_g|\chi_g] &= \mathbb{E}_q[\boldsymbol{\zeta}_g^T\boldsymbol{W}_g^T\boldsymbol{W}_g\boldsymbol{\zeta}_g|\chi_g]\chi_g \\ &= (\mathrm{tr}(\boldsymbol{W}_g\Sigma_{\zeta_g}\boldsymbol{W}_g) + \mathbb{E}_q[\boldsymbol{\zeta}_g^T|\chi_g]\boldsymbol{W}_g^T\boldsymbol{W}_g\mathbb{E}_q[\boldsymbol{\zeta}_g|\chi_g])\chi_g\end{aligned}$$

so $\mathbb{E}_g[\boldsymbol{\zeta}_g^T\boldsymbol{W}_g^T\boldsymbol{W}_g\boldsymbol{\zeta}\chi_g]$ referred to as $(\boldsymbol{\zeta}_g^T\boldsymbol{W}_g^T\boldsymbol{W}_g\boldsymbol{\zeta})^{(1)}$ and

$$\begin{aligned}\mathbb{E}_q[\mathbb{E}_q[\boldsymbol{\zeta}_g^T\boldsymbol{W}_g^T\boldsymbol{W}_g\boldsymbol{\zeta}\chi_g|\chi_g]] &= \left(\mathrm{tr}(\boldsymbol{W}_g\Sigma_{\zeta_g}\boldsymbol{W}_g^T) + \boldsymbol{\mu}_{\zeta_g}^T\boldsymbol{W}_g^T\boldsymbol{W}_g\boldsymbol{\mu}_{\zeta_g}\right)(\chi_g)^{(1)} \\ &= (\boldsymbol{\zeta}_g^T\boldsymbol{W}_g^T\boldsymbol{W}_g\boldsymbol{\zeta})^{(1)}\end{aligned}$$

$$
\begin{aligned}
||\boldsymbol{u}||^{(2)} = ||\mathbf{y}||^2 + n(\alpha)^{(2)} + \sum_s ||X_s||^2(\beta_s)^{(2)} + \sum_g (\boldsymbol{\zeta}_g^T \boldsymbol{W}_g^T \boldsymbol{W}_g \boldsymbol{\zeta}_g)^{(1)} + \mathbb{E}_q[\boldsymbol{\theta}_\xi^T \boldsymbol{Z}_\xi^T \boldsymbol{Z}_\xi \boldsymbol{\theta}_\xi] \\
- 2\sum_s y^T X_s(\beta_s)^{(1)} - 2\mathbf{y}^T \boldsymbol{Z}(\boldsymbol{\theta}_\xi)^{(1)} - 2\sum_g \mathbf{y}^T \boldsymbol{W}_g(\boldsymbol{\zeta}_g)^{(1)} - 2(\alpha)^{(1)}\mathbf{1}_n^T\mathbf{y} + \\
+ 2\sum_{s\neq s', s<s'} X_s^T X_{s'}(\beta_s)^{(1)}(\beta_{s'})^{(1)} + 2(\boldsymbol{Z}(\boldsymbol{\theta})^{(1)})^T(\sum_s X_s(\beta_s)^{(1)}) + \\
+ 2(\boldsymbol{Z}(\boldsymbol{\theta})^{(1)})^T(\sum_g \boldsymbol{W}_g(\boldsymbol{\zeta}_g)^{(1)}) + 2\sum_{g\neq g', g<g'} (\boldsymbol{\zeta}_g)^{(1)T}\boldsymbol{W}_g^T \boldsymbol{W}_{g'}(\boldsymbol{\zeta}_{g'})^{(1)} + \\
+ 2(\alpha)^{(1)}\mathbf{1}_n^T \sum_s X_s(\beta_s)^{(1)} + 2(\alpha)^{(1)}\mathbf{1}_n^T \boldsymbol{Z}(\boldsymbol{\theta})^{(1)} + 2(\alpha)^{(1)}\mathbf{1}_n^T \sum_g \boldsymbol{W}_g(\boldsymbol{\zeta}_g)^{(1)} \\
+ 2\sum_s \sum_g (\beta_s)^{(1)} X_s^T \boldsymbol{W}_g(\boldsymbol{\zeta}_g)^{(1)}. \qquad (1.82)
\end{aligned}
$$

$$
\begin{aligned}
\log q(\sigma^2) =& \frac{n}{2}\log\sigma^{-2} - \frac{\sigma^{-2}}{2}\mathbb{E}_{-\sigma^2}\left[\|\mathbf{y} - \alpha\mathbf{1}_n - \sum_s X_s\gamma_s\beta_s - \boldsymbol{Z}_\xi\boldsymbol{\theta}_\xi - \sum_g \boldsymbol{W}_g\boldsymbol{\zeta}_g\|^2\right] + \\
&(\tau+1)\log\sigma^{-2} - (\nu)^{(1)}\sigma^{-2} + cst \\
=& \log\sigma^{-2}\left(\frac{n}{2} + \tau + 1\right) + \sigma^{-2}\left(\frac{\|\boldsymbol{u}\|^{(2)}}{2} + (\nu)^{(1)}\right) + cst
\end{aligned}
$$

$$
q(\sigma^2) = Inv - Gamma(\nu^*, \tau^*)
$$

$$
\nu^* = \frac{n}{2} + \tau \qquad (1.83)
$$

$$
\tau^* = \frac{\|\boldsymbol{u}\|^{(2)}}{2} + (\nu)^{(1)} \qquad (1.84)
$$

where

$$(\sigma^{-2})^{(1)} = \frac{\nu^*}{\tau^*} \tag{1.85}$$

$$(\log \sigma^{-2})^{(1)} = \Psi(\nu^*) - \log \tau^* \tag{1.86}$$

The update for $q(\kappa)$ is

$$\begin{aligned}
\log q(\kappa) &= \mathbb{E}_{-\kappa}\left[\log \prod_j p(\xi_j|\kappa) + \log p(\kappa)\right] + cst \\
&= \mathbb{E}_{-\kappa}\Big[\Big(\sum_j \xi_j \log(\kappa) + \sum_j (1-\xi_j)\log(1-\kappa)\Big)\mathrm{I}\Big[\sum_j \xi_j \neq 1\Big] + (a_j - 1)\log(\kappa) + \\
&+ (b_j - 1)\log(1-\kappa)\Big] + cst
\end{aligned}$$

As the update for ξ from the construction of the MCMC and the SMVN is

$$\mathbb{E}_q[\xi] = \mathbb{E}_q\left[\xi \ \mathrm{I}\Big[\sum_j \xi_j \neq 1\Big]\right] = (\xi)^{(1)} \tag{1.87}$$

the update can be solved in closed form, using the MCMC marginal expectations.

$$\log q(\kappa) = \left(\sum_j (\xi_j)^{(1)} + a_j - 1\right)\log(\kappa) + \left(d - \sum_j (\xi_j)^{(1)} + b_j - 1\right)\log(1-\kappa) + cst$$

$$q(\kappa) = Beta(a^*_\kappa, b^*_\kappa) \tag{1.88}$$

with parameters

$$a_\kappa^* = a_\kappa + \sum_j (\xi_j)^{(1)} \tag{1.89}$$

$$b_\kappa^* = b_\kappa + d - \sum_j (\xi_j)^{(1)} \tag{1.90}$$

where

$$\begin{aligned}
(\kappa)^{(1)} &= a_\kappa^* / \left(a_\kappa^* + b_\kappa^*\right) = a_\kappa^* / \left(a_\kappa + b_\kappa + d\right) && (1.91)\\
(\log \kappa)^{(1)} &= \Psi(a_\kappa^*) - \Psi(a_\kappa^* + b_\kappa^*)\\
(\log(1-\kappa))^{(1)} &= \Psi(b_\kappa^*) - \Psi(a_\kappa^* + b_\kappa^*)
\end{aligned}$$

where $\Psi(\cdot)$ is the digamma function.

The update for $q(\varrho)$ is

$$\begin{aligned}
\log q(\varrho) &= \mathbb{E}_{-\varrho}\left[\log p(\chi_g|\varrho) + \log p(\varrho)\right] + cst\\
&\propto \mathbb{E}_{-\varrho}\left[\chi_g \log(\varrho) + (1-\chi_g)\log(1-\varrho) + (a_\varrho - 1)\log(\varrho) + (b_\varrho - 1)\log(1-\varrho)\right]\\
&\propto ((\chi_g)^{(1)} + a_\varrho - 1)\log(\varrho) + (1 - (\chi_g)^{(1)} + b_\varrho - 1)\log(1-\varrho)
\end{aligned}$$

$$q(\varrho) = Beta(a_\varrho^*, b_\varrho^*) \tag{1.92}$$

with parameters

$$a_{\varrho}^{*} = a_{\varrho} + \sum_{g} (\chi_g)^{(1)} \tag{1.93}$$

$$b_{\varrho}^{*} = b_{\varrho} + G - \sum_{g} (\chi_g)^{(1)} \tag{1.94}$$

where

$$(\varrho)^{(1)} = a_{\varrho}^{*} / \left(a_{\varrho}^{*} + b_{\varrho}^{*}\right) = a_{\varrho}^{*} / \left(a_{\varrho} + b_{\varrho} + G\right) \tag{1.95}$$

$$(\log \varrho)^{(1)} = \Psi(a_{\varrho}^{*}) - \Psi(a_{\varrho}^{*} + b_{\varrho}^{*})$$

$$(\log(1-\varrho))^{(1)} = \Psi(b_{\varrho}^{*}) - \Psi(a_{\varrho}^{*} + b_{\varrho}^{*})$$

where $\Psi(\cdot)$ is the digamma function.

$$\begin{aligned}\log q(\nu) &= \mathbb{E}_{-\nu}\left[\log p(\sigma^2|\tau,\nu) + \log p(\nu)\right] + cst \\ &= \tau \log \nu - \nu(\sigma^{-2})^{(1)} + (a_\nu - 1)\log \nu - \nu b_\nu \\ &= (\tau + a_\nu - 1)\log \nu - ((\sigma^{-2})^{(1)} + b_\nu)\nu\end{aligned}$$

$$q(\nu) = Inv - Gamma(a_\nu^{*}, b_\nu^{*})$$

$$a_\nu^{*} = \tau + a_\nu \tag{1.96}$$

$$b_\nu^{*} = (\sigma^{-2})^{(1)} + b_\nu \tag{1.97}$$

where

$$(\nu)^{(1)} = \frac{a_\nu^*}{b_\nu^*} \tag{1.98}$$

$$(\log \nu)^{(1)} = \Psi(a_\nu^*) - \log b_\nu^* \tag{1.99}$$

1.3 Pseudo Updates

The pseudo updates are derived in full. The prior parametrisation is

$$p(\Omega_j|\Delta_j, \Upsilon_j) = \left[\frac{1}{(2\pi\Delta_j)^{(-1/2)}} \exp\left(-\frac{1}{2\Delta_j}\Omega_j^2\right)\right]^{\Upsilon_j} \delta_0(\Omega_j)^{1-\Upsilon_j} \tag{1.100}$$

$$p(\Delta_j|\Upsilon_j) = \left[\frac{b_\Delta^{a_\Delta}}{\Gamma(a_\Delta)}(\Delta_j)^{-a_\Delta-1} \exp\{-b_\Delta \Delta_j^{-1}\}\right]^{\Upsilon_j} \delta_0(\Delta_j)^{1-\Upsilon_j} \tag{1.101}$$

$$P(\Upsilon_j) = (\kappa)^{\Upsilon_j}(1-\kappa)^{1-\Upsilon_j} \tag{1.102}$$

The joint update $q(\Omega_j, \Upsilon_j)$ is

$$q(\Omega_j, \Upsilon_j) \propto \mathbb{E}_{(-\Omega_j,\Upsilon_j)}\Big[\log p(y|.) + \log p(\Omega_j|\Delta_j, \Upsilon_j) + p(\Delta_j|\Upsilon_j) + p(\Upsilon_j)\Big] \tag{1.103}$$

$$\begin{aligned} q(\Omega_j, \Upsilon_j) \propto & \Big[N(\Omega_j|\mu_{\Omega_j}, \sigma^2_{\Omega_j})\Big]^{\Upsilon_j} [\delta_0(\Omega_j)]^{1-\Upsilon_j} \\ & \Big[\exp\Big(\frac{1}{2}\log\sigma^2_{\Omega_j} + (\log\kappa)^{(1)} - \frac{1}{2}\mathbb{E}_q(\log\Delta_j|\Upsilon_j) + \frac{1}{2}\mu^2_{\Omega,j}\sigma^{-2}_{\Omega,j} + a_\Delta\log(b_\Delta) + \\ & - \log(\Gamma(a_\Delta)) - (a_\Delta+1)\mathbb{E}_q(\log\Delta_j|\Upsilon_j) - b_\Delta\mathbb{E}_q[\Delta_j^{-1}|\Upsilon_j]\Big)\Big]^{\Upsilon} \\ & \Big[(1-\kappa)^{(1)} + \delta_0(\Delta_j)\Big]^{1-\Upsilon_j} \end{aligned}$$

$$
\begin{aligned}
\sigma^2_{\Omega,j} &= \left[\|Z_j\|^2(\sigma^{-2})^{(1)} + \mathbb{E}_q[\Delta_j^{-1}|\Upsilon_j]\right]^{-1} \\
\mu_{\Omega,j} &= \sigma^2_{\Omega,j} Z_j^T\left[(\sigma^{-2})^{(1)}\left(y - (\alpha)^{(1)}\mathbf{1}_n - \sum_{k\neq j} Z_k(\Omega_k)^{(1)} - \sum_s X_s(\beta_s)^{(1)} - \sum_g W_g(\boldsymbol{\zeta}_g)^{(1)}\right)\right]
\end{aligned}
$$

$$
\pi(\Omega_j|\Upsilon_j = 1, y) = \mathcal{N}(\mu_{\Omega,j}, \sigma^2_{\Omega,j}), \quad q(\Omega_j|\Upsilon_j = 0, y) = \delta_0(\Omega_j) \tag{1.104}
$$

which gives us the update

$$
\begin{aligned}
\sigma^2_{\Omega,j} &= \left[\|Z_j\|^2(\sigma^{-2})^{(1)} + \mathbb{E}_q[\Delta_j^{-1}|\Upsilon_j = 1]\right]^{-1} \\
\mu_{\Omega,j} &= \sigma^2_{\Omega,j} Z_j^T\left[(\sigma^{-2})^{(1)}\left(\mathbf{y} - (\alpha)^{(1)}\mathbf{1}_n - \sum_{k\neq j} Z_k(\Omega_k)^{(1)} - \sum_s X_s(\beta_s)^{(1)} - \sum \boldsymbol{W}_g(\boldsymbol{\zeta}_g)^{(1)}\right)\right]
\end{aligned}
$$

The terms in the $q(\Upsilon_j)$, using $\Delta_j = 0$ when $\Upsilon_j = 0$, are proportional to

$$
\begin{aligned}
p(\Upsilon_j = 1) \propto \exp\Big(&\frac{1}{2}\log\sigma^2_{\Omega_j} + (\log\kappa)^{(1)} - (a_\Delta + 3/2)\mathbb{E}_q(\log\Delta_j|\Upsilon_j = 1) + \frac{1}{2}\mu^2_{\Omega,j}\sigma^{-2}_{\Omega,j} + \\
&+ a_\Delta\log(b_\Delta) - \log(\Gamma(a_\Delta)) - b_\Delta\mathbb{E}_q[\Delta_j^{-1}|\Upsilon_j = 1]\Big) \\
p(\Upsilon_j = 0) \propto& (\log(1-\kappa))^{(1)}
\end{aligned}
$$

Which after normalisation is

$$
\begin{aligned}
(\Upsilon_j)^{(1)} = \Bigg[1 + \exp\Bigg\{&\frac{1}{2}\log\left(\sigma^{-2}_{\Omega,s}\right) + (\log(1-\kappa))^{(1)} - (\log\kappa)^{(1)} + \frac{1}{2}\mathbb{E}_q(\log\Delta_j|\Upsilon_j = 1) + \\
&- \frac{1}{2}\mu^2_{\Omega,j}\sigma^{-2}_{\Omega,j} - a_\Delta\log(b_\Delta) + \log(\Gamma(a_\Delta)) + (a_\Delta + 1)\mathbb{E}_q(\log\Delta_j|\Upsilon_j = 1) + \\
&+ b_\Delta\mathbb{E}_q[\Delta_j^{-1}|\Upsilon_j = 1]\Bigg\}\Bigg]^{-1}
\end{aligned}
$$

Note that now

$$(\Omega_j)^{(1)} = \mu_{\Omega,j}(\Upsilon_j)^{(1)} \tag{1.105}$$

$$(\Omega_j)^{(2)} = (\sigma^2_{\Omega,j} + \mu^2_{\Omega,j})(\Upsilon_j)^{(1)}. \tag{1.106}$$

The approximating q density for Δ_j, which is proportional to Δ_j but conditional on Υ_j is

$$\begin{aligned}
\log q(\Delta_j|\Upsilon_j) &\propto \mathbb{E}_{q(-\Delta_j,-\Upsilon_j)}\Big[\log p(\Omega_j|\Upsilon_j,\Delta_j) + \log p(\Delta_j|\Upsilon_j)\Big] \\
&\propto \mathbb{E}_{q(-\Delta_j,-\Upsilon_j)}\Big[\frac{1}{2}\log \Delta_j^{-1}\Upsilon_j - \frac{1}{2}\Omega_j^2\Upsilon_j\Delta_j^{-1} + \Upsilon_j(a_\Delta+1)\log\Delta_j^{-1} + \\
&\quad - b_\Delta\Upsilon_j\Delta_j^{-1} + (1-\Upsilon_j)\delta_0(\Delta_j)\Big] \\
&\propto \mathbb{E}_{q(-\Delta_j,-\Upsilon_j)}\left[(\log\Delta_j^{-1})\Upsilon_j\left(\frac{1}{2}+a_\Delta+1\right) - \Delta_j^{-1}\Upsilon_j\left(\frac{1}{2}\Omega_j^2 + b_\Delta\right)\right]\Big[(1-\Upsilon_j)\delta_0(\Delta_j)\Big]
\end{aligned}$$

which gives us

$$q(\Delta_j|\Upsilon_j) \sim \Big[IG(\Delta_j|a^*_{\Delta_j}, b^*_{\Delta_j})\Big]^{\Upsilon_j}\Big[\delta_0(\Delta_j)\Big]^{(1-\Upsilon_j)} \tag{1.107}$$

Under q

$$q(\Delta_j|\Upsilon_j = 1, y) \sim IG(\Delta_j|a^*_{\Delta_j}, b^*_{\Delta_j}), \quad q(\Delta_j|\Upsilon_j = 0, y), \sim \delta_0(\Delta_j)$$

with updates

$$a^*_{\Delta,j} = \frac{1}{2} + a_\Delta \tag{1.108}$$

$$\begin{aligned}
b^*_{\Delta,j} &= \frac{1}{2}\mathbb{E}[\Omega_j^2|\Upsilon_j = 1] + b_\Delta \\
&= \frac{1}{2}(\sigma^2_{\Omega,j} + \mu^2_{\Omega,j}) + b_\Delta
\end{aligned} \tag{1.109}$$

This gives

$$\mathbb{E}_q(\Delta_j^{-1}|\Upsilon_j = 1) = a^*_{\Delta,j}/b^*_{\Delta,j} \tag{1.110}$$
$$\mathbb{E}_q(\log \Delta_j|\Upsilon_j = 1) = \log\left(b^*_{\Delta_j}\right) - \Psi(a^*_{\Delta_j})$$

The auxiliary parameters create an alternative DAG which is updated via a "separate branch" of pseudo updates which helps us to approximate the model in order to guide the MCMC step. These updates are refined at each iteration by the full VI updates which account for the constraint. The "sparsity" parameter κ and the hyperparameters a_Δ, b_Δ which are set to a_ψ, b_ψ provide a link back to the constrained model.

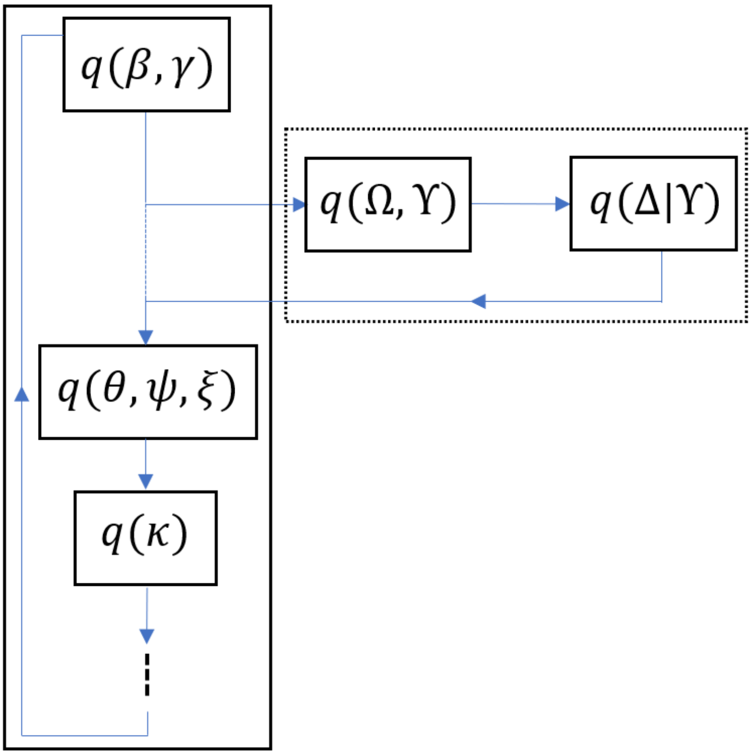

Figure S1: Diagram depicting the order and structure of the CAVI updates. Although the CAVI-MC permits any order, the pseudo updates for the auxiliary parameters help guide the MCMC and are performed directly before the $q(\boldsymbol{\theta}, \boldsymbol{\psi}, \boldsymbol{\xi})$ MC update. The pseudo updates for an unconstrained model are in the dashed box and branch off prior to the joint $q(\boldsymbol{\theta}, \boldsymbol{\psi}, \boldsymbol{\xi})$ update. The q approximating densities $q(\Delta_j|\Upsilon_j = 1)$ are then used to guide the MCMC step.

1.4 ELBO

The objective of VI is to find the candidate from a family of densities $\mathcal{D}$ which best approximates, the one closest in KL divergence, to exact conditional

$$q^*(\boldsymbol{\vartheta}) = \underset{q^*(\boldsymbol{\vartheta})\in\mathcal{D}}{\arg\min}\ \mathrm{KL}(q(\boldsymbol{\vartheta})||p(\boldsymbol{\vartheta}|y))$$

This objective is not computable as it requires computing marginal likelihood. If we expand the expression

$$\mathrm{KL}(q(\boldsymbol{\vartheta})||p(\boldsymbol{\vartheta}|\mathbf{y})) = \mathbb{E}_{q(\boldsymbol{\vartheta})}[\log q(\boldsymbol{\vartheta})] - \mathbb{E}_{q(\boldsymbol{\vartheta})}[\log p(\boldsymbol{\vartheta},\mathbf{y})] + \log p(\mathbf{y})$$

we can identify the elements which are a function of the parameters in the model. As the KL cannot be computed, an alternative objective that is equivalent to the KL up to an added constant is the evidence lower bound (ELBO).

$$\mathcal{L}(q) = \mathbb{E}_{q(\boldsymbol{\vartheta})}[\log p(\boldsymbol{\vartheta},\mathbf{y})] - \log q(\boldsymbol{\vartheta}) \tag{1.111}$$

This function is the negative KL divergence plus the marginal likelihood, and is optimised at each iteration of the CAVI in order to monitor its convergence. The computational details are:

$$\begin{aligned}\mathcal{L}(q) = {} & \mathbb{E}_{q(\boldsymbol{\vartheta})}[\log p(\mathbf{y},\boldsymbol{\vartheta})] - \mathbb{E}_{q(\boldsymbol{\vartheta})}[\log q(\boldsymbol{\vartheta})]\\ &= A(\mathbf{y}|\cdot) + B^*(\alpha|w_\alpha) + \sum_s B(\beta_s,\gamma_s|w,\omega) + \tilde{B}(\boldsymbol{\theta},\boldsymbol{\psi},\boldsymbol{\xi}|\kappa) + \sum_g \hat{B}(\boldsymbol{\zeta}_g,\chi_g|v,\varrho)+\\ &\quad + C(\omega) + \tilde{C}(\kappa) + \hat{C}(\varrho) + D(w) + D^*(w_\alpha) + \hat{D}(v)+\\ &\quad + F(\sigma^2|\tau,\nu) + G(\nu) + H(b_w) + H^*(b_\alpha) + \hat{H}(b_v).\end{aligned}$$

The functions are

$$
\begin{aligned}
A(\mathbf{y}|\boldsymbol{\beta},\boldsymbol{\theta},\boldsymbol{\zeta},\sigma^2) =&\mathbb{E}_q[\log p(\mathbf{y}|\beta,\theta,\zeta,\sigma^2)]\\
=&\mathbb{E}_q\left[-\frac{n}{2}\log(2\pi)+\frac{n}{2}\log\bigl(\sigma^{-2}\bigr)-\frac{1}{2\sigma^2}\left\|\boldsymbol{u}\right\|^2\right]\\
=&-\frac{n}{2}\log(2\pi)+\frac{n}{2}\log\bigl(\sigma^2\bigr)^{(1)}-\frac{(\sigma^{-2})^{(1)}\left\|\boldsymbol{u}\right\|^{(2)}}{2}
\end{aligned}
$$

where $||\boldsymbol{u}||^2$ is defined in (1.82).

$$
\begin{aligned}
B^*(\alpha|w_\alpha) =&\mathbb{E}_q[\log p(\alpha|w_\alpha)]-\mathbb{E}_q[\log q(\alpha)]\\
=&\biggl[-\frac{1}{2}\log(2\pi)+\frac{1}{2}(\log w_\alpha^{-1})^{(1)}-\frac{1}{2(w_\alpha)^{(1)}}(\alpha)^{(2)}\biggr]-\\
&\biggl[-\frac{1}{2}\log(2\pi)-\frac{1}{2}(\log\sigma_\alpha^2)-\frac{1}{2(\sigma_\alpha^2)}\mathbb{E}_q\left[(\alpha-\mu_\alpha)^2\right]\biggr]\\
=&\frac{1}{2}\log\bigl(\sigma_\alpha^2\bigr)+\frac{1}{2}(\log w_\alpha^{-1})^{(1)}+\frac{1}{2}-\frac{1}{2}(w_\alpha^{-1})^{(1)}(\alpha)^{(2)}
\end{aligned}
\tag{1.112}
$$

$$
\begin{aligned}
B(\beta_s,\gamma_s|w,\omega) =&\mathbb{E}_q[\log p(\beta_s|\gamma_s,w)]+\mathbb{E}_q[\log p(\gamma_s|\omega)]-\mathbb{E}_q[\log q(\beta_s,\gamma_s)]\\
=&(\gamma_s)^{(1)}\biggl[-\frac{1}{2}\log(2\pi)+\frac{1}{2}(\log w^{-1})^{(1)}\biggr]-\mathbb{E}_q\left[\frac{1}{2w}\gamma_s\beta_s^2\right]+\\
&+(1-(\gamma_s)^{(1)})\delta_0(\beta_s)+(\gamma_s)^{(1)}(\log\omega)^{(1)}+(1-(\gamma_s)^{(1)})(\log(1-\omega))^{(1)}+\\
&+\frac{1}{2}(\gamma_s)^{(1)}\left[\log(2\pi)+\log\sigma_{\beta,s}^2\right]+\mathbb{E}_q\left[\frac{1}{2\sigma_{\beta,s}^2}\gamma_s\Bigl(\beta_s^2-2\beta_s\mu_{\beta,s}+\mu_{\beta,s}^2\Bigr)\right]+\\
&-(1-(\gamma_s)^{(1)})\delta_0(\beta_s)-(\gamma_s)^{(1)}\log(\gamma_s)^{(1)}-(1-(\gamma_s)^{(1)})\log\bigl(1-(\gamma_s)^{(1)}\bigr)
\end{aligned}
\tag{1.113}
$$

Simplifying using $\mathbb{E}_q\Bigg[\frac{1}{2\sigma^2_{\beta,s}}\gamma_s\Big(\beta_s^2 - 2\beta_s\mu_{\beta,s} + \mu^2_{\beta,s}\Big)\Bigg] = -(\gamma_s)^{(1)}/2$

$$\begin{aligned} B(\beta_s, \gamma_s|w,\omega) =& \frac{(\gamma_s)^{(1)}}{2}\Bigg[(\log w^{-1})^{(1)} + 2(\log\omega)^{(1)} + 1 + \log\sigma^2_{\beta,s} + 1 - 2\log(\gamma_s)^{(1)}\Bigg] + \\ & - \frac{(\gamma_s)^{(1)}}{2}\Bigg[(\sigma^2_{\beta,s} + \mu^2_{\beta,s})(w)^{(-1)}\Bigg] + \\ & + (1-(\gamma_s)^{(1)})\Bigg[(\log(1-\omega))^{(1)} + \log\big(1-(\gamma_s)^{(1)}\big)\Bigg] \end{aligned} \tag{1.114}$$

$$\begin{aligned} \hat{B}(\boldsymbol{\zeta}_g, \chi_g|v,\varrho) &= \mathbb{E}_q[\log p(\boldsymbol{\zeta}_g|\chi_g, v)] + \mathbb{E}_q[\log p(\chi_g|\varrho)] - \mathbb{E}_q[\log q(\boldsymbol{\zeta}_g, \chi_g)] \\ =& (\chi_g)^{(1)}\Bigg[-\frac{m_g}{2}\log(2\pi) + \frac{m_g}{2}(\log v^{-1})^{(1)}\Bigg] - \mathbb{E}_q\Bigg[\frac{1}{2v}\chi_g\boldsymbol{\zeta}_g^T\boldsymbol{\zeta}_g\Bigg] + \\ & + (1-(\chi_g)^{(1)})\delta_0(\boldsymbol{\zeta}_g) + (\chi_g)^{(1)}(\log\varrho)^{(1)} + (1-(\chi_g)^{(1)})(\log(1-\varrho))^{(1)} + \\ & + \frac{1}{2}(\chi_g)^{(1)}\Bigg[m_g\log(2\pi) + \log\det(\Sigma_{\zeta_g})\Bigg] - (1-(\chi_g)^{(1)})\delta_0(\boldsymbol{\zeta}_g) - (\chi_g)^{(1)}\log(\chi_g)^{(1)} \\ & + \mathbb{E}_q\Bigg[\frac{1}{2}\chi_g(\boldsymbol{\zeta}_g - \boldsymbol{\mu}_{\zeta_g})^T\Sigma^{-1}_{\zeta_g}(\boldsymbol{\zeta}_g - \boldsymbol{\mu}_{\zeta_g})\Bigg] - (1-(\chi_g)^{(1)})\log\big(1-(\chi_g)^{(1)}\big) \end{aligned} \tag{1.115}$$

Simplifying using $\mathbb{E}_q\Bigg[\chi_g\left(\boldsymbol{\zeta}_g^T\Sigma^{-1}_{\zeta_g}\boldsymbol{\zeta}_g\right)\Bigg] = m_g(\chi_g)^{(1)}$

$$\begin{aligned} \hat{B}(\boldsymbol{\zeta}_g, \chi_g|v,\varrho) =& \frac{(\chi_g)^{(1)}}{2}\Bigg(m_g(\log v^{-1})^{(1)} - \frac{1}{(v)^{(1)}}(tr(\Sigma_{\zeta_g}) + \boldsymbol{\mu}^T_{\zeta_g}\boldsymbol{\mu}_{\zeta_g}) + \log\det(\Sigma_{\zeta_g}) + m_g + \\ & + 2(\log\varrho)^{(1)} - 2\log\big((\chi_g)^{(1)}\big)\Bigg) + \\ & + (1-(\chi_g)^{(1)})\Bigg(\log\big(1-(\chi_g)^{(1)}\big) + (\log(1-\varrho))^{(1)}\Bigg) \end{aligned} \tag{1.116}$$

$$\tilde{B}(\boldsymbol{\theta}, \boldsymbol{\xi}, \boldsymbol{\psi}|\cdot) = \mathbb{E}_{q(\boldsymbol{\vartheta})}\Big[\log p(\boldsymbol{\theta}|\boldsymbol{\psi}, \boldsymbol{\xi}) + \log p(\boldsymbol{\psi}|\boldsymbol{\xi}) + \log p(\boldsymbol{\xi})\Big] - \mathbb{E}_{\log q(\boldsymbol{\vartheta})}\Big[\log q(\boldsymbol{\theta}, \boldsymbol{\psi}, \boldsymbol{\xi})\Big] \tag{1.117}$$

The approximating density is only known up to a constant of proportionality but this is sufficient for the ELBO calculations.

$$\begin{aligned}
\mathbb{E}_{q(\boldsymbol{\vartheta})}\Big[\log(p(\boldsymbol{\theta}, \boldsymbol{\xi}, \boldsymbol{\psi}))\Big] &= -\frac{1}{2}((d_\xi)^{(1)} - 1)\log(2\pi) - \frac{1}{2}(\log(\det{}^*(\mathbf{T}_\xi D(\boldsymbol{\psi}_\xi)\mathbf{T}_\xi))^{(1)} + \\
&- \frac{1}{2}(\boldsymbol{\theta}_\xi^T(\mathbf{T}_\xi D(\boldsymbol{\psi}_\xi)\mathbf{T}_\xi)^+\theta_\xi)^{(1)} + \sum_j(\xi_j)^{(1)}(\log\kappa)^{(1)} + \sum_j(1 - (\xi_j)^{(1)})(\log\kappa)^{(1)} + \\
&- \sum_j(a_\psi + 1)(\xi_j\log(\psi_j))^{(1)} - b_\psi\sum_j(\xi_j\psi_j^{-1})^{(1)} + (a_\psi\log(b_\psi) - \log(\Gamma(a_\psi))\sum_j(\xi_j)^{(1)}
\end{aligned}$$

The q expectations $(\xi_j\log(\psi_j))^{(1)}$ and $(\xi_j\psi_j^{-1})^{(1)}$ can be found using the law of iterative expectations but these will cancel. The free parameters are a function of ξ so when we take an expectation we have

$$\begin{aligned}
\mathbb{E}_{q(\vartheta)}\Big[\log q(\boldsymbol{\theta}, \boldsymbol{\xi}, \boldsymbol{\psi}|\mathbf{y})\Big] &\propto \mathbb{E}_{q(\boldsymbol{\vartheta})}\Big[\log(\text{SMVN}(\boldsymbol{\theta}))\Big] + \frac{1}{2}(\boldsymbol{\mu}_{\theta_\xi}^T\mathbf{T}_\xi(\mathbf{T}_\xi^T\Sigma_{\theta_\xi}\mathbf{T}_\xi)^+\mathbf{T}_\xi\boldsymbol{\mu}_{\theta_\xi})^{(1)} + \\
&+ \frac{1}{2}(\log\big(\det{}^*(\mathbf{T}_\xi\Sigma_{\theta_\xi}\mathbf{T}_\xi)\big))^{(1)} - \frac{1}{2}(\log(\det{}^*(\mathbf{T}_\xi D(\boldsymbol{\psi}_\xi)\mathbf{T}_\xi)))^{(1)} + \\
&+ \sum_j\xi_j(\log\kappa)^{(1)} + \sum_j(1 - \xi_j)(\log(1 - \kappa))^{(1)} - \sum_j(a_\psi + 1)(\xi_j\log(\psi_j))^{(1)} + \\
&+ (a_\psi\log(b_\psi) - \log(\Gamma(a_\psi))\sum_j(\xi_j)^{(1)} - b_\psi\sum_j(\xi_j\psi_j^{-1})^{(1)}
\end{aligned} \tag{1.118}$$

$$
\begin{aligned}
\mathbb{E}_{q(\boldsymbol{\vartheta})}\Big[\log(\mathrm{SMVN}(\boldsymbol{\theta}))\Big] =& -\frac{1}{2}((d_\xi)^{(1)}-1)\log(2\pi)-\frac{1}{2}(\log(\det{}^*(\mathbf{T}_\xi\Sigma_\xi\mathbf{T}_\xi)))^{(1)}+\\
&-\frac{1}{2}\Bigg\{(\boldsymbol{\theta}_\xi^T(\mathbf{T}_\xi\Sigma_\xi\mathbf{T}_\xi)^+\boldsymbol{\theta}_\xi)^{(1)}-2(\boldsymbol{\theta}_\xi^T(\mathbf{T}_\xi\Sigma_\xi\mathbf{T}_\xi)^+\mathbf{T}_\xi\boldsymbol{\mu}_{\theta_\xi})^{(1)}+\\
&+(\boldsymbol{\mu}_{\theta_\xi}^T\mathbf{T}_\xi(\mathbf{T}_\xi\Sigma_\xi\mathbf{T}_\xi)^+\mathbf{T}_\xi\boldsymbol{\mu}_{\theta_\xi})^{(1)}\Bigg\}
\end{aligned}
\tag{1.119}
$$

Bringing together the expression for $\tilde{B}$

$$
\begin{aligned}
\tilde{B}(\boldsymbol{\theta},\boldsymbol{\psi},\boldsymbol{\xi}|\cdot) =&\mathbb{E}_{q(\boldsymbol{\vartheta})}\Big[\log p(\boldsymbol{\theta}|\boldsymbol{\xi},\boldsymbol{\psi})+\log p(\boldsymbol{\psi}|\boldsymbol{\xi},a_\psi,b_\psi)+\log p(\boldsymbol{\xi}|\kappa)\Big]-\mathbb{E}_{q(\boldsymbol{\vartheta})}\Big[\log q(\boldsymbol{\theta},\boldsymbol{\xi})\Big]\\
=&-\frac{1}{2}(\log(\det{}^*(\mathbf{T}_\xi D(\boldsymbol{\psi}_\xi)\mathbf{T}_\xi)))^{(1)}+\frac{1}{2}(\log(\det{}^*(\mathbf{T}_\xi\Sigma_\xi\mathbf{T}_\xi)))^{(1)}+\\
&-\frac{1}{2}\Big\{(\boldsymbol{\theta}_\xi^T(\mathbf{T}_\xi D(\boldsymbol{\psi}_\xi)\mathbf{T}_\xi)^+\boldsymbol{\theta}_\xi)^{(1)}-(\boldsymbol{\theta}_\xi^T(\mathbf{T}_\xi\Sigma_\xi\mathbf{T}_\xi)^+\boldsymbol{\theta}_\xi)^{(1)}\Big\}+\\
&+(\boldsymbol{\theta}_\xi^T(\mathbf{T}_\xi\Sigma_\xi\mathbf{T}_\xi)^+\mathbf{T}_\xi\boldsymbol{\mu}_{\theta_\xi})^{(1)}
\end{aligned}
\tag{1.120}
$$

$$
\begin{aligned}
\tilde{C}(\kappa) =&\mathbb{E}_q[\log p(\kappa)]-\mathbb{E}_q[\log q(\kappa)]\\
=&\log B(a_\kappa^*,b_\kappa^*)-\log B(a_\kappa,b_\kappa)+(a_\kappa^*-a_\kappa)(\log\kappa)^{(1)}+(b_\kappa^*-b_\kappa)(\log[1-\kappa])^{(1)}
\end{aligned}
\tag{1.121}
$$

$$
\begin{aligned}
C(\omega) =&\mathbb{E}_q[\log p(\omega)]-\mathbb{E}_q[\log q(\omega)]\\
=&\log B(a_\omega^*,b_\omega^*)-\log B(a_\omega,b_\omega)+\\
&+(a_\omega^*-a_\omega)(\log\omega)^{(1)}+(b_\omega^*-b_\omega)(\log(1-\omega))^{(1)}
\end{aligned}
\tag{1.122}
$$

$$
\begin{aligned}
\hat{C}(\varrho) =& \mathbb{E}_q[\log p(\varrho)] - \mathbb{E}_q[\log q(\varrho)] \\
=& \log B(a_\varrho^*, b_\varrho^*) - \log B(a_\varrho, b_\varrho) + (a_\varrho^* - a_\varrho)(\log \varrho)^{(1)} + (b_\varrho^* - b_\varrho)(\log[1-\varrho])^{(1)}
\end{aligned}
\tag{1.123}
$$

$$
\begin{aligned}
D(w) =& \mathbb{E}_q[\log p(w)] - \mathbb{E}_q[\log q(w)] \\
=& \mathbb{E}_q\Bigg[a_w \log b_w - \log \Gamma(a_w) + (a_w + 1) \log w^{-1} - b_w w^{-1}\Bigg] + \\
& - \mathbb{E}_q\Bigg[a_w^* \log b_w^* - \log \Gamma(a_w^*) - (a_w^* + 1) \log w^{-1} + b_w^* w^{-1}\Bigg] \\
=& a_w (\log b_w)^{(1)} - a_w^* \log b_w^* - \log \Gamma(a_w) + \log \Gamma(a_w^*) + \\
& + (a_w - a_w^*)(\log w^{-1})^{(1)} + (b_w^* - (b_w)^{(1)})(w^{-1})^{(1)}
\end{aligned}
\tag{1.124}
$$

$$
\begin{aligned}
D^*(w_\alpha) =& \mathbb{E}_q[\log p(w_\alpha)] - \mathbb{E}_q[\log q(w_\alpha)] \\
=& \mathbb{E}_q\Bigg[a_\alpha \log b_\alpha - \log \Gamma(a_\alpha) + (a_\alpha + 1) \log w_\alpha^{-1} - b_\alpha w_\alpha^{-1}\Bigg] + \\
& - \mathbb{E}_q\Bigg[a_\alpha^* \log b_\alpha^* - \log \Gamma(a_\alpha^*) - (a_\alpha^* + 1) \log w_\alpha^{-1} + b_\alpha^* w_\alpha^{-1}\Bigg] \\
=& a_\alpha (\log b_\alpha)^{(1)} - a_\alpha^* \log b_\alpha^* - \log \Gamma(a_\alpha) + \log \Gamma(a_\alpha^*) + \\
& + (a_\alpha - a_\alpha^*)(\log w_\alpha^{-1})^{(1)} + (b_\alpha^* - (b_\alpha)^{(1)})(w_\alpha^{-1})^{(1)}
\end{aligned}
\tag{1.125}
$$

$$\begin{aligned}\hat{D}(v) =&\mathbb{E}_q[\log p(v)] - \mathbb{E}_q[\log q(v)]\\ =&\mathbb{E}_q\left[a_v \log b_v - \log\Gamma(a_v) + (a_v+1)\log v^{-1} - b_v v^{-1}\right]+\\ &-\mathbb{E}_q\left[a_v^* \log b_v^* - \log\Gamma(a_v^*) - (a_v^*+1)\log v^{-1} + b_v^* v^{-1}\right]\\ =&a_v(\log b_v)^{(1)} - a_v^*\log b_v^* - \log\Gamma(a_v) + \log\Gamma(a_v^*)+\\ &+(a_v - a_v^*)(\log v^{-1})^{(1)} + (b_v^* - (b_v)^{(1)})(v^{-1})^{(1)} \end{aligned} \tag{1.126}$$

$$\begin{aligned}F(\sigma^2|\tau,\nu) =&\mathbb{E}_q[\log p(\sigma^2|\tau,\nu)] - \mathbb{E}_q[\log q(\sigma^2)]\\ =&\left[\tau(\log\nu)^{(1)} - \log\Gamma(\tau) + (\tau+1)(\log\sigma^{-2})^{(1)} - (\nu)^{(1)}(\sigma^{-2})^{(1)}\right]+\\ &-\left[\tau^*(\log\nu^*) - \log\Gamma(\tau^*) + (\tau^*+1)(\log\sigma^{-2})^{(1)} + \nu^*(\sigma^{-2})^{(1)}\right]\\ =&\log\Gamma(\tau^*) - \log\Gamma(\tau) + (\tau-\tau^*)(\log\sigma^{-2})^{(1)} + \tau(\log\nu)^{(1)}+\\ &-\tau^*(\log\nu^*) + (\sigma^{-2})^{(1)}(\nu^* - (\nu)^{(1)}) \end{aligned} \tag{1.127}$$

$$\begin{aligned}G(\nu) =&\mathbb{E}_q[\log p(\nu)] - \mathbb{E}_q[\log q(\nu)]\\ =&a_\nu\log b_\nu - a_\nu^*\log b_\nu^* + \log\Gamma(a_\nu^*) - \log\Gamma(a_\nu)+\\ &+(a_\nu - a_\nu^*)(\log\nu)^{(1)} + (b_\nu - b_\nu^*)(\nu)^{(1)}. \end{aligned} \tag{1.128}$$

$$
\begin{aligned}
H(b_w) =& \mathbb{E}_q[\log p(b_w)] - \mathbb{E}_q[\log q(b_w)] \\
=& \mathbb{E}_q\Bigg[a_b \log b_b - \log\Gamma(a_b) + (a_b - 1)\log b_w - b_b b_w\Bigg] + \\
& \mathbb{E}_q\Bigg[a_b^* \log b_b^* - \log\Gamma(a_b^*) + (a_b^* - 1)\log b_w - b_b^* b_w\Bigg] \\
=& a_b \log b_b - a_b^* \log b_b^* - \log\Gamma(a_b) + \log\Gamma(a_b^*) + (\log b_w)^{(1)}(a_b - a_b^*) + \\
& + (b_w)^{(1)}(b_b^* - b_b)
\end{aligned} \tag{1.129}
$$

$$
\begin{aligned}
H^*(b_\alpha) =& \mathbb{E}_q[\log p(b_\alpha)] - \mathbb{E}_q[\log q(b_\alpha)] \\
=& \mathbb{E}_q\Bigg[a_{b,\alpha} \log b_{b,\alpha} - \log\Gamma(a_{b,\alpha}) + (a_{b,\alpha} - 1)\log b_\alpha - b_\alpha b_{b,\alpha}\Bigg] + \\
& \mathbb{E}_q\Bigg[a_{b,\alpha}^* \log b_\alpha^* - \log\Gamma(a_{b,\alpha}^*) + (a_{b,\alpha}^* - 1)\log b_\alpha - b_\alpha^* b_{b,\alpha}\Bigg] \\
=& a_{b,\alpha} \log b_{b,\alpha} - a_{b,\alpha}^* \log b_\alpha^* - \log\Gamma(a_{b,\alpha}) + \log\Gamma(a_{b,\alpha}^*) + (\log b_\alpha)^{(1)}(a_{b,\alpha} - a_{b,\alpha}^*) + \\
& + (b_\alpha)^{(1)}(b_{b,\alpha}^* - b_{b,\alpha})
\end{aligned} \tag{1.130}
$$

$$
\begin{aligned}
\hat{H}(b_v) =& \mathbb{E}_q[\log p(b_v)] - \mathbb{E}_q[\log q(b_v)] \\
=& \mathbb{E}_q\Bigg[a_{bv} \log b_{bv} - \log\Gamma(a_{bv}) + (a_{bv} - 1)\log b_v - b_{bv} b_v\Bigg] + \\
& \mathbb{E}_q\Bigg[a_{bv}^* \log b_{bv}^* - \log\Gamma(a_{bv}^*) + (a_{bv}^* - 1)\log b_v - b_{bv}^* b_v\Bigg] \\
=& a_{bv} \log b_{bv} - a_{bv}^* \log b_{bv}^* - \log\Gamma(a_{bv}) + \log\Gamma(a_{bv}^*) + (\log b_v)^{(1)}(a_{bv} - a_{bv}^*) + \\
& + (b_v)^{(1)}(b_{bv}^* - b_{bv})
\end{aligned} \tag{1.131}
$$

2 RJMCMC moves and model proposals

This section explains the RJMCMC moves in detail. In the RJMCMC the proposal for $\psi_j|\xi_j = 1$ is from the q approximating density of the auxiliary parameter Ω_j, where the free parameters are obtained from the pseudo updates. As $q(\boldsymbol{\theta}|\boldsymbol{\psi}, \boldsymbol{\xi})$ is available in closed form, we are able to sample directly from it. Since the proposals do not depend on their current values, this leads to a reverse move which is a random function and thus a Jacobian which is equal to 1.

The RJMCMC involves the following steps:

- Select a birth-death or swap move with probability ϕ, $1-\phi$.
- Propose a new model $\boldsymbol{\xi}'$ with probability $j(\boldsymbol{\xi}, \boldsymbol{\xi}')$.
- Generate $\mathbf{u}$ from our proposal density $g(\mathbf{u}|\cdot) \sim q(\boldsymbol{\theta}'|\boldsymbol{\psi}', \boldsymbol{\xi}') \prod_j \pi(\boldsymbol{\psi}_j'|a^*_{\Delta_j}, b^*_{\Delta_j}, \boldsymbol{\xi}')$.
- Set $(\boldsymbol{\theta}'_{(\xi',\psi')}, \boldsymbol{\psi}'_{\xi'}, \mathbf{u}') = h(\boldsymbol{\theta}_{(\xi,\psi)}, \boldsymbol{\psi}_{\xi}, \mathbf{u})$ where h is a specified invertible mapping function.
- Accept the proposed move to model $\boldsymbol{\xi}'$ with probability

$$\alpha_b = \min\left\{1, \frac{\Big[q(\boldsymbol{\theta}'|\mathbf{y}, \boldsymbol{\xi}', \boldsymbol{\psi}')q(\boldsymbol{\psi}', \boldsymbol{\xi}'|\mathbf{y})\Big] j_m(\boldsymbol{\xi}', \boldsymbol{\xi}) g'(\mathbf{u}'|\cdot)}{\Big[q(\boldsymbol{\theta}|\mathbf{y}, \boldsymbol{\xi}, \boldsymbol{\psi})q(\boldsymbol{\psi}, \boldsymbol{\xi}|\mathbf{y})\Big] j_m(\boldsymbol{\xi}, \boldsymbol{\xi}') g(\mathbf{u}|\cdot)} \left|\frac{\partial h(\boldsymbol{\theta}_{(\xi,\psi)}, \boldsymbol{\psi}_{\xi}, \mathbf{u})}{\partial(\boldsymbol{\theta}_{(\xi,\psi)}, \boldsymbol{\psi}_{\xi}, \mathbf{u})}\right|\right\}.$$

  where the target is in the square parenthesis.

The acceptance probability for the RJMCMC between-model move, as the Jacobian is equal to 1, simplifies to

$$\alpha_b = \min\left\{1, \frac{q(\boldsymbol{\xi}', \boldsymbol{\psi}'|\mathbf{y}) j_m(\boldsymbol{\xi}', \boldsymbol{\xi}) \pi(\boldsymbol{\psi}|\boldsymbol{\xi})}{q(\boldsymbol{\xi}, \boldsymbol{\psi}|\mathbf{y}) j_m(\boldsymbol{\xi}, \boldsymbol{\xi}') \pi(\boldsymbol{\psi}'|\boldsymbol{\xi}')}\right\} \tag{2.1}$$

where $j_m(\boldsymbol{\xi}, \boldsymbol{\xi}')$ is the proposal probability for the latent variable selection parameter $\boldsymbol{\xi}'$ (which depends on the move type and the data) and

$$\begin{aligned}\log q(\boldsymbol{\psi}, \boldsymbol{\xi}|\mathbf{y}, .) \propto \frac{1}{2}\boldsymbol{\mu}^T_{\theta_{(\xi,\psi)}}\mathbf{T}_\xi(\mathbf{T}^T_\xi\Sigma_{\theta_{(\xi,\psi)}}\mathbf{T}_\xi)^{+}\mathbf{T}_\xi\boldsymbol{\mu}_{\theta_{(\xi,\psi)}} + \frac{1}{2}\log\Big(\det{}^*(\mathbf{T}_\xi\Sigma_{\theta_{(\xi,\psi)}}\mathbf{T}_\xi)\Big)+ \\ -\frac{1}{2}\log(\det{}^*(\mathbf{T}_\xi D(\boldsymbol{\psi}_\xi)\mathbf{T}_\xi)) + \sum_j \xi_j(\log\kappa)^{(1)} + \sum_j(1-\xi_j)(\log(1-\kappa))^{(1)}+ \\ -(a_\psi+1)\sum_j \xi_j\log(\psi_j) - b_\psi\sum_j \xi_j\psi_j^{-1} + (a_\psi\log(b_\psi) - \log(\Gamma(a_\psi))\sum_j \xi_j. \quad (2.2)\end{aligned}$$

As described in the main paper, a univariate approximation is used to calculate $j(\boldsymbol{\xi}, \boldsymbol{\xi}')$ in the birth-death or swap move of the RJMCMC.

2.1 Birth-death and swap moves

To guide the RJMCMC over a large binary space, we use a univariate approximation $\tilde{p}(\xi_j = 1|\boldsymbol{\vartheta})$ of the joint approximating density $q(\boldsymbol{\psi}, \boldsymbol{\xi})$ relative to the jth element. The probability of a new model $j_m(\boldsymbol{\xi}, \boldsymbol{\xi}')$ is a function of this approximation and the move type.

Each time a variable is selected for (or removed from) the model, the remaining approximate probabilities proposal for all elements outside of the model must be renormalised. The normalised probabilities for a variable h to be selected for the model, the birth move is

$$b_h(\boldsymbol{\vartheta}) = \frac{\tilde{p}_h(\xi_h = 1|\boldsymbol{\vartheta})}{\sum_{j\notin\mathcal{M}}\tilde{p}_j(\xi_j = 1|\boldsymbol{\vartheta})}, \quad (2.3)$$

where any $\tilde{p}(\xi_j = 1|\boldsymbol{\vartheta})$ below a small threshold ε_b (set at 1×10^{-30}) is replaced by ε_b to avoid zero probabilities. The normalised probabilities to remove a variable h from the

model $\mathcal{M}$, the death move is

$$d_h(\boldsymbol{\vartheta}) = \frac{1 - \tilde{p}_h(\xi_h = 1|\boldsymbol{\vartheta}) + \varepsilon_d}{\sum_{j \in \mathcal{M}}(1 - \tilde{p}_j(\xi_j = 1|\boldsymbol{\vartheta}) + \varepsilon_d)} \tag{2.4}$$

as we select the variables to remove with probability inversely proportional to the approximate probability of inclusion. ε_d guarantees that the probabilities are comparable when they are close to the limit of their domain. The difference between the groups is relative to the size of ε_d.

If i is the current iteration, define $\sum_j (\xi_j)^{[i]} = (d_\xi)^{[i]}$ the size of the current model in the MCMC, the proposal is generated in the following way:

Sample (birth-death) and swap with probability ϕ and $1-\phi$ respectively if $2 \leq (d_\xi)^{[i]} < d$:

- (Birth-Death) Sample uniformly birth or death:
  - (Birth): If $(d_\xi)^{[i]} = 0$ add 2 variables else add 1.

$$(d_\xi)^{[i]} \neq 0 \text{ (Birth)}: \quad \frac{j_m(\boldsymbol{\xi}', \boldsymbol{\xi})}{j_m(\boldsymbol{\xi}, \boldsymbol{\xi}')} = \frac{\phi(0.5)d(\boldsymbol{\vartheta})}{\phi(0.5)b(\boldsymbol{\vartheta})} \tag{2.5}$$

$$(d_\xi)^{[i]} = 0 \text{ (Birth)}: \quad \frac{j_m(\boldsymbol{\xi}', \boldsymbol{\xi})}{j_m(\boldsymbol{\xi}, \boldsymbol{\xi}')} = \frac{\phi(0.5)}{\phi(0.5)(b_{(h)}(\boldsymbol{\vartheta})b_{(l)}(\boldsymbol{\vartheta}) + b_{(l)}(\boldsymbol{\vartheta})b_{(h)}(\boldsymbol{\vartheta}))} \tag{2.6}$$

  - (Death): If $d_\xi = 2$ remove 2 variables else remove 1.

$$(d_\xi)^{[i]} = 2 \text{ (Death)}: \quad \frac{j_m(\boldsymbol{\xi}', \boldsymbol{\xi})}{j_m(\boldsymbol{\xi}, \boldsymbol{\xi}')} = \frac{\phi(0.5)(b_{(h)}(\boldsymbol{\vartheta})b_{(l)}(\boldsymbol{\vartheta}) + b_{(l)}(\boldsymbol{\vartheta})b_{(h)}(\boldsymbol{\vartheta}))}{\phi(0.5)} \tag{2.7}$$

$$(d_\xi)^{[i]} \notin \{0, 2\} \text{ (Death)}: \quad \frac{j_m(\boldsymbol{\xi}', \boldsymbol{\xi})}{j_m(\boldsymbol{\xi}, \boldsymbol{\xi}')} = \frac{\phi(0.5)b(\boldsymbol{\vartheta})}{\phi(0.5)d(\boldsymbol{\vartheta})} \tag{2.8}$$

When we add two elements h and l the order is not important. As the probability of

selecting each element is not the same, we have to add the probabilities so that

$$b_{(h)}(\boldsymbol{\vartheta})b_{(l)}(\boldsymbol{\vartheta}) + b_{(l)}(\boldsymbol{\vartheta})b_{(h)}(\boldsymbol{\vartheta}) \tag{2.9}$$

is the probability of choosing element h first and element l second plus the probability of choosing element l first and element h second (the order is in the bracket).

- (Swap):
  - Sample a variable included in the model h and swap with one outside l.

$$\text{(Swap)}: \quad \frac{j_m(\boldsymbol{\xi}', \boldsymbol{\xi})}{j_m(\boldsymbol{\xi}, \boldsymbol{\xi}')} = \frac{(1-\phi)d_l(\boldsymbol{\vartheta})b_h(\boldsymbol{\vartheta})}{(1-\phi)d_h(\boldsymbol{\vartheta})b_l(\boldsymbol{\vartheta})}. \tag{2.10}$$

2.2 Within-model moves

Within-model samples are included so that both $\boldsymbol{\psi}$ and $\boldsymbol{\theta}$ are sampled sufficiently. This enables the calculation of q expectations within the ELBO and the free parameter updates for $q(\sigma^2)$. Its is particularly important when estimating $||u||^{(2)}$ as the calculation has to be split into its component parts, because the latent variables which perform variable selection need to be incorporated for the expectations. If $\boldsymbol{\theta}|\boldsymbol{\xi}, \boldsymbol{\psi}$ has not been sampled sufficiently to estimate $\mathbb{E}_q[\boldsymbol{\theta}_\xi^T \boldsymbol{Z}_\xi^T \boldsymbol{Z}_\xi \boldsymbol{\theta}_\xi]$, then the cross product terms may not be sufficiently large enough to prevent the dot product from having a negative value.

The within-model move is performed after a successful between-model move and for a random subset of the total number of iterations. Conditional on $\boldsymbol{\xi}$, propose ψ_j for each j element in the model

$$\pi(\psi_j|\xi_j = 1) = IG(\psi_j|a^*_{\Delta_j}, b^*_{\Delta_j}) \tag{2.11}$$

and then propose the vector θ directly from the target distribution

$$\pi(\boldsymbol{\theta}_\xi|\boldsymbol{\xi}, \psi) = SMVN_{d_\xi}(\boldsymbol{\theta}_\xi|\boldsymbol{\mu}_{\theta_{(\xi,\psi)}}, \Sigma_{\theta_{(\xi,\psi)}}). \tag{2.12}$$

The acceptance probability simplifies to

$$\alpha_w = \min\left\{1, \frac{q(\boldsymbol{\psi}'|\mathbf{y}, \boldsymbol{\xi})\pi(\boldsymbol{\psi}|\boldsymbol{\xi})}{q(\boldsymbol{\psi}|\mathbf{y}, \boldsymbol{\xi})\pi(\boldsymbol{\psi}'|\boldsymbol{\xi})}\right\} \tag{2.13}$$

where $\log q(\boldsymbol{\psi}|\boldsymbol{\xi}, \mathbf{y})$ is proportional to (2.2).

3 Proofs

Here are some simple proofs of the results used in the derivations.

3.1 Proof: Simplification of the constraint matrix

We can simplify the calculations. $\mathbf{T}\mathbf{T}^T = \mathbf{T}\mathbf{T} = \mathbf{T}$. If we define the matrix

$$\mathbf{T} = \begin{pmatrix} 1-1/d & -1/d & \dots & -1/d \\ -1/d & 1-1/d & \ddots & \vdots \\ \vdots & \ddots & \ddots & -1/d \\ -1/d & \dots & -1/d & 1-1/d \end{pmatrix}$$

Then for the diagonal component of **TT** we either have entries corresponding to the dot product of

$$\begin{bmatrix} 1-1/d \\ -1/d \\ \vdots \\ -1/d \end{bmatrix} \cdot \begin{bmatrix} 1-1/d \\ -1/d \\ \vdots \\ -1/d \end{bmatrix} = (1-1/d)^2 + \frac{d-1}{d^2} = 1 - 1/d \tag{3.1}$$

where $1 - 1/d$ is in the same position in the vector. The off-diagonal entries correspond to dot product of vectors where the position of the $1 - 1/d$ terms are not matched which always gives us

$$(1 - 1/d) \times (-2/d) + (d-2)/d^2 = -1/d \, \square \tag{3.2}$$

Using the matrix determinant lemma where A is an invertible square matrix and u, v are column vectors

$$\det\left(A + uv^T\right) = (1 + v^T A^{-1} u) \det(A) \tag{3.3}$$

we can prove that the determinant of this matrix is zero. Express **T** as

$$\begin{aligned} \mathbf{T} &= (\mathbf{I}_d - (1/d)\mathbf{1}_{d\times d}) \\ &= \mathbf{I}_d + \begin{bmatrix} -1/\sqrt{d} \\ \vdots \\ -1/\sqrt{d} \end{bmatrix} \begin{bmatrix} 1/\sqrt{d} & \ldots & 1/\sqrt{d} \end{bmatrix} \end{aligned}$$

Thus

$$\det(\mathbf{T}) = 1 + \begin{bmatrix} 1/\sqrt{d} & \ldots & -1/\sqrt{d} \end{bmatrix} \begin{bmatrix} -1/\sqrt{d} \\ \vdots \\ 1/\sqrt{d} \end{bmatrix} \tag{3.4}$$

$$= 1 - 1 = 0 \, \square \tag{3.5}$$

3.2 Proof: Eigenvalues of T comprise of $d-1$ 1's and one 0.

To find the eigenvalues of $\mathbf{T}$ need to solve

$$\det(\mathbf{T} - \lambda\mathbf{I}) = 0 \tag{3.6}$$

for λ. Using the lemma in Equation (3.3) and $\mathbf{T} - \lambda\mathbf{I} = A + uv^T$ where

$$A = \text{diag}(1-\lambda) \qquad u = \begin{bmatrix} -1/\sqrt{d} \\ \vdots \\ -1/\sqrt{d} \end{bmatrix} \qquad v = \begin{bmatrix} 1/\sqrt{d} \\ \vdots \\ 1/\sqrt{d} \end{bmatrix} \tag{3.7}$$

we have

$$\begin{aligned} \det(\mathbf{T} - \lambda\mathbf{I}) &= (1 + v^T \text{diag}((1-\lambda)^{-1}))u)(1-\lambda)^d \\ &= (1 - (1-\lambda)^{-1})(1-\lambda)^d \\ &= \left(\frac{1-\lambda+1}{1-\lambda}\right)(1-\lambda)^d \\ &= -\lambda(1-\lambda)^{d-1}. \end{aligned} \tag{3.8}$$

Therefore the eigenvalues for $\mathbf{T}$ are

$$\lambda_1, \lambda_2, ..., \lambda_{d-1} = 1 \qquad \lambda_d = 0 \; \square \tag{3.9}$$

3.3 Proof: $\mathbf{T} = \mathbf{T}^+$

Using the SVD $\mathbf{T}$ can be expressed as $U\Lambda V$. As $\mathbf{T}$ is symmetric $U\Lambda V = U\Lambda U$. The pseudo inverse is

$$\mathbf{T}^+ = U\Lambda^+U^T = \begin{bmatrix} \\ u_1 & \cdots & u_d \\ \\ \end{bmatrix} \begin{bmatrix} \lambda_1^{-1} & & & \\ & \ddots & & \\ & & \lambda_{d-1}^{-1} & \\ & & & 0 \end{bmatrix} \begin{bmatrix} \\ u_1 & \cdots & u_d \\ \\ \end{bmatrix}$$

$$\begin{bmatrix} \\ u_1 & \cdots & u_d \\ \\ \end{bmatrix} \begin{bmatrix} 1 & & & \\ & \ddots & & \\ & & 1 & \\ & & & 0 \end{bmatrix} \begin{bmatrix} \\ u_1 & \cdots & u_d \\ \\ \end{bmatrix}$$

$$= \mathbf{T} \ \square$$

This approach can also be used to solve the pseudo determinant $\det^*(\theta\mathbf{T})$ (where θ is a scalar) which is a product of the non-zero eigenvalues. The eigenvalues of the scaled matrix can be found solving $\det(\theta\mathbf{T} - \lambda I) = 0$.

$$\det(\theta\mathbf{T} - \lambda\mathbf{I}) = (1 + v^T A^{-1} u) \det(A) \tag{3.10}$$

where

$$A = \mathrm{diag}(\theta - \lambda) \qquad u = \begin{bmatrix} -\sqrt{\theta/d} \\ \vdots \\ -\sqrt{\theta/d} \end{bmatrix} \qquad v = \begin{bmatrix} \sqrt{\theta/d} \\ \vdots \\ \sqrt{\theta/d} \end{bmatrix} \tag{3.11}$$

Simplifying gives

$$\det(\theta\mathbf{T} - \lambda\mathbf{I}) = (1 + v^T A^{-1} u) \det(A) \tag{3.12}$$

$$= -\lambda(\theta - \lambda)^{d-1} \tag{3.13}$$

The eigenvalues, found by setting this expression to zero are

$$\lambda_1, \lambda_2, ..., \lambda_{d-1} = \theta \qquad \lambda_d = 0 \ \square \tag{3.14}$$

Thus the expression

$$\det{}^*(2\pi wT) = (2\pi w)^{d-1}. \tag{3.15}$$

4 Tables

Tables 9 to 16 contain the squared bias diagnostic for the additive-log-ratio simulation experiment for each signal-to-noise ratio (SNR).

Table 9: Table of bias for the additive-log-ratio model with SNR of 0.5 and d of 45.

Method	ρ	Squared Bias
Lasso	0	0.868 ± 0.406
Lin	0	0.821 ± 0.39
Bates	0	0.842 ± 0.547
VB	0	0.780 ± 0.409
Lasso	0.2	1.160 ± 0.164
Lin	0.2	1.167 ± 0.444
Bates	0.2	1.047 ± 0.612
VB	0.2	1.022 ± 0.751
Lasso	0.4	1.873 ± 0.587
Lin	0.4	2.096 ± 0.825
Bates	0.4	1.739 ± 0.975
VB	0.4	2.146 ± 1.486

Table 10: Table of bias for the additive-log-ratio model with SNR of 0.5 and d of 200.

Method	ρ	Squared Bias
Lasso	0	1.386 ± 0.466
Lin	0	1.579 ± 0.465
Bates	0	1.201 ± 0.807
VB	0	1.461 ± 0.511
Lasso	0.2	2.010 ± 0.719
Lin	0.2	2.477 ± 0.790
Bates	0.2	1.764 ± 1.425
VB	0.2	2.070 ± 0.624
Lasso	0.4	3.041 ± 0.979
Lin	0.4	4.008 ± 1.359
Bates	0.4	2.798 ± 2.226
VB	0.4	4.354 ± 2.960

Table 11: Table of bias for the additive-log-ratio model with SNR of 0.83 and d of 45.

Method	ρ	Squared Bias
Lasso	0	0.313 ± 0.136
Lin	0	0.297 ± 0.123
Bates	0	0.183 ± 0.179
VB	0	0.281 ± 0.161
Lasso	0.2	0.409 ± 0.164
Lin	0.2	0.390 ± 0.161
Bates	0.2	0.313 ± 0.259
VB	0.2	0.233 ± 0.119
Lasso	0.4	0.702 ± 0.111
Lin	0.4	0.757 ± 0.342
Bates	0.4	0.581 ± 0.396
VB	0.4	0.397 ± 0.182

Table 12: Table of bias for the additive-log-ratio model with SNR of 0.83 and d of 200.

Method	ρ	Squared Bias
Lasso	0	0.617 ± 0.209
Lin	0	0.734 ± 0.257
Bates	0	0.361 ± 0.307
VB	0	0.531 ± 0.047
Lasso	0.2	0.808 ± 0.306
Lin	0.2	0.973 ± 0.336
Bates	0.2	0.361 ± 0.346
VB	0.2	0.687 ± 0.240
Lasso	0.4	1.55 ± 0.459
Lin	0.4	1.972 ± 0.518
Bates	0.4	0.700 ± 0.518
VB	0.4	1.017 ± 0.569

Table 13: Table of bias for the additive-log-ratio model with SNR of 1.67 and d of 45.

Method	ρ	Squared Bias
Lasso	0	0.118 ± 0.061
Lin	0	0.122 ± 0.045
Bates	0	0.041 ± 0.043
VB	0	0.081 ± 0.044
Lasso	0.2	0.079 ± 0.041
Lin	0.2	0.075 ± 0.036
Bates	0.2	0.035 ± 0.037
VB	0.2	0.101 ± 0.048
Lasso	0.4	0.165 ± 0.077
Lin	0.4	0.176 ± 0.066
Bates	0.4	0.043 ± 0.046
VB	0.4	0.086 ± 0.044

Table 14: Table of bias for the additive-log-ratio model with SNR of 1.67 and d of 200.

Method	ρ	Squared Bias
Lasso	0	0.141 ± 0.061
Lin	0	0.131 ± 0.016
Bates	0	0.059 ± 0.016
VB	0	0.174 ± 0.014
Lasso	0.2	0.187 ± 0.006
Lin	0.2	0.218 ± 0.005
Bates	0.2	0.100 ± 0.001
VB	0.2	0.188 ± 0.003
Lasso	0.4	0.257 ± 0.010
Lin	0.4	0.287 ± 0.010
Bates	0.4	0.134 ± 0.065
VB	0.4	0.204 ± 0.001

Table 15: Table of bias for the additive-log-ratio model with SNR of 2.5 and d of 45.

Method	ρ	Squared Bias
Lasso	0	0.051 ± 0.022
Lin	0	0.052 ± 0.020
Bates	0	0.020 ± 0.022
VB	0	0.077 ± 0.029
Lasso	0.2	0.039 ± 0.185
Lin	0.2	0.037 ± 0.017
Bates	0.2	0.019 ± 0.020
VB	0.2	0.097 ± 0.027
Lasso	0.4	0.107 ± 0.032
Lin	0.4	0.176 ± 0.066
Bates	0.4	0.043 ± 0.041
VB	0.4	0.118 ± 0.049

Table 16: Table of bias for the additive-log-ratio model with SNR of 2.5 and d of 200.

Method	ρ	Squared Bias
Lasso	0	0.062 ± 0.061
Lin	0	0.059 ± 0.021
Bates	0	0.044± 0.054
VB	0	0.061 ± 0.001
Lasso	0.2	0.086 ± 0.028
Lin	0.2	0.099 ± 0.014
Bates	0.2	0.014 ± 0.001
VB	0.02	0.131 ± 0.002
Lasso	0.4	0.121 ± 0.042
Lin	0.4	0.134 ± 0.043
Bates	0.4	0.026 ± 0.024
VB	0.4	0.175 ± 0.002

5 Figures

Figure S2 is the full DAG for the CAVI-MC model.Figures S3 to S5 are the ROC curves for the CAVI-MC in the simulation study at signal-to-noise ratio of 0.83 and 1.67, for $d = 45$ and $d = 200$. The frequentist compositional approaches (lasso, symmetric alr, two-stage log-ratio lasso and selbal) are each represented by a dot. The ROC curve illustrates the true positive and false positive rate when thresholding $\mathbb{E}_q[\boldsymbol{\xi}|\mathbf{y}] > u$ at decreasing values of u. The CAVI-MC purple dot identifies thresholding for feature selection at $u = 0.5$.

Figures S6 and S7 are the plots of the ELBO for each CAVI-MC model applied to the data from the "Know your Heart" cross-sectional study of cardiovascular disease.

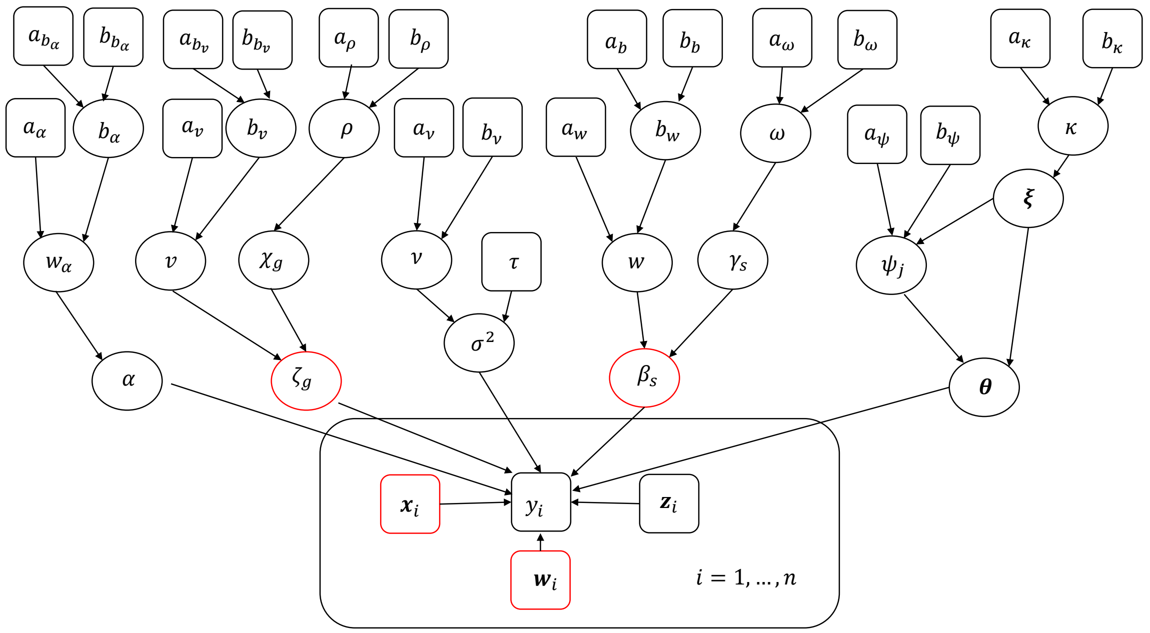

Figure S2: The full DAG of the CAVI-MC model. A square block indicates an element of the model which is fixed; either data via the design matrix or the response, or a hyperparameter of the hyperprior. A circle indicates a random element in the model. A red outline at the lowest level of the DAG highlights a parameter or design matrix omitted in the simulation study. Vectors are in bold.

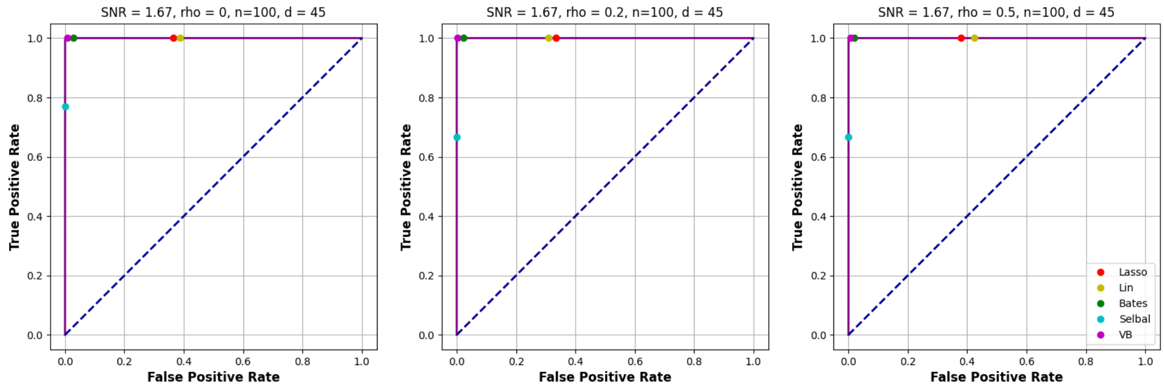

Figure S3: Plot of the ROC curves for the CAVI-MC for a SNR of 1.67 for each value of ρ for $d = 45$.

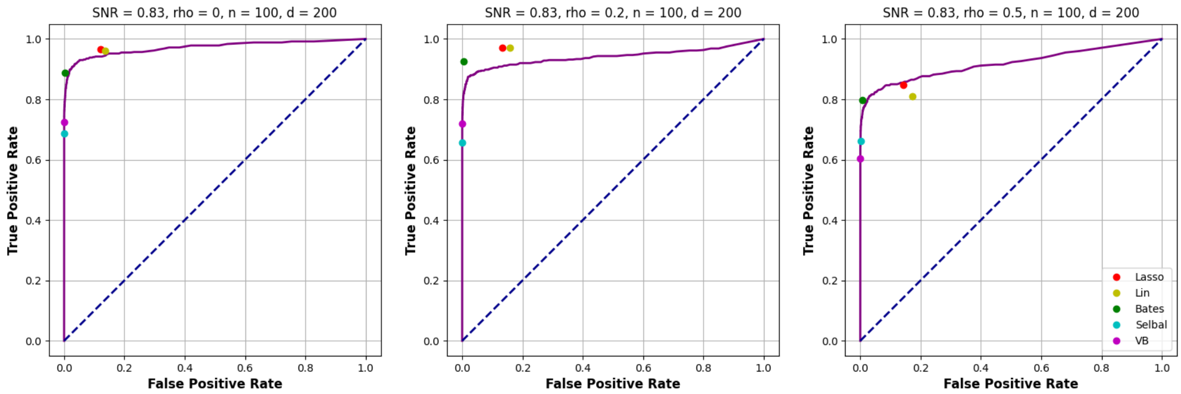

Figure S4: Plot of the ROC curves for the CAVI-MC for a SNR of 0.83 for each value of ρ for $d = 200$.

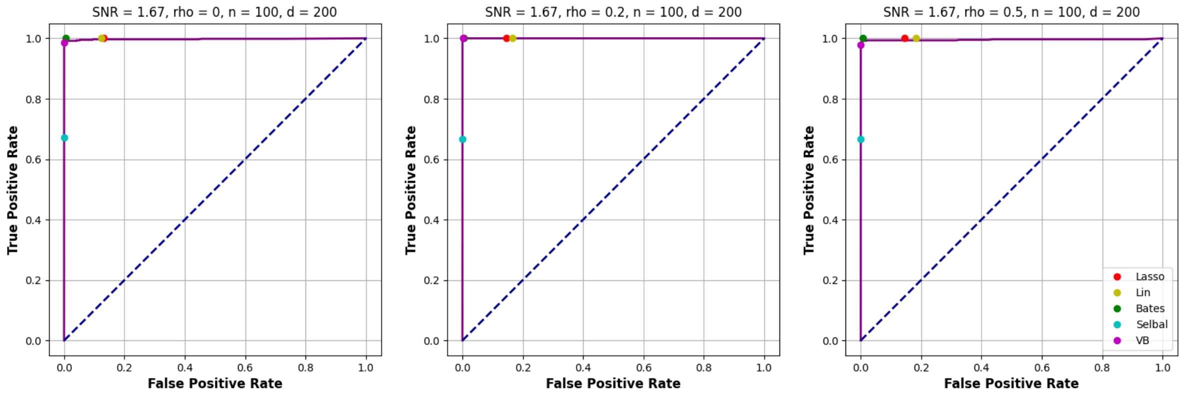

Figure S5: Plot of the ROC curves for the CAVI-MC for a SNR of 1.67 for each value of ρ for $d = 200$.

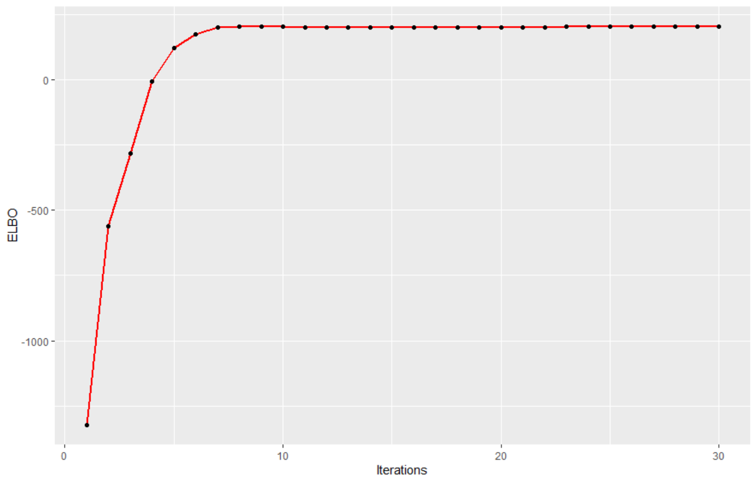

Figure S6: Plot of the ELBO against iterations for the CAVI-MC applied to the "Know Your Heart" data set with the microbiome grouped at the genus level. 30 iterations are performed, with 30,000 between state space moves by the RJMCMC after 4 iterations. The approximate straight line after only 7 iterations implies that the model has reached convergence. Despite the MCMC component removing the monotonic properties of the ELBO, the fluctuations are relatively small.

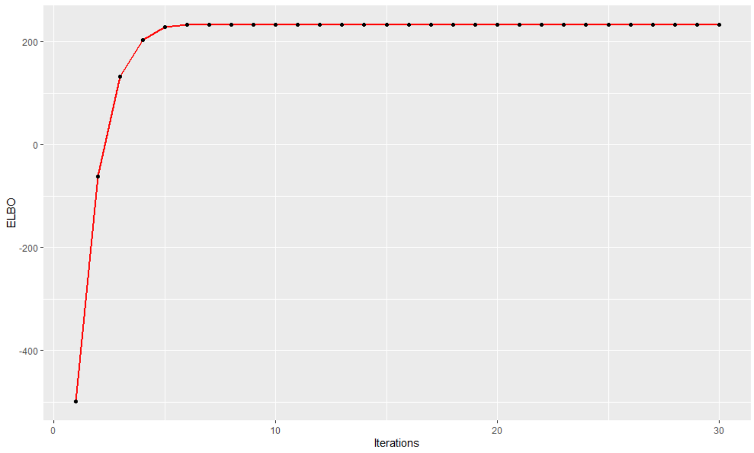

Figure S7: Plot of the ELBO against iterations for the CAVI-MC applied to the "Know Your Heart" data set with the microbiome grouped at the phylum level. 30 iterations are performed, with 30,000 between state space moves by the RJMCMC after 4 iterations. The approximate straight line after only 5 iterations implies that the model has reached convergence. Despite the MCMC component removing the monotonic properties of the ELBO, the fluctuations are relatively small.
